# Tumor cell–intrinsic stress states drive sensitivity to CAR T cell therapy in pancreatic cancer

**DOI:** 10.1126/sciadv.aeh8973

**Published:** 2026-10-02

**Authors:** Julia Fröse, Evelyn Chen, Charles A. Whittaker, Paul Leclerc, Sean Doherty, Adam Langenbucher, Jasmine Shao, Riley D. Hellinger, Daniel Goulet, Tuomas Tammela, Francisco J. Sánchez-Rivera, Michael T. Hemann

**Affiliations:** ^1^ The David H. Koch Institute for Integrative Cancer Research, Cambridge, MA 02142, USA.; ^2^ Department of Biology, Massachusetts Institute of Technology, Cambridge, MA 02142, USA.; ^3^ Cancer Biology and Genetics Program, Sloan Kettering Institute, Memorial Sloan Kettering Cancer Center, New York, NY 10065, USA.; ^4^ David M. Rubenstein Center for Pancreatic Cancer, Memorial Sloan Kettering Cancer Center, New York, NY 10065, USA.

## Abstract

Chimeric antigen receptor (CAR) T cell therapy has transformed the treatment of hematologic cancers but has shown limited efficacy in solid tumors, including pancreatic ductal adenocarcinoma (PDAC). The cellular and molecular factors that influence CAR T cell therapy response remain largely unknown. By integrating modular in vivo CRISPR screens with single-cell genomics and immunocompetent orthotopic models of PDAC, we uncover oxidative and proteotoxic stress pathways as previously unknown modulators of CAR T cell therapy response. Disruption of stress regulatory genes, particularly *Keap1* and *Slc33a1*, sensitizes PDAC tumors to CAR T cell killing in vivo. Hyperactivation of the Nrf2 pathway by genetic ablation of *Keap1* or endogenous engineering of clinically observed *Keap1* or *Nfe2l2* mutations enhances tumor susceptibility to CAR T cell therapy. Thus, tumor-intrinsic molecular stress phenotypes can induce unexpected cell state–specific vulnerabilities to cell-based immunotherapies. These findings provide a foundation to improve efficacy of CAR T cell therapy in solid malignancies and to better stratify patients with cancer by tumor genotype.

## INTRODUCTION

Pancreatic ductal adenocarcinoma (PDAC) is projected to become the second leading cause of cancer death by 2030 (*1*). PDAC lacks effective treatment options, and innovative strategies for therapeutic intervention are urgently needed (*2*). The use of chimeric antigen receptor (CAR) T cell therapy, in which a patient’s own T cells are engineered to recognize and kill cancer cells, has shown remarkable success in hematological malignancies in recent years (*3*). The adaptation of these therapies to solid tumors such as pancreatic cancer holds great promise (*4*). However, clinical trials for CAR T cell therapy in PDAC have shown limited efficacy in patients (*5*). To date, no universal mechanism underlying the therapeutic success or failure has been identified.

CAR T cells face a host of challenges, especially in solid tumor microenvironments, such as inefficient homing to the tumor site, immunosuppressive tumor microenvironments, and lack of persistence in the tumor due to exhaustion or other factors (*6*). Many strategies for improving CAR T cell therapy efficacy have focused on further engineering CAR T cells to improve their persistence and expansion or prevent exhaustion in patients with cancer (*7*, *8*). However, initial studies examining tumor response to immune checkpoint blockade (ICB) have shown that tumor cell–intrinsic properties can also play important roles in influencing the response to immunotherapy (*9*). Recent studies have identified tumor-intrinsic mechanisms of CAR T cell therapy resistance and disease relapse, including antigen loss (*10*–*12*), impaired death receptor signaling (*13*), and alterations in the interferon-γ (IFN-γ) pathway (*14*–*16*). Nevertheless, to date, no cancer-specific dependencies have been found that can influence CAR T cell therapy response. It also remains unclear whether the cancer alterations that underlie tumor responses to conventional or targeted chemotherapy can similarly affect response to CAR T treatment.

Solid tumors are complex ecosystems of tumor cells, as well as multiple types of infiltrating immune cells, fibroblasts, and endothelial cells (*17*). PDAC tumors are particularly complex malignancies characterized by substantial intra- and intertumoral heterogeneity, as well as an unusually dense fibrotic stroma (*18*, *19*). Recent studies have also shown that PDAC tumors harbor distinct phenotypic states, including classical, basal, and mesenchymal states (*20*–*22*). In addition, different cell states have been shown to modulate response to targeted therapies and cytotoxic T cell killing (*23*, *24*). It is unclear whether there are any tumor cell states in PDAC that are particularly sensitive to CAR T cell treatment.

A number of studies focused on CAR T cell therapy resistance in solid tumors have relied on CRISPR-Cas9 knockout (KO) screens in cell culture, immunodeficient mouse models, or heterotopic locations (*25*, *26*). While these screens have uncovered important biology, in vivo screening in orthotopic locations and in the presence of a fully functional immune system may reveal previously unknown tumor-intrinsic mediators of CAR T cell response. Understanding which tumor-intrinsic mechanisms mediate resistance to immunotherapy in relevant physiological settings is key in designing effective treatments for patients.

To uncover tumor cell–intrinsic mechanisms of resistance to CAR T cell therapy in PDAC, we performed iterative CRISPR-Cas9–based pooled KO screens in a fully immunocompetent, orthotopic model of PDAC treated with CAR T cells. In this physiological setting, we uncovered previously unknown modulators of CAR T cell therapy response. Single-cell RNA sequencing (scRNA-seq) of CAR T cell–treated PDAC tumors revealed that therapy-resistant tumor cells exhibit a metabolic cell state characterized by reduced antioxidant pathway activation, reactive oxygen species (ROS) signaling, and glutathione metabolism driven by the oxidative stress response master regulator Nrf2 (nuclear factor, erythroid 1-like 2). Using these two orthogonal approaches, we show that loss of genes involved in mediating proteotoxic and oxidative stress, particularly *Slc33a1* and *Keap1*, is sufficient to sensitize PDAC tumors to CAR T cell treatment in vivo. Hyperactivation of the Keap1/Nrf2 antioxidant response pathway by genetic ablation of *Keap1* or endogenous engineering of a tumor-derived *Keap1* mutation is sufficient to sensitize PDAC tumors to CAR T cell therapy. This work presents a general strategy to integrate functional genomics with preclinical mouse models to study mechanisms of response and resistance to state-of-the-art cell-based immunotherapies. More broadly, our data indicate a potential avenue for therapeutic synergy by targeting pathways involved in cellular stress states to increase the efficacy of CAR T cell therapy in PDAC.

## RESULTS

### A scalable PDAC model to study CAR T cell therapy resistance

To identify genetic mediators of CAR T cell therapy response in an unbiased manner, we established a murine PDAC model amenable to large-scale CRISPR-Cas9 KO screens and targeting by murine CAR T cells. For this purpose, a PDAC cell line derived from autochthonous pancreatic tumors in the KPC (*LSL-Kras^G12D/+^;LSL-Trp53^R172H/+^;Pdx1-Cre*) mouse model (Fig. 1A) was engineered to express both *firefly luciferase* (Luc) and *SpCas9* (*27*). From this population, a Cas9-expressing (Cas9^+^) PDAC clone with high genome editing efficiency (fig. S1, A and B) was isolated. This clone was further modified to express human CD19 (hCD19) without the intracellular domain (−ICD), as an antigen for CAR T cell treatment (hCD19^+^ PDAC; fig. S1C). We selected hCD19 as a tumor antigen for three reasons. First, it allowed us to use established anti-hCD19 CAR constructs. Second, it narrowed the potential scope of biology emerging from the screen results by eliminating the relevance of genes involved in endogenous antigen presentation. Third, this strategy can be extrapolated to other tumor types that may lack suitable murine CAR T cell targets.

**Fig. 1.**
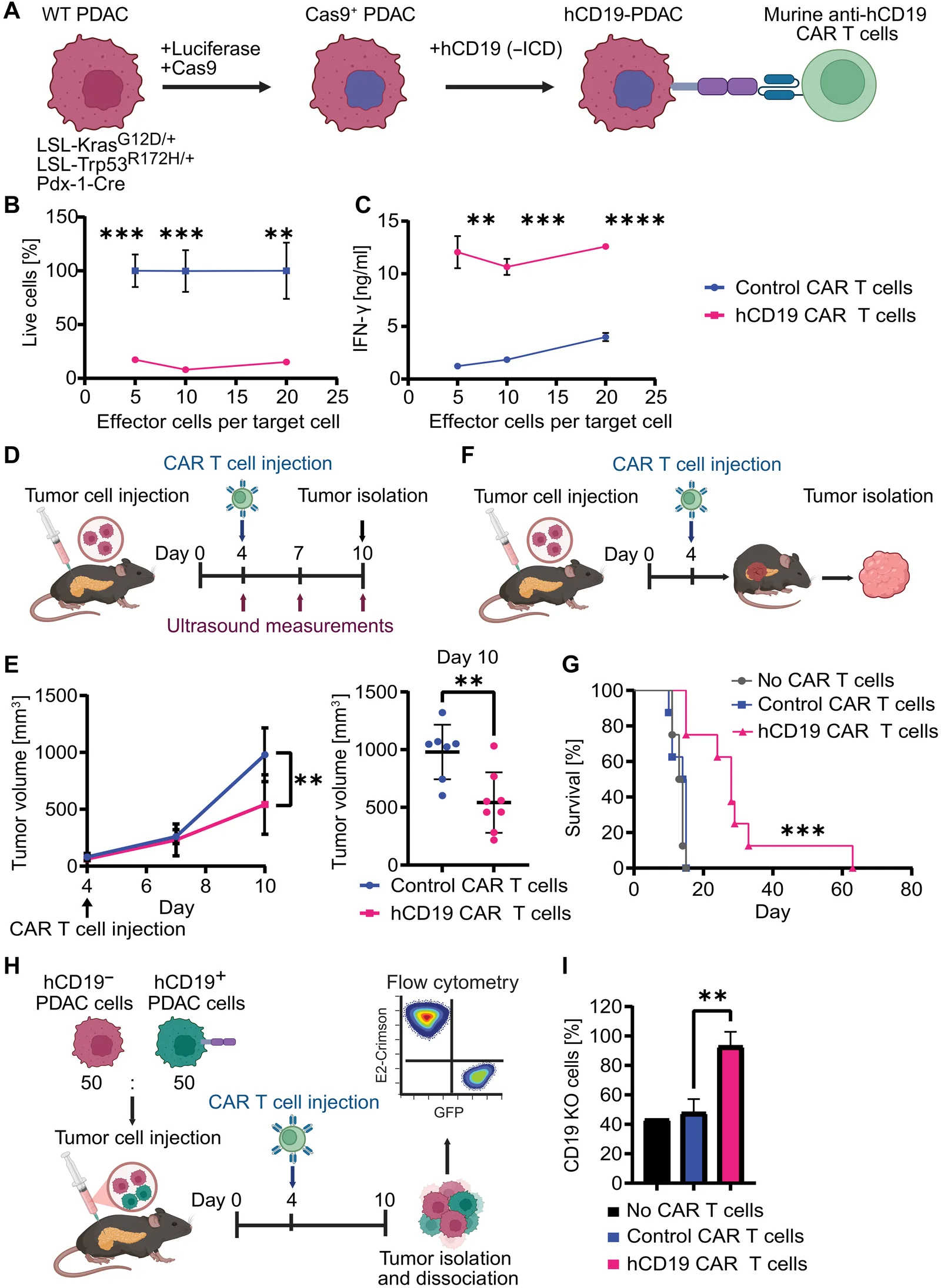
Development of a CRISPR-Cas9 screening–competent PDAC model for CAR T resistance. (**A**) A KPC tumor–derived murine PDAC cell line engineered to stably express Luc, Cas9, and hCD19 (−ICD); single-cell cloned for uniformity.* (**B**) CAR T cell cytotoxicity assay: hCD19 or control (anti-EGFRvIII) CAR T cells cocultured with hCD19^+^ PDAC cells at varying E:T ratios for 24 hours. All conditions analyzed in triplicates; error bars represent means ± SD; unpaired *t* test. (**C**) IFN-γ release from CAR T cells in cytotoxicity assay. All conditions analyzed in triplicates; error bars represent means ± SD; unpaired *t* test. (**D**) Orthotopic model: 6 × 10^5^ PDAC cells injected into C57BL/6 mice and treated with hCD19 (*n* = 8) or control CAR T cells (2.5 × 10^6^; *n* = 7) via intraperitoneal injection on day 4. Tumor volume monitored by ultrasound.* (**E**) Left: tumor volume over time following CAR T treatment. Right: significant tumor reduction on day 10 with hCD19 CAR T cell treatment. Error bars represent means ± SD; Mann-Whitney test (*P* = 0.0059). (**F**) Orthotopic model: 6 × 10^5^ PDAC cells injected into C57BL/6 mice and treated with hCD19, control CAR T cells (2.5 × 10^6^), or no CAR T cells (*n* = 8 per group) via intraperitoneal injection on day 4.* (**G**) Kaplan-Meier curve: survival of mice bearing hCD19^+^ PDAC tumors untreated or treated with hCD19 or control CAR T cells. Mantel-Cox test (*P* = 0.0004). (**H**) Mixed tumor model (1:1 hCD19^+^ GFP^+^ and CD19^−^ E2-Crimson^+^ PDAC cells): 6 × 10^5^ PDAC cells injected into C57BL/6 mice and treated with hCD19 or control CAR T cells (2.5 × 10^6^; *n* = 5 per group) via intraperitoneal injection on day 4; analyzed on day 10 by flow cytometry for GFP^+^ and E2-Crimson^+^ tumor cells.* (**I**) CD19- PDAC tumor cells enrich after hCD19 CAR T cell treatment. Error bars represent means ± SD. Mann-Whitney test (*P* = 0.0079) [not significant (ns) > 0.05, **P ≤ 0.01, ***P ≤ 0.001, and ****P ≤ 0.0001]. *Created in BioRender. Hemann, M. (2026) https://BioRender.com/uu8rpk2.

To determine the feasibility of in vivo screening with hCD19^+^ PDAC in immunocompetent mice, we examined the growth kinetics and tumor burdens of both Luc^+^ PDAC cells and hCD19^+^ PDAC cells. These cells exhibit similar in vitro proliferation rates (fig. S1D). Tumor growth kinetics were also similar between Luc^+^ and hCD19^+^ PDAC cells over a time course of 10 days, indicating that these engineered PDAC cells exhibit no or very little immunogenicity in vivo (fig. S1E). Furthermore, we did not observe any differences in the weight of orthotopic PDAC tumors established from Luc^+^ or hCD19^+^ PDAC cells in either immunocompetent C57BL/6 mice or immunodeficient NOD-SCID/IL2Rg^−/−^ (NSG) mice (fig. S1, F and G). This lack of immunogenicity is in line with previously published work using a Cas9^+^ murine B cell acute lymphoblastic leukemia model in C57BL/6 mice (*14*).

Next, we investigated whether hCD19^+^ PDAC cells are susceptible to CAR T cell killing in vitro. We incubated 10,000 hCD19^+^ PDAC cells with increasing effector-to-target (E:T) ratios of murine CAR T cells targeting EGFRvIII, a control antigen not expressed by our cells, or the model antigen hCD19 for 24 hours (Fig. 1B). We observed efficient killing of PDAC cells at all E:T ratios tested (5:1, 10:1, and 20:1), as well as robust IFN-γ release by hCD19 CAR T cells compared to control CAR T cells (Fig. 1C).

To test whether hCD19^+^ PDAC cells could also be targeted by CAR T cells in vivo, we transplanted hCD19^+^ PDAC cells orthotopically into the pancreas of fully immunocompetent C57BL/6 mice followed by intraperitoneal injection of murine control or hCD19 CAR T cells 4 days later (Fig. 1D). Tumor growth, monitored by ultrasound over the following 10 days, was substantially delayed in mice treated with hCD19 CAR T cells compared to controls (Fig. 1E), and this translated into a survival benefit (Fig. 1, F and G). Despite continued tumor enlargement during treatment, histological analysis revealed extensive necrosis, hemorrhage, and dense immune cell infiltration in CAR T cell–treated tumors (fig. S1H), consistent with robust intratumoral CAR T cell activity.

Having established that hCD19^+^ PDAC cells can be targeted by hCD19 CAR T cells in vivo, we then tested whether CAR T cell treatment can provide the selective pressure required to successfully identify mediators of response and resistance using CRISPR-Cas9 KO screening. A well-established mediator of resistance in patients treated with CAR T cell therapy is loss of the targeted tumor antigen. Therefore, antigen-negative tumor cells should enrich in a mixture with antigen-positive tumor cells when targeted with CAR T cells if the selective pressure is adequate in our model system. To test this, we mixed hCD19 antigen–positive [green fluorescent protein positive (GFP^+^)] PDAC cells with antigen-negative (E2-Crimson^+^) PDAC cells at a 1:1 ratio and treated them with control (EGFRvIII) or hCD19 CAR T cells at an E:T ratio of 10:1 for 24 hours in vitro (fig. S2A). Treatment with hCD19 CAR T cells, but not control CAR T cell treatment or no treatment, led to an increase in antigen-negative PDAC cells relative to antigen-positive PDAC cells (fig. S2B). Similarly, orthotopic injection of a 1:1 mixture of antigen-positive (GFP^+^) and antigen-negative (E2-Crimson^+^) PDAC cells followed by CAR T cell treatment with hCD19 or control CAR T cells leads to an enrichment of antigen-negative PDAC cells over a period of 10 days (Fig. 1, H and I). Together, these results confirm that CAR T cell treatment in our PDAC model can successfully enrich for an established resistance mechanism both in vitro and in vivo. Thus, this selective pressure should similarly enable the identification of previously unidentified mediators of CAR T cell therapy response and resistance using CRISPR-Cas9 KO screening.

For successful in vivo CRISPR-Cas9 KO screening, it is essential to maintain an adequate representation of single guide RNAs (sgRNAs) throughout the experiment (*28*). The size limit of the sgRNA library that can be screened robustly in vivo is influenced by the tumor type, the number of tumor cells that can be safely injected, and the engraftment efficiency of tumor cells in the chosen location. To empirically determine the maximum library size that can be screened in the hCD19^+^ PDAC model, we generated individual hCD19^+^ PDAC cell lines expressing either GFP^+^ or mCherry^+^ to perform limiting GFP dilution assays in vivo. To do so, we established five populations of hCD19^+^ PDAC cells containing decreasing fractions of GFP^+^ cells (50, 1, 0.1, 0.05, or 0.025%) mixed with mCherry^+^ cells and orthotopically transplanted them into C57BL/6 mice (fig. S2C). Each GFP percentage corresponds to the maximal empirical library size that could be used if the corresponding GFP percentage is recovered at the experimental endpoint (fig. S2D). Ten days after transplantation, PDAC tumors were isolated and analyzed by flow cytometry for GFP^+^ PDAC cells. We then compared the percentage of GFP^+^ cells recovered at the end-point (“output”) to the starting percentage of GFP^+^ cells before orthotopic transplantation (“input”) (fig. S2E). In all experimental groups, the empirical output to input ratio was ∼1, indicating robust in vivo maintenance of GFP^+^ PDAC cells throughout the entire experiment. These results indicate that our hCD19^+^ PDAC model could adequately represent a library of ∼4000 sgRNAs in vivo. Together, we have established an immunocompetent, orthotopic PDAC model amenable to CRISPR-Cas9 KO screening in the context of CAR T cell therapy.

### In vivo CRISPR-Cas9 screens reveal modulators of CAR T cell response in PDAC

Next, we performed iterative KO screens using the hCD19^+^ PDAC model system to uncover genetic modulators of CAR T cell response in pancreatic cancer. For this purpose, we made use of the modular SKY CRISPR library that is divided into nonredundant pools that can be individually screened or combined into larger libraries (*14*). This genome-wide library contains four sgRNAs per protein-coding murine gene, divided into 48 pools. Using their initial Kyoto Encyclopedia of Genes and Genomes (KEGG) term in a nonredundant manner, 36% of protein-coding genes could be classified into a KEGG pathway, comprising the first 14 pools and part of pool 15 covering all KEGG pathways, while all other genes were randomly distributed among the remaining pools. Each pool also contained control sgRNAs targeting intergenic regions, olfactory genes, and murine essential genes, and the first pool additionally contained sgRNAs targeting hCD19.

We performed a total of eight independent KO screens covering the first 16 pools of the SKY library (table S1). Guided by our limiting dilution experiments (fig. S2, D and E), we decided to combine and screen two library pools (∼3800 total sgRNAs) in individual mice. Each screen was performed by transplanting 600,000 sgRNA-expressing hCD19^+^ PDAC cells orthotopically into the pancreas of C57BL/6 mice followed by treatment with murine hCD19 CAR T cells or control CAR T cells targeting EGFRvIII 4 days later (Fig. 2A). Each batch of freshly produced murine CAR T cells was functionally validated for antigen-dependent on-target killing efficiency and IFN-γ release in vitro before use in vivo (fig. S3, A to C). Tumors were isolated 10 to 25 days after initial transplantation, when mice showed advanced tumor burden (fig. S3, D to K), dissociated into single-cell suspensions, and subjected to high-throughput sequencing to quantify sgRNA representation. sgRNA coverage was well maintained across all experimental arms compared to prescreen input samples (fig. S4, A and B). To further assess screen quality, we confirmed that many sgRNAs targeting essential genes were significantly depleted in both control and hCD19 CAR T cell–treated groups, whereas those targeting nonessential genes remained unchanged (fig. S5A).

**Fig. 2.**
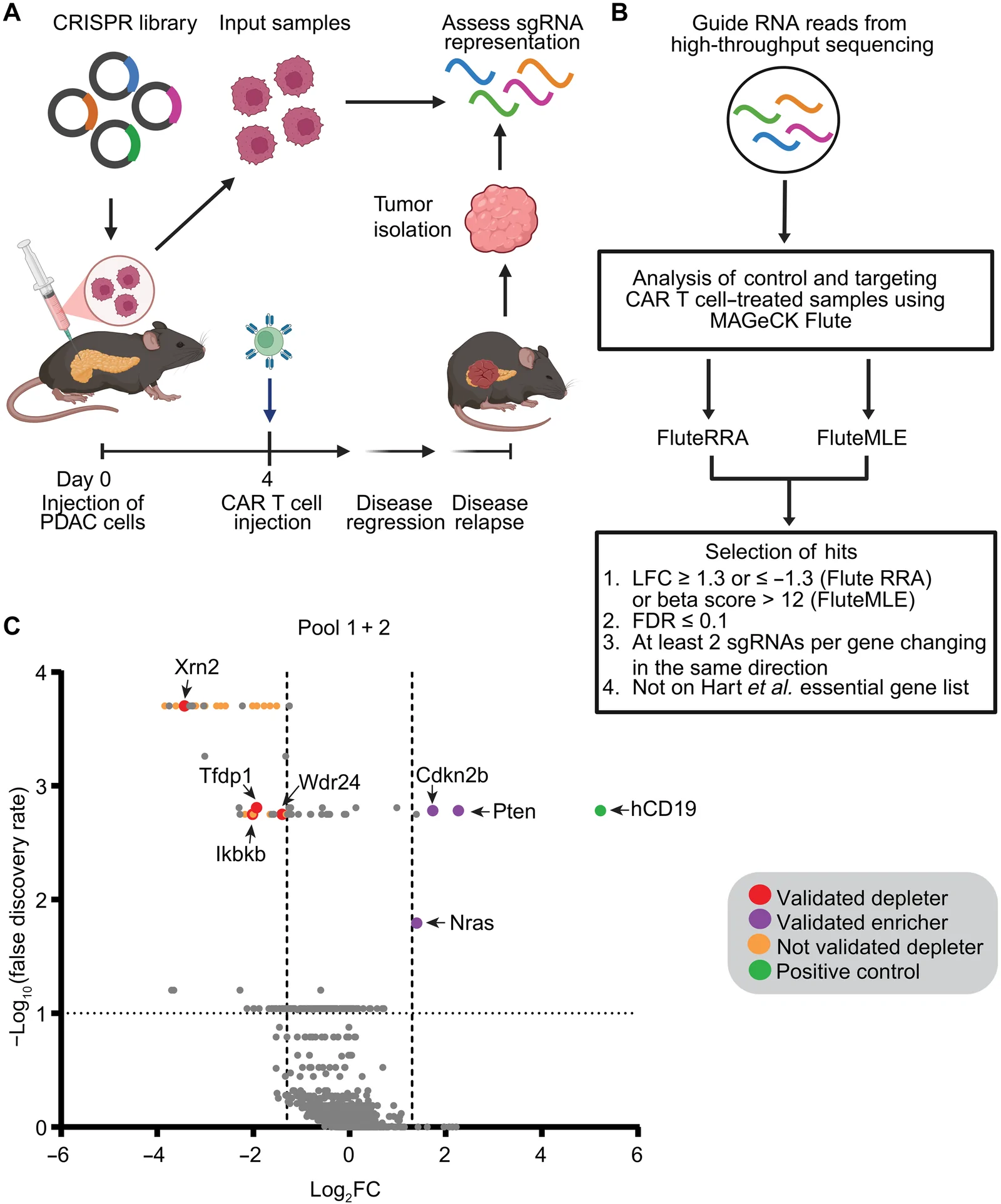
Pooled in vivo CRISPR-Cas9 screens identify candidate genes modulating CAR T response in PDAC. (**A**) PDAC cells containing pooled CRISPR libraries orthotopically injected into C57BL/6 mice and treated with hCD19 or control CAR T cells (*n* = 8 per pool/condition) via intraperitoneal injection on day 4. Tumors were harvested when a humane endpoint was reached. The sgRNA representation in tumors and input samples was quantified by NGS. A total of eight independent screens covered >6000 KEGG-associated genes.* (**B**) Analysis pipeline: sgRNA reads were analyzed using MAGeCK-Flute (RRA and MLE). Hits were defined by effect size (LFC or beta), FDR ≤ 0.1, ≥2 concordant sgRNAs, and exclusion of essential genes.* (**C**) Volcano plot of first screen (library pools 1 and 2), including hCD19 as positive control. “Validated depleter/enricher” refers to genes that were validated in a secondary CRISPR-Cas9 KO screen. “Not validated depleter” refers to genes that were not confirmed as hits in a secondary CRISPR-Cas9 KO screen. *Created in BioRender. Hemann, M. (2026) https://BioRender.com/uu8rpk2.

Next, we used the model-based analysis of genome-wide CRISPR-Cas9 KO (MAGeCK) FluteRRA and FluteMLE algorithms to determine significantly enriched or depleted sgRNAs between control CAR T cell–treated and hCD19 CAR T cell–treated PDAC tumor cells (Fig. 2B) (*29*). Enriched sgRNAs are predicted to target genes whose inactivation mediates resistance to CAR T cell therapy, while depleted sgRNAs target genes whose loss sensitizes PDAC cells to CAR T cell therapy. Genes were considered a “hit” if they fulfilled four rigorous criteria: (i) a log fold change (LFC) of greater than 1.3 or smaller than −1.3 (as determined by FluteRRA) or a beta score higher than 12 (as determined by FluteMLE), (ii) a false discovery rate (FDR) equal to or smaller than 0.1, (iii) at least two of four sgRNAs changing in the same direction, and (iv) not part of the essential gene catalog defined by Hart *et al.* (*30*). sgRNAs targeting the hCD19 CAR T cell antigen showed the strongest enrichment of all sgRNAs in the first screen (Fig. 2C). Across all eight KO screens, a total of 195 genes fulfilled our hit selection criteria and altered the representation of hCD19^+^ PDAC cells following CAR T cell therapy (fig. S6 and table S2). Of these 195 potential CAR T cell therapy modulators, 30 genes were classified as “sensitizers,” and the remaining 147 genes were classified as “resistance” genes according to their LFC through MAGeCK Flute RRA analysis. The remaining 24 genes were included for validation because they were classified as additional “hits” (6 sensitizers and 18 resistance genes) according to MAGeCKs Flute MLE analysis.

A caveat of our pooled screening approach is that it does not enable a ranked comparison between sgRNAs across separate screens. Therefore, we performed a validation screen using a secondary CRISPR KO library containing six sgRNAs targeting each gene previously identified as a hit, as well as 49 essential genes (table S2), along with 90 nontargeting (scrambled), 213 intergenic, and 91 olfactory gene–targeting sgRNAs that exhibited neutral behavior (no enrichment or depletion) in our initial screens. We performed this validation screen in an identical fashion to the primary KO screens. CAR T cell functionality was confirmed in vitro by efficient on-target killing and IFN-γ release (fig. S7, A to C). Tumors were isolated when mice showed advanced tumor burden, 15 to 32 days after initial injection (fig. S7D), and sgRNA representation was quantified using high-throughput sequencing. Compared to prescreen input samples, sgRNA coverage was generally maintained, although some variation was observed, especially in tumors from long-term survivors treated with hCD19 CAR T cells (fig. S7E). As in our primary screens, sgRNAs targeting known essential genes have large negative fold changes relative to nonessential genes in both the control and hCD19 CAR T cell–treated groups compared to their respective prescreen input samples, whereas sgRNAs targeting intergenic regions or nonessential genes were unchanged, confirming screen robustness (fig. S7F).

Bona fide modulators of CAR T cell therapy were determined by assessing the FDR values, LFCs, and both intrascreen (within validation screen) and interscreen (compared to primary screens) consistency of sgRNA behavior (fig. S7G). Of the 195 genes that we screened, 30 genes validated as robust modulators of CAR T cell therapy (Fig. 3A and fig. S8, A to C). Of these genes, 13 sensitized tumor cells to CAR T cell therapy as indicated by consistent enrichment of sgRNAs targeting these genes (Fig. 3B). This group consisted of known tumor suppressor genes (e.g., *Pten*, *Cdkn2b*, and *Eif4g2*) (*31*–*33*) and genes regulating cell proliferation (e.g., Hippo pathway genes *Nf2*, *Taok1*, and *Lats2*) (*34*). Loss of *Pten* has previously been shown to mediate resistance to ICB therapy in multiple tumor types (*35*–*38*). The remaining 17 genes were identified as mediators of resistance to CAR T cell therapy, as indicated by consistent depletion of sgRNAs targeting those genes (Fig. 3B). This set included mediators of autophagy (*Vmp1* and *Wrd24*) and intracellular trafficking (*Vps29*) processes previously shown to mediate immune evasion from T cells (*39*). We also identified known regulators of clathrin-mediated endocytosis (*Aps2s1* and *DynII1*) as resistance genes to CAR T cell therapy (*40*). Unexpectedly, multiple genes identified in our screen were core-metabolic genes (Fig. 3C), encoding enzymes or transporters in canonical metabolic pathways defined by KEGG, Reactome, and GO annotations (*Hmgcs1*, *Sptlc1*, *Slc33a1*, *Fxn*, *Pigs*, *Alg10a*, and *Alg3*) (*41*–*44*). In addition, nine other genes can be classified as metabolism adjacent due to their roles in nutrient sensing, autophagy, and metabolic stress adaptation (table S3). Currently, no tumor-intrinsic alterations in metabolic genes have been described to influence CAR T cell response, highlighting metabolic stress regulation as a previously unrecognized determinant of PDAC susceptibility to CAR T cell therapy.

**Fig. 3.**
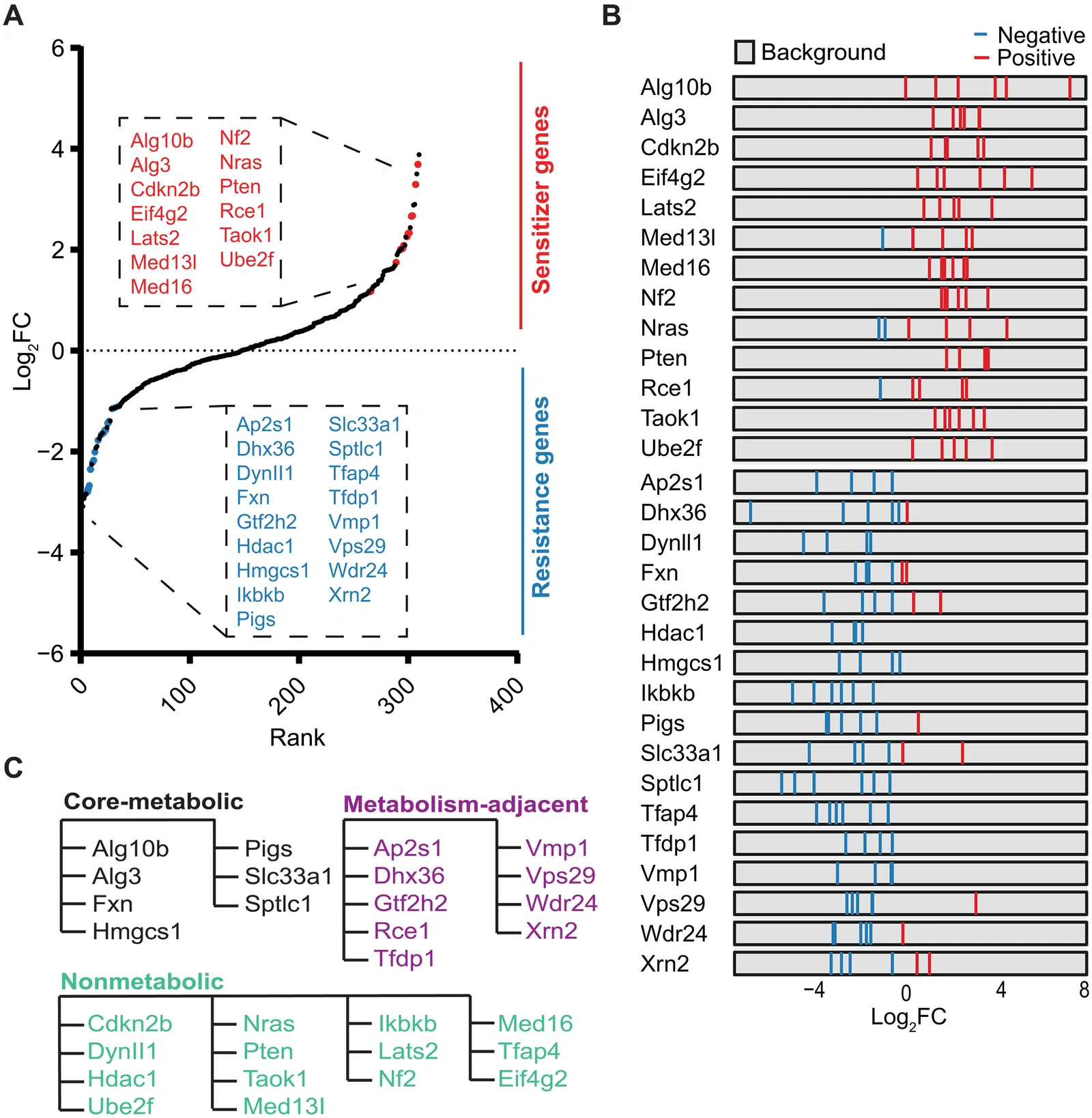
In vivo CRISPR-Cas9 validation screen identifies modulators of CAR T response in PDAC. (**A**) Validation screen: secondary CRISPR library (six sgRNAs per gene) targeting candidates from primary screens under identical conditions; rank plot highlights validated genes. In total, 13 sensitizers and 17 resistance genes were identified. (**B**) Log_2_ fold change of individual sgRNAs for validated genes; enrichment (red) and depletion (blue). (**C**) Classification of modulators of CAR T cell therapy response into core-metabolic, metabolism-adjacent, or nonmetabolic genes using KEGG, Reactome, and GO annotations.

To further validate our screen results, we selected *Sptlc1*, an independently reported regulator of immune evasion in KRAS-driven cancers, for functional validation in vivo (*45*). *Sptlc1* KO significantly prolonged survival following CAR T cell therapy without impairing tumor formation (fig. S9). Having established the validity of the screening strategy, we next focused on the metabolic stress pathways that had not previously been linked to CAR T cell response.

### scRNA-seq of CAR T–treated PDAC reveals Nrf2-driven redox changes

To determine whether CAR T cell therapy similarly selects for distinct metabolic tumor cell states in vivo, we performed scRNA-seq of orthotopic hCD19^+^ PDAC tumors treated with control (EGFRvIII) or hCD19 CAR T cells. For this purpose, we injected hCD19^+^ PDAC cells orthotopically into C57BL/6 mice and treated them with freshly produced murine CAR T cells (Fig. 4A and fig. S10, A to C). Tumors were isolated after 10 days for control CAR T cell–treated mice and after 15 days for hCD19 CAR T cell–treated mice, at which stage all PDAC tumors had reached a similar weight (fig. S10D). Following tumor dissociation into single cells, live cell mixtures were subjected to scRNA-seq. The resulting data were filtered, and 20,516 cells were portioned into clusters of similar cells (fig. S10E; see Materials and Methods). We identified tumor cells by hCD19 and Cas9 expression and annotated stromal and immune cell clusters using well-established genes and gene signatures, such as *Cd19* for B cells and *Cd68* for macrophages (figs. S10F and S11A). In total, we identified 12 transcriptionally distinct clusters consisting of PDAC cells, cells of the innate and adaptive immune system, including CAR T cells (*Cd8a* and GFP expression), endothelial cells, pancreatic stellate cells, and erythroid cells (fig. S11B).

**Fig. 4.**
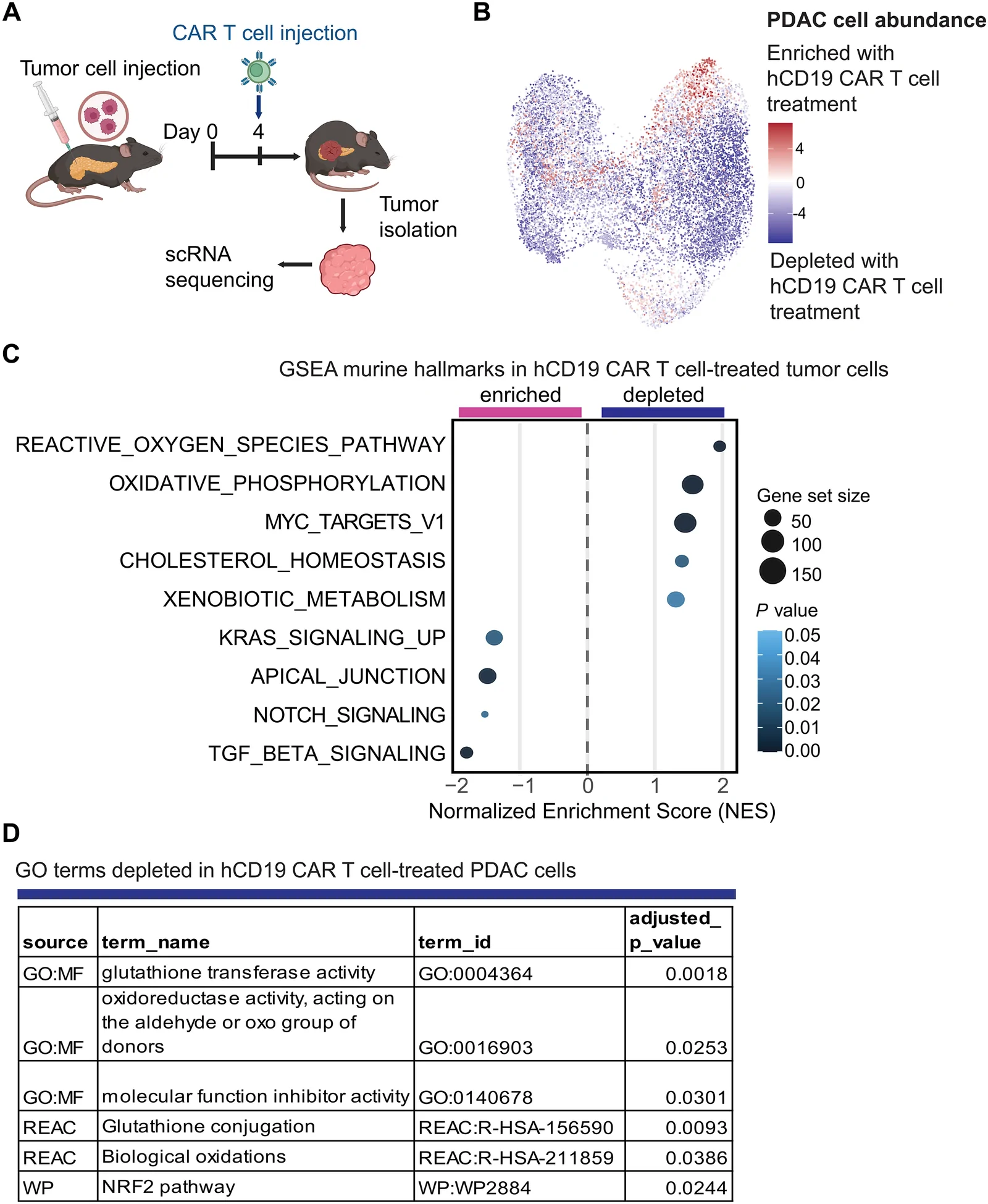
scRNA-seq of CAR T–treated PDAC tumors reveals altered redox states. (**A**) Orthotopic model: 6 × 10^5^ murine PDAC cells injected into C57BL/6 mice and treated with hCD19 or control CAR T cells (2.5 × 10^6^; *n* = 4 per group) via intraperitoneal injection on day 4. Tumors were harvested at ∼1500 mm^3^ (day 10 for control and day 15 for hCD19 CAR T–treated tumors) and analyzed by scRNA-seq. Created in BioRender. Hemann, M. (2026) https://BioRender.com/uu8rpk2. (**B**) UMAP of PDAC cells showing enrichment (red) or depletion (blue) of tumor cells after hCD19 CAR T treatment. (**C**) Dot plot: GSEA of murine hallmark pathways in PDAC cells from hCD19 versus control CAR T–treated tumors. (**D**) GO analysis of top 100 depleted genes in hCD19 versus control CAR T–treated PDAC tumors.

To identify differentially expressed genes between PDAC cells treated with control CAR T cells compared to hCD19 CAR T cells, we isolated the 13,410 PDAC cells in our dataset and performed unsupervised clustering and differential gene expression analysis. Furthermore, we identified differences in the abundance of tumor cells in different regions of the two-dimensional uniform manifold approximation and projection (UMAP) plots between control and hCD19 CAR T cell treatment, showing CAR T cell treatment–specific enrichment and depletion of certain tumor cell clusters (Fig. 4B). To identify biological processes and molecular functions affected in PDAC cells upon treatment with CAR T cells, we performed gene set enrichment analysis (GSEA) on murine hallmark genes. This analysis showed the depletion of ROS, oxidative phosphorylation, *Myc* targets, cholesterol homeostasis, and xenobiotic metabolism pathways in PDAC cells following hCD19 CAR T cell treatment, while signaling pathways such as *Tgf-*β, *Notch*, apical junction, and *Kras* signaling were enriched (Fig. 4C). In parallel, we performed gene ontology (GO) analysis using gProfiler on the top 100 differentially expressed genes and revealed down-regulation of pathways related to oxidative stress in hCD19 CAR T cell–treated PDAC cells, including glutathione metabolism, oxidoreductase activity, biological oxidation, and the Nrf2 pathway (Fig. 4D). Conversely, pathways regulating the interaction of tumor cells with the extracellular matrix were up-regulated in hCD19 CAR T cell–treated PDAC cells (fig. S11C). Together, these results indicate that PDAC cells treated with hCD19 CAR T cells exhibit substantial molecular changes in oxidative stress pathways, including the Nrf2 antioxidant response.

### Nrf2-activated PDAC cells are eliminated by CAR T cell therapy

The Nrf2 antioxidant response element (ARE) signaling pathway plays a key role in the cellular defense against oxidative or electrophilic stress (*46*). The cytosolic protein Kelch-like ECH-associated protein 1 (Keap1) acts as the central negative regulator of this pathway by targeting Nrf2 for proteasomal degradation (*47*). Mutations in *Keap1* that lead to Nrf2 hyperactivation have been implicated in mediating resistance to ICB therapy in lung adenocarcinoma (LUAD) (*48*). This resistance can be overcome by inhibition of glutaminase, a key enzyme that produces glutamate which is required for the glutathione redox system (*49*). To further analyze the connection between the Nrf2 pathway and CAR T cell therapy, we investigated the expression of a gene signature of murine Nrf2-responsive genes in PDAC cells following hCD19 CAR T cell treatment (Fig. 5A and table S4). Consistent with our previous results, expression of murine Nrf2-responsive genes was notably reduced in PDAC cells that survived hCD19 CAR T cell treatment compared to control CAR T cell treatment. Thus, we hypothesized that PDAC cells with Nrf2 pathway activation may show increased sensitivity to CAR T cell treatment. To test this, we leveraged a previously described *Nrf2* 8xARE-GFP reporter for which GFP expression serves as a readout of *Nrf2* transcriptional activity (*50*). Following transduction of wild-type (WT) PDAC cells with the *Nrf2* 8xARE-GFP reporter (*Nrf2* reporter), we validated reporter activity by treating these cells with tert-butylhydroquinone (tBHQ), an established *Nrf2* activator (*51*). We observed a robust, dose-dependent induction of GFP expression in vitro, thereby validating the assay (fig. S12, A and B). Next, we orthotopically transplanted *Nrf2* reporter PDAC cells into C57BL/6 mice and treated them with either control (EGFRvIII) or murine mesothelin (mmeso) CAR T cells (Fig. 5B) (*52*). Ten days later, we isolated the tumors and analyzed *Nrf2* reporter activity via GFP expression in PDAC cells. Notably, *Nrf2* reporter activity was significantly lower in PDAC cells isolated from tumors treated with mmeso CAR T cells compared to those isolated from tumors treated with control CAR T cells at the same time point (Fig. 5C and fig. S12C). The PDAC tumors treated with mmeso CAR T cells were significantly smaller than control CAR T cell treated tumors on the same day (fig. S12D). To match these data to the time points of our previous scRNA-seq experiment, we isolated PDAC tumors treated with mmeso CAR T cells after 15 days as well, at which point the tumors had reached a similar size as control CAR T cell treated tumors on day 10 (fig. S12D). We observed an even further decrease of *Nrf2* reporter activity in mmeso CAR T cell–treated tumors on day 15 (Fig. 5C). These results indicate that PDAC cells with high Nrf2 pathway activity may be hypersensitive to, and selectively eliminated by, CAR T cell therapy in vivo. This stands in contrast to ICB in which Nrf2 hyperactivation in cancer cells is associated with therapy resistance (*53*).

**Fig. 5.**
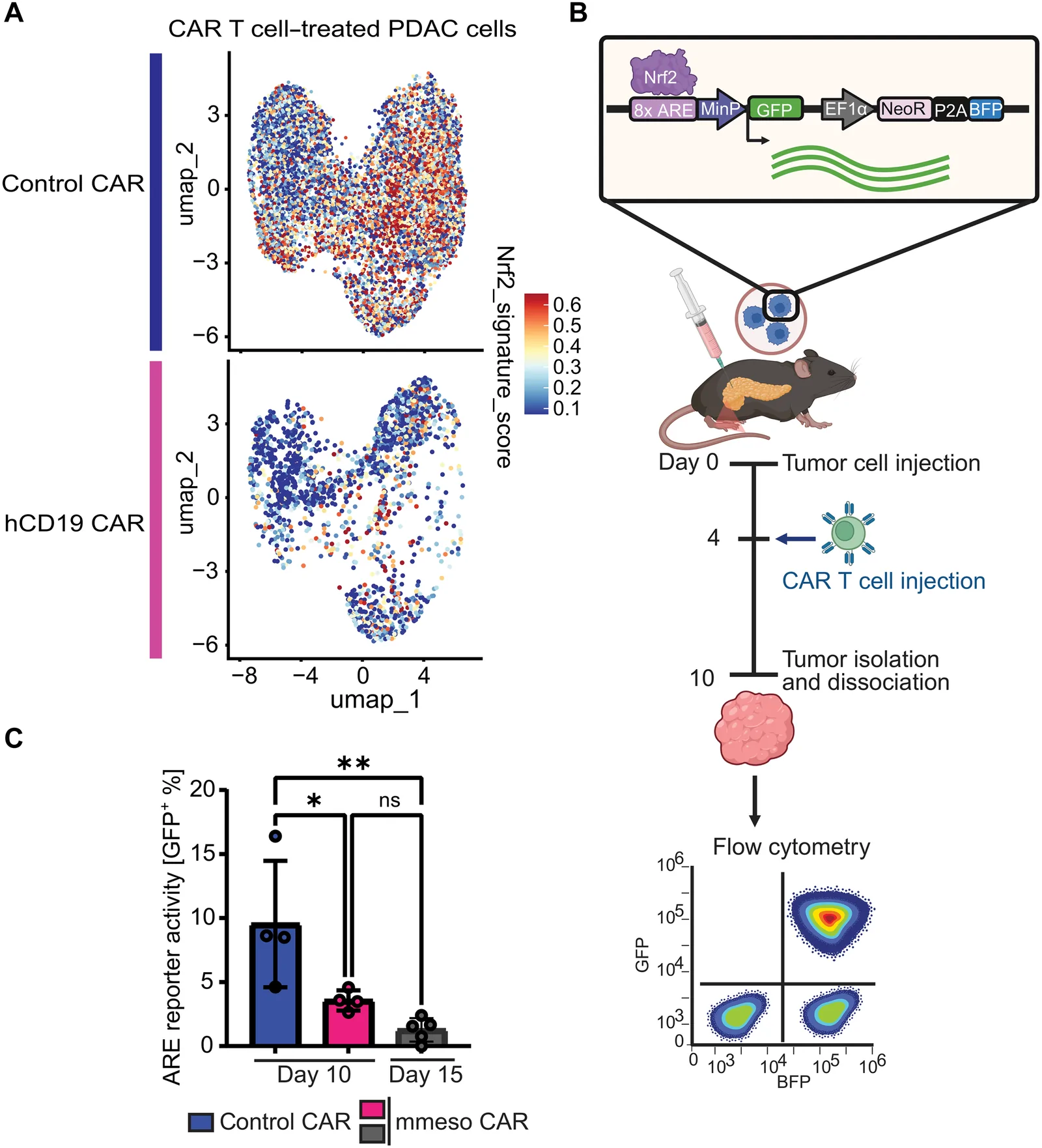
Nrf2-hyperactivated cells are depleted by CAR T cell therapy in vivo. (**A**) UMAP showing the expression of a Nrf2-responsive gene signature (murine orthologs of the human NRF2 Wikipathway) in PDAC cells from hCD19 versus control CAR T–treated tumors. Nrf2-responsive gene expression is lower in hCD19 CAR T–treated PDAC cells. (**B**) Orthotopic model: 6 × 10^5^ PDAC cells expressing an Nrf2 8xARE-GFP reporter injected into C57BL/6 mice and treated with mmeso or control CAR T cells (5 × 10^6^; *n* = 4 to 5 per group) via intraperitoneal injection on day 4. Tumors harvested on day 10 or 15. Nrf2 activity (GFP) in PDAC cells analyzed by flow cytometry. Created in BioRender. Hemann, M. (2026) https://BioRender.com/uu8rpk2. (**C**) Nrf2 activity (GFP) in PDAC cells is reduced following mmeso CAR T treatment. Error bars represent means ± SD. Ordinary one-way analysis of variance (ANOVA) [not significant (ns) > 0.05, *P ≤ 0.05, and **P ≤ 0.01].

### *Slc33a1* loss and Nrf2 hyperactivation are synthetically lethal in PDAC

Our CRISPR-Cas9 KO screening and single-cell transcriptome analysis of PDAC cells treated with CAR T cell therapy converge on metabolic genes as critical modulators of CAR T cell therapy response. Among the screen hits, *Slc33a1* was particularly intriguing because previous work identified a synthetic lethal interaction with *Keap1*, a key negative regulator of Nrf2 signaling in LUAD (*54*). We wanted to confirm that Slc33a1 was similarly linked to the tolerance of Nrf2-hyperactive states in PDAC.

To do so, we performed in vitro competition assays with hCD19^+^ PDAC cells containing a control (*Olfr402*) KO, *Slc33a1* KO, or *Slc33a1* reexpression in combination with *Keap1* KO (fig. S12E). PDAC cells with *Slc33a1*/*Keap1* double KO depleted over time compared to PDAC cells with single *Slc33a1* KO, whereas *Olfr402/Keap1* double-KO cells did not deplete in comparison to *Olfr402* KO PDAC cells (fig. S12F). At the experimental endpoint, the remaining PDAC cells in the *Keap1/Slc33a1* double-KO population were selected against *Keap1* loss (fig. S12G). These results demonstrate that the *Keap1*/*Slc33a1* synthetic lethal interaction is conserved across different types of tumor cells.

### Loss of *Slc33a1* sensitizes PDAC tumors to CAR T cell therapy in vivo

Given that (i) Nrf2-high PDAC cells may be selectively sensitive to CAR T cells, (ii) *Keap1* is a negative regulator of *Nrf2* response, and (iii) there is a conserved genetic link between *Slc33a1* and *Keap1*, we hypothesized that *Slc33a1* loss and *Keap1* loss might impose different intrinsic stresses in PDAC cells that converge on increased sensitivity to CAR T cell therapy. To test this hypothesis, we set out to first validate *Slc33a1* as a resistance mediator to CAR T cell therapy.

*Slc33a1* encodes for the solute carrier family member 1 protein, an acetyl-coenzyme A and oxidized glutathione transporter located in the endoplasmic reticulum (ER) membrane (*55*, *56*). Its function is essential for the redox balance and lysine acetylation of proteins in the ER, and both loss of function and overactivation of *Slc33a1* have been implicated in various diseases (*57*, *58*). In pancreatic acinar cells, *Slc33a1* KO has been shown to result in ER stress, fibrosis, and inflammation (*59*). For validation, we injected hCD19^+^ PDAC cells with *Slc33a1* KO (Fig. 6A) or control (*Olfr402*) KO into C57BL/6 mice and treated them with murine control (EGFRvIII) or hCD19 CAR T cells (Fig. 6B). We observed a statistically significant extension of survival in mice injected with *Slc33a1* KO hCD19^+^ PDAC cells compared to control KO PDAC cells treated with hCD19 CAR T cells (Fig. 6C). Mice injected with *Slc33a1* KO hCD19^+^ PDAC cells treated with hCD19 CAR T cells ultimately succumbed to disease; however, end-stage *Slc33a1* KO tumors were significantly larger compared to all other experimental groups and showed extensive immune cell infiltration, hemorrhage, and necrosis (Fig. 6, D and E). These features are suggestive of pseudo-progression, a clinically observed phenomenon during immunotherapy in which immune-mediated inflammation and cell death lead to apparent increases in tumor volume despite effective antitumor responses (*60*–*62*). In this context, the absence of tumor regression may not reflect therapeutic failure but rather that tumors reach a size incompatible with survival before meaningful shrinkage can occur. While we cannot definitively distinguish pseudo-progression from true progression in this model, the observed histological changes and survival benefit support this as a potential explanation.

**Fig. 6.**
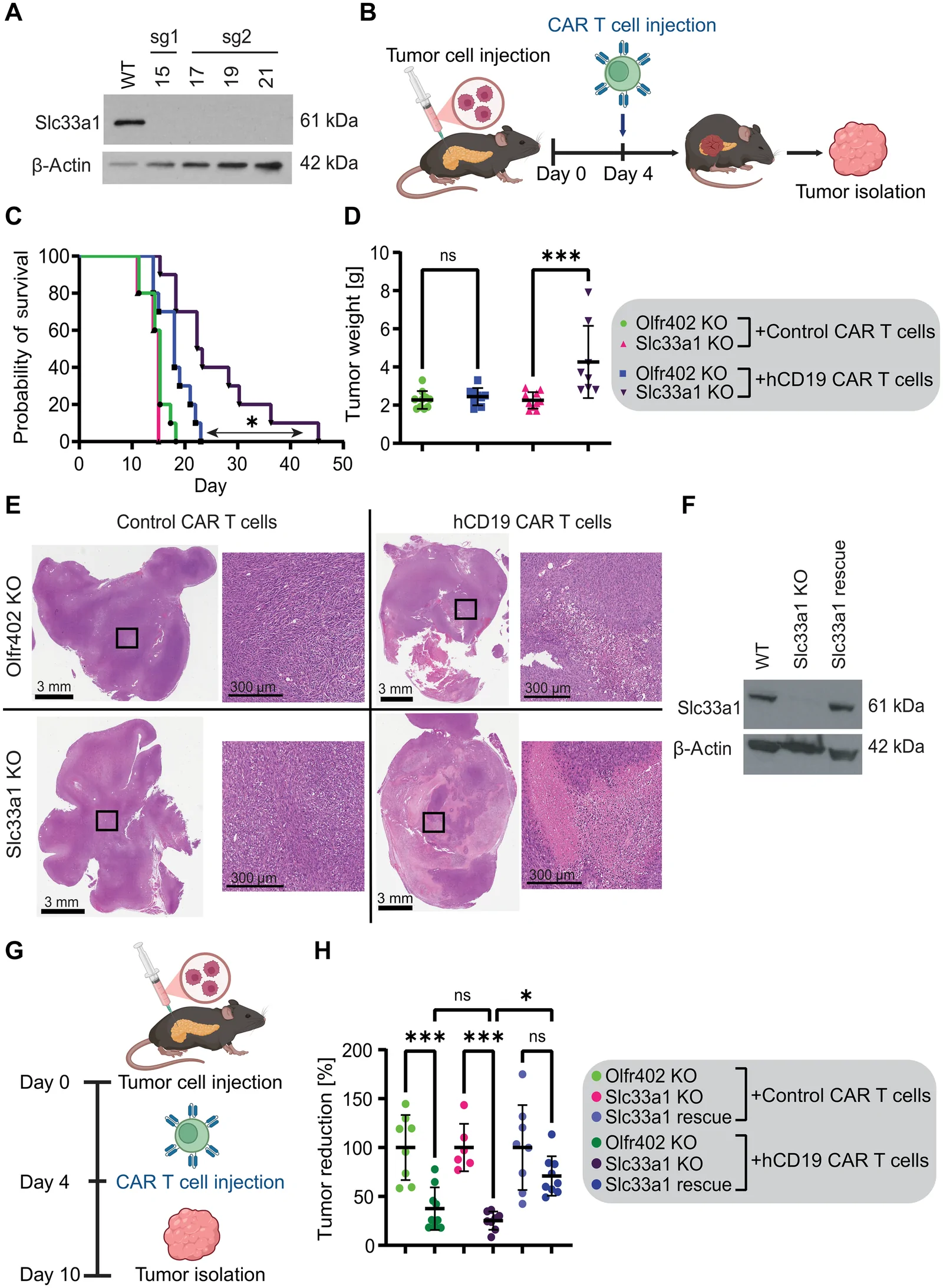
Loss of *Slc33a1* sensitizes to CAR T cell therapy in vivo. (**A**) Western blot confirming *Slc33a1* KO in multiple hCD19^+^ PDAC single-cell clones using two independent sgRNAs. (**B**) Orthotopic model: 6 × 10^5^ PDAC cells (control KO or Slc33a1 KO) injected into C57BL/6 mice and treated with hCD19 or control (EGFRvIII) CAR T cells (5 × 10^6^; *n* = 10 per group) via intraperitoneal injection on day 4. Tumors were harvested when a humane endpoint was reached.* (**C**) Kaplan-Meier curve: *Slc33a1* KO tumors show extended survival following hCD19 CAR T treatment. Mantel-Cox test (*P* = 0.0123). (**D**) Tumor weight at morbidity: *Slc33a1* KO tumors treated with hCD19 CAR T cells are significantly larger. Error bars represent means ± SD. Ordinary one-way ANOVA (*P* = 0.0006). (**E**) Hematoxylin and eosin–stained tumor sections (control KO versus *Slc33a1* KO; ± CAR T treatment); right shows magnified regions. Increased necrosis observed in *Slc33a1* KO tumors following hCD19 CAR T treatment. (**F**) Western blot confirming *Slc33a1* reconstitution in KO PDAC cells. (**G**) Orthotopic model: 6 × 10^5^ PDAC cells (control KO, *Slc33a1* KO, or *Slc33a1* rescue) injected into C57BL/6 mice and treated with hCD19 or control CAR T cells (5 × 10^6^; *n* = 6 to 8 per group) via intraperitoneal injection on day 4. Tumors harvested on day 10.* (**H**) Relative tumor reduction following hCD19 CAR T treatment across genotypes. Error bars represent means ± SD. Ordinary one-way ANOVA [not significant (ns) > 0.05, *P ≤ 0.05, and ***P ≤ 0.001]. *Created in BioRender. Hemann, M. (2026) https://BioRender.com/uu8rpk2.

Next, we tested whether *Slc33a1* loss in tumor cells causes any changes in the tumor microenvironment that may contribute to their increased susceptibility to CAR T cell therapy. To control for potential clonal effects occurring in *Slc33a1* KO cells, we expressed a Cas9-resistant version of *Slc33a1* in *Slc33a1* KO cells to create *Slc33a1* rescue cells (Fig. 6F). Then, we injected hCD19^+^ PDAC cells with control (*Olfr402*) KO, *Slc33a1* KO, or *Slc33a1* rescue orthotopically into C57BL/6 mice, treated them with murine control (EGFRvIII) or hCD19 CAR T cells, and isolated the tumors after 10 days (Fig. 6G). On-target (hCD19) CAR T cell therapy consistently reduced the tumor weight of control KO and *Slc33a1* KO PDAC tumors, but not of *Slc33a1* rescue tumors (Fig. 6H and fig. S13A). Here, *Slc33a1* rescue tumors were the most resistant to hCD19 CAR T cell therapy.

We subsequently dissociated these tumors and analyzed their tumor cell and immune cell content by flow cytometry. No differences in tumor cell content or CD45^+^ immune cells, including T cell subsets, B cells, natural killer (NK) cells, and myeloid cell populations, were observed between control KO, *Slc33a1* KO, and *Slc33a1* rescue tumors treated with control CAR T cells (fig. S13, B to L). Similarly, there were no significant changes in immune cell populations between control KO tumors and *Slc33a1* KO tumors treated with hCD19 CAR T cells. The only changes in immune cell subsets we observed were caused by hCD19 CAR T cell treatment and were independent of, *Slc33a1* status, suggesting that *Slc33a1* loss does not alter the immune tumor microenvironment.

Given that the enhanced sensitivity of *Slc33a1*-deficient cells is not caused by the immune tumor microenvironment, we wanted to explore cell-intrinsic transcriptome changes caused by *Slc33a1* KO in tumor cells in vivo. Therefore, we injected hCD19^+^ PDAC cells with control (*Olfr402*) KO or *Slc33a1* KO orthotopically into C57BL/6 mice and treated them with murine control (EGFRvIII) or hCD19 CAR T cells. After 12 days, we harvested the tumors, isolated E2-Crimson^+^ tumor cells by fluorescence-activated cell sorting, and performed bulk transcriptome profiling. Differential gene expression analysis between control KO and *Slc33a1* KO tumor cells followed by GSEA revealed a depletion in inflammatory response signaling, such as IFN-γ, IFN-α, and interleukin-6–Janus kinase–signal transducer and activator of transcription (IL-6/JAK/STAT) signaling, but an enrichment in the UPR in PDAC cells with *Slc33a1* loss (fig. S14, A to C). This is in line with previous studies showing that *Slc33a1* loss leads to ER stress and the induction of the UPR (*54*, *59*).

### Nrf2 hyperactivation sensitizes PDAC tumors to CAR T cell therapy in vivo

Having verified that *Slc33a1* loss sensitizes PDAC cells to CAR T cell therapy, we tested whether *Keap1* loss alone could sensitize PDAC cells to CAR T cell therapy. To this end, we orthotopically transplanted hCD19^+^ PDAC cells with control (*Olfr402*) or previously generated *Keap1* KO (sg2) into C57BL/6 mice and treated them with control (EGFRvIII) or hCD19 CAR T cells (Fig. 7, A and B). Consistent with our hypothesis, mice harboring *Keap1* KO PDAC tumors treated with hCD19 CAR T cells showed a significant survival advantage compared to mice with control PDAC tumors treated with hCD19 CAR T cells (*P* = 0.0086; Fig. 7C). Of note, while *Keap1*-targeting sgRNAs were not present in our initial screening library, the orthogonal use of scRNA-seq and the genetic interaction between *Keap1* and *Slc33a1* allowed us to identify a human cancer gene and a mechanistic cellular redox state that modulates response to CAR T cell therapy.

**Fig. 7.**
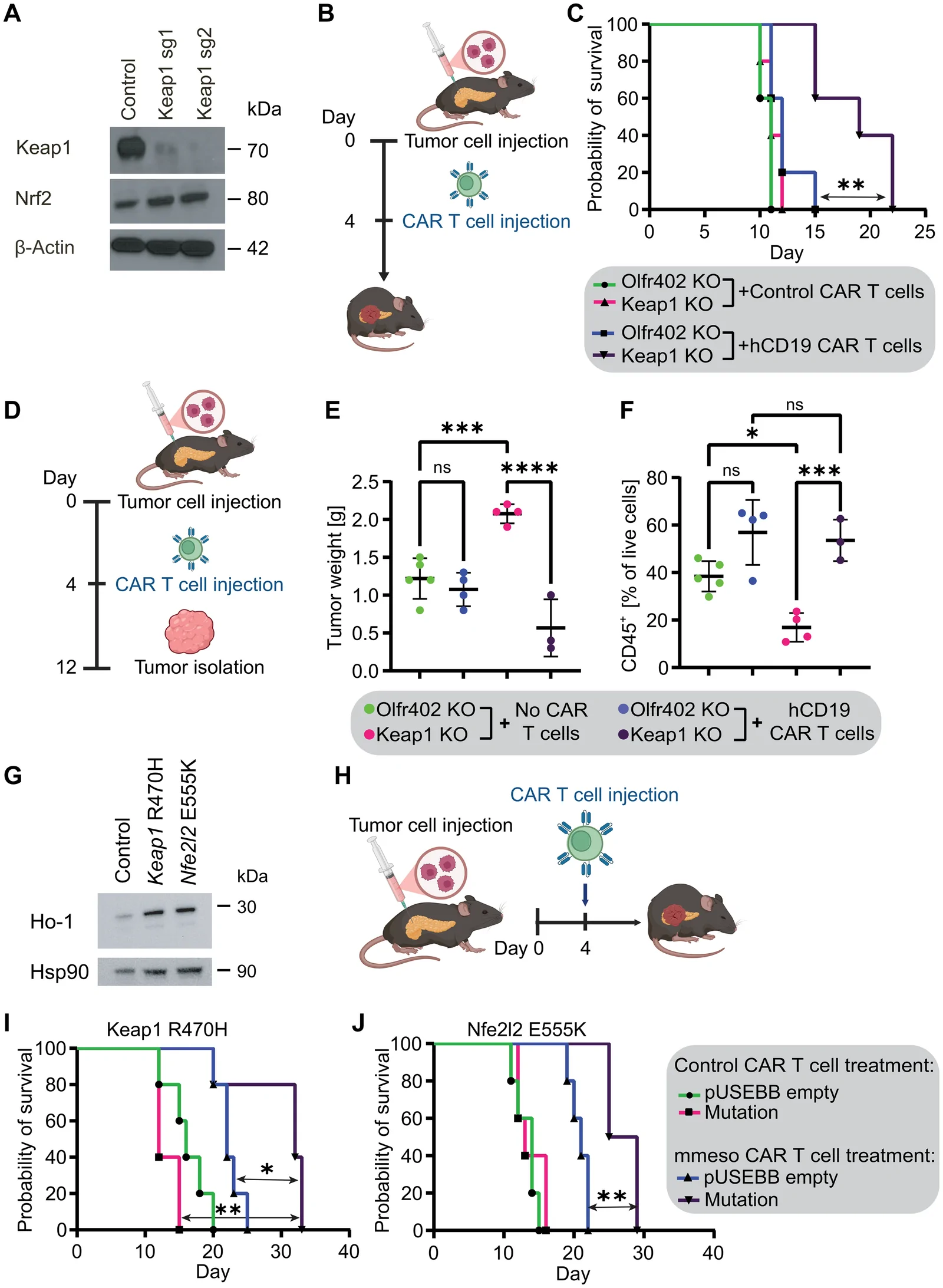
Nrf2 hyperactivation sensitizes to CAR T cell therapy in vivo. (**A**) Western blot confirming *Keap1* KO in bulk murine PDAC cells using two sgRNAs. (**B**) Orthotopic model: 6 × 10^5^ PDAC cells (control KO or *Keap1* KO) injected into C57BL/6 mice and treated with hCD19 or control (EGFRvIII) CAR T cells (5 × 10^6^; *n* = 5 per group) via intraperitoneal injection on day 4. Tumors were harvested when a humane endpoint was reached.* (**C**) Kaplan-Meier curve: *Keap1* KO tumors show extended survival following hCD19 CAR T treatment. Mantel-Cox test (*P* = 0.0086). (**D**) Orthotopic model: 6 × 10^5^ PDAC cells (control KO or *Keap1* KO) injected into C57BL/6 mice and treated with saline or hCD19 CAR T cells (2 × 10^6^; *n* = 3 to 5 per group) via intraperitoneal injection on day 4. Tumors harvested on day 12.* (**E**) Tumor weight at harvest. Error bars represent means ± SD. Ordinary one-way ANOVA. (**F**) Flow cytometry of CD45^+^ immune cells in control or *Keap1 KO* tumors ± hCD19 CAR T cells; percentage of immune cells of all live cells shown. Error bars represent means ± SD. Ordinary one-way ANOVA. (**G**) Western blot of Nrf2 effector Ho-1 in control, *Keap1* R470H, or *Nfe2l2* E555K PDAC cells. (**H**) Orthotopic model: 6 × 10^5^ PDAC cells injected into C57BL/6 mice and treated with mmeso or control CAR T cells [2 million (I) or 3 million (J)] via intraperitoneal injection on day 4. Tumors were harvested when a humane endpoint was reached.* (**I**) Kaplan-Meier curve: *Keap1* R470H mutation extends survival with mmeso CAR T treatment (*n* = 5 per group). Mantel-Cox test (*P* = 0.0277). (**J**) Kaplan-Meier curve: *Nfe2l2* E555K mutation extends survival with mmeso CAR T treatment (*n* = 4 to 5 per experimental group). Mantel-Cox test (*P* = 0.0065) [not significant (ns) > 0.05, *P ≤ 0.05, **P ≤ 0.01, ***P ≤ 0.001, and ****P ≤ 0.0001]. *Created in BioRender. Hemann, M. (2026) https://BioRender.com/uu8rpk2.

Next, we investigated whether *Keap1* loss in tumor cells causes any changes in the tumor microenvironment that may contribute to their increased susceptibility to CAR T cell therapy. We injected hCD19^+^ PDAC cells with control (*Olfr402*) KO or *Keap1* KO cells orthotopically into C57BL/6 mice and treated them with hCD19 CAR T cells or no CAR T cells (Fig. 7D). When we isolated and analyzed the tumors 12 days later, untreated *Keap1* KO tumors were significantly larger than untreated control KO tumors (Fig. 7E) and contained significantly fewer CD45^+^ immune cells (Fig. 7F) but an increased percentage of E2-Crimson^+^ PDAC cells (fig. S15A). This is in line with multiple studies highlighting *Keap1* KO tumors as “immune-cold” (*63*). Analysis of hCD19 surface antigen expression levels revealed no differences between untreated control KO and *Keap1* KO tumors (fig. S15B). The proportions of immune cells (with the exception of Ly6C^+^ Ly6G^−^ MDSCs) were largely similar between untreated control KO and *Keap1* KO tumors (fig. S15, C to L), rather the overall number of immune cells, including CD3^+^ T cells, was reduced in *Keap1* KO tumors (fig. S15, C and D). Conversely, hCD19 CAR T cell treatment increased the percentage of immune cells in both *Keap1* KO and control KO tumors compared to untreated tumors. A closer look at different immune subsets revealed no differences in T cell subsets, B cells, NK cells, and myeloid cell populations, between hCD19 CAR T cell–treated control KO and *Keap1* KO tumors, indicating similar levels of immune infiltration in response to CAR T cell therapy. Together, untreated *Keap1* KO PDAC tumors are immune-cold compared to control KO tumors but, nevertheless, remain susceptible to CAR T cell therapy.

To define the tumor cell–intrinsic changes caused by *Keap1* KO in PDAC cells that may underlie their sensitivity to CAR T cell therapy, we performed bulk RNA transcriptome analysis on PDAC cells isolated from untreated *Keap1* KO and control KO tumors. Following alignment of sequencing reads (fig. S16A), we performed differential gene expression analysis on control KO tumor cells compared to *Keap1* KO tumor cells (fig. S16B). GSEA on these differentially expressed genes revealed a strong depletion in inflammatory response signaling, such as IFN-γ, IFN-α, and tumor necrosis factor–α (TNF-α) signaling, in *Keap1* KO PDAC cells (fig. S16C), similar to that previously observed with *Slc33a1* loss (fig. S14C). Furthermore, *Keap1* loss led to an enrichment in ROS pathway signaling, consistent with an increased tolerance of oxidative stress caused by Nrf2 hyperactivation.

Given that both *Slc33a1* KO and *Keap1* KO can sensitize PDAC cells to CAR T cell therapy, we compared their bulk transcriptome data (figs. S14 and S16, A to C). GSEA confirmed enrichment of the UPR in *Slc33a1* KO cells and enrichment in ROS pathway signaling in *Keap1* KO cells, respectively, as some of the top divergent pathways between the two gene KO cells (fig. S16E). Thus, *Keap1* loss and *Slc33a1* loss appear to impose different intrinsic stresses in PDAC cells that converge on increased sensitivity to CAR T cell therapy.

### Patient-derived mutations in *KEAP1* and *NFE2L2* sensitize to CAR T cell therapy

Loss-of-function mutations in *KEAP1* and gain-of-function mutations in *NFE2L2* are observed in *KRAS*-driven malignancies, including in LUAD and PDAC (*48*), prompting us to hypothesize that patient-derived mutations could also sensitize tumors to CAR T cell therapy. According to the AACR GENIE database, 0.9% of patients with PDAC carry mutations in KEAP1, and 0.6% carry mutations in NFE2L2. While the rate of mutations in these two genes in patients with PDAC is low, NRF2 is frequently hyperactivated, with strong cytoplasmic NRF2 expression reported in ∼84% of PDAC tumors and nuclear NRF2 staining detected in ∼70% of cases (*64*, *65*). To test this hypothesis, we used base editing to engineer the murine ortholog of the human tumor–derived *Keap1* R470H mutation or *Nfe2l2* E563K (E555K in mice) mutation in WT PDAC cells. Mutations at *KEAP1* R470 are observed in patients with PDAC and have been previously shown to enhance NRF2 stability and target gene activation (*66*–*68*). In line with this, the *Keap1* R470H loss-of-function mutation leads to activation of Nrf2 as judged by increased protein levels of its downstream target heme oxygenase [Ho-1; (*69*)] compared to WT PDAC cells (Fig. 7G). The *Nfe2l2* mutation E555K also led to a similar increase in Ho-1 protein levels, suggesting that it may act as a gain-of-function mutation. Next, we orthotopically transplanted WT, *Keap1^R470H/R470H^* PDAC cells followed by treatment with mmeso or control CAR T cells (Fig. 7H). Before their injection in vivo, we confirmed that mmeso CAR T cells were efficient at killing WT PDAC cells in vitro (fig. S17A) while producing high levels of IFN-γ (fig. S17B). Consistent with our hypothesis, we observed significant survival extension in mice harboring *Keap1^R470H/R470H^* PDAC tumors compared to nonmutant PDAC tumors treated with mmeso CAR T cells (*P* = 0.0277; Fig. 7I). Similarly, mice with PDAC tumors containing the *Nfe2l2^E555K^* mutation showed a significant longer survival compared to mice with nonmutant PDAC tumors treated with mmeso CAR T cells (*P* = 0.0065; Fig. 7J). These results show that human tumor–derived *Keap1* and *Nfe2l2* mutations can sensitize tumors to CAR T cell therapy.

## DISCUSSION

CAR T cell therapies have shown limited success in PDAC clinical trials for reasons that remain poorly understood. CAR T cell failure alone is not sufficient to explain the resistance of PDAC to this therapy, suggesting that tumor-intrinsic factors may also be important. Efforts to discover tumor-intrinsic mechanisms of resistance have been hampered by the lack of faithful preclinical model systems that are amenable to high-throughput genetic screens. Particularly challenging are large-scale CRISPR-Cas9 KO screens in PDAC models in vivo due to limited orthotopic engraftment rates that do not achieve appropriate sgRNA representation required for screening genome-scale CRISPR libraries. Hence, in vivo screens in PDAC models published to date often used small libraries, have rarely been performed in orthotopic allografts, and mainly focused on the discovery of genes essential for PDAC growth (*70*–*72*). Multiple in vivo screens have sought to identify genes mediating general immune evasion in PDAC and have highlighted a role for antigen presentation, IFN-γ pathway signaling, and lipid metabolism (*45*, *73*). Screening for CAR T cell response modulators in other tumor models has revealed roles for antigen loss (*10*–*12*), impaired death receptor signaling in tumor cells (*13*), and alterations in the IFN-γ pathway (*14*–*16*, *25*). However, to date, no in vivo screens for mediators of CAR T cell response have been performed in solid tumors, including PDAC models.

Here, we established a fully immunocompetent, orthotopic PDAC model that can be used for multiplexed in vivo CRISPR screens in tumor cells to discover and validate mediators of resistance to CAR T cell therapy. We used a modular CRISPR library that is divided into individual, nonredundant pools, enabling us to perform iterative screening of more than one-third of the protein-coding genome and all KEGG pathway–associated genes. Through this large-scale screening approach, metabolic genes, particularly *Slc33a1*, emerged as critical modulators of CAR T cell therapy response in PDAC cells.

Using scRNA transcriptome analysis as an orthogonal approach, we found that PDAC cells resistant to CAR T cell therapy display a distinct metabolic cell state, characterized by reduced *Nrf2* pathway activation, glutathione metabolism, oxidative phosphorylation, and ROS signaling. Consistent with this observation, we demonstrated that PDAC cells with high Nrf2 pathway activity, a marker of the tolerance of oxidative stress, are hypersensitive to, and eliminated by, CAR T cell therapy in vivo (Fig. 8). Under homeostatic conditions, the antioxidant transcription factor Nrf2 is kept inactive by the oxidative stress sensor Keap1 and is only released upon stress conditions (*74*–*76*). Thus, cells with loss of *Keap1* function are known to experience Nrf2 hyperactivation, resulting in a redox imbalance that can promote tissue injury and cancer progression (*77*, *78*). Our experiments revealed that activation of the Keap1/Nrf2 pathway, either via endogenous activation or genetic ablation of *Keap1*, was sufficient to sensitize PDAC cells to CAR T cell therapy (Fig. 8). In addition, we show that patient-relevant mutations in *Keap1* and *Nfe2l2* can similarly sensitize PDAC tumors to CAR T cell treatment in vivo.

**Fig. 8.**
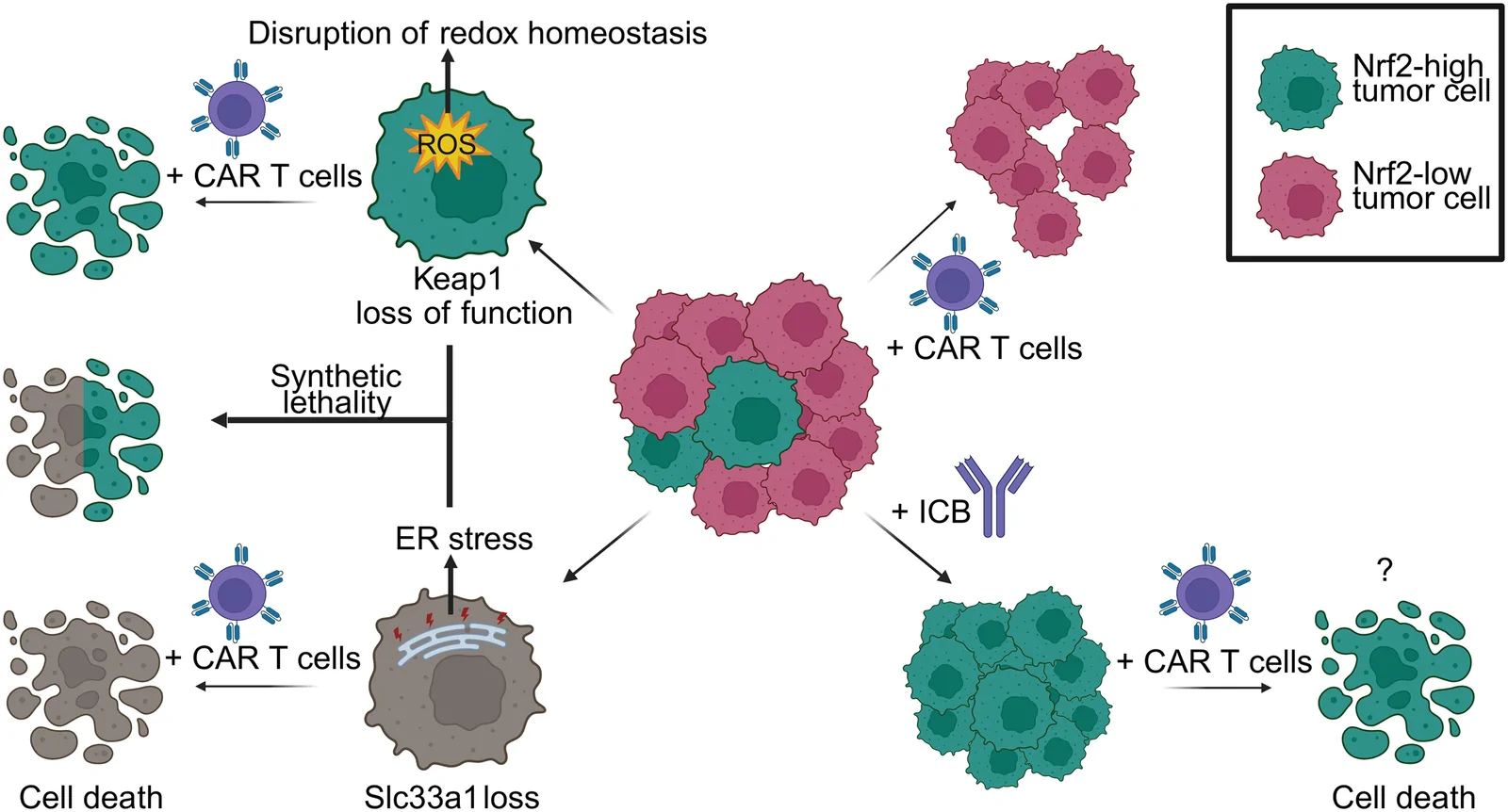
Model: Tumor cell–intrinsic stress states drive sensitivity to CAR T cell therapy. PDAC tumors contain Nrf2-high and Nrf2-low tumor cells. CAR T cell therapy effectively eliminates preexisting Nrf2-high tumor cell populations. In addition, tumor cell–intrinsic stress states, such as ER stress caused by *Slc33a1* loss or reductive stress caused by *Keap1* loss of function, sensitize to CAR T cell therapy. Combined inactivation of both *Slc33a1* and *Keap1* genes leads to tumor cell death driven by disruptions of redox homeostasis and proteostasis. Furthermore, the clinical observation that *Keap1* KO cells are particularly resistant to ICB therapy opens the possibility of the selection of Nrf2-hyperactivated tumor cells by ICB, which may then in turn be sensitized to CAR T cell therapy. Created in BioRender. Hemann, M. (2026) https://BioRender.com/rgvrw4b.

Our findings contrast with observations from ICB studies, where *Keap1* loss-of-function mutations have been associated with therapy resistance in preclinical models and patients with LUAD (*79*, *80*). *Keap1*-mutant tumors are characterized by an immunosuppressive, cold tumor environment, a finding that we recapitulated in our experiments. Although both ICB and CAR T cell therapy fall under the immunotherapy umbrella, the two therapeutic modalities engage fundamentally different mechanisms. ICB relies on antibodies to enhance the endogenous immune response to antigens presented by the major histocompatibility complex I (MHC-I) on tumor cells, a process requiring both DCs and T cells (*81*). In contrast, CAR T cell therapy does not require antigen presentation by MHC-I nor the help of additional endogenous immune cell populations to mount an immune response. Hence, our results may be reflective of mechanisms that are unique to adoptive cell-based immunotherapy and highlight the power of our modular in vivo screening platform to uncover such context-dependent tumor sensitivities. Furthermore, given that *Keap1* KO cells are particularly resistant to ICB therapy, this raises the intriguing possibility that ICB may select for a Nrf2-hyperactivated state that is responsive to subsequent CAR T cell therapy (Fig. 8).

The acetyl-CoA transporter Slc33a1, identified as a determinant of resistance in our CRISPR-Cas9 screens, has recently been shown as a key mediator of tolerance to *Nrf2* hyperactivation states in *Keap1* mutant LUAD (*54*). Our data reinforce the important functional link between Slc33a1 and the Keap1/Nrf2 pathway. Combined loss of *Slc33a1* and *Keap1* in our PDAC cells resulted in synthetic lethality (Fig. 8). We further showed that loss of *Slc33a1* alone sensitizes PDAC cells to CAR T cell therapy (Fig. 8). Notably, *Slc33a1* loss in PDAC cells did not alter the tumor microenvironment but caused tumor cell–intrinsic ER stress and the activation of the UPR, in agreement with prior studies in LUAD and pancreatic acinar cells (*54*, *59*). We show that this disruption of proteostasis is distinct from the disruption of redox homeostasis caused by *Keap1* loss, suggesting that disruption of distinct stress adaptation pathways can independently mediate PDAC susceptibility to CAR T cell therapy.

Recently, the concept that tumor cell states can influence therapy response in PDAC has gained notable traction. For example, PDAC cells in the classical epithelial cell state have been shown to drive resistance to *KRAS* inhibitors, exhibiting enhanced sensitivity to cytotoxic T cell killing (*23*, *24*). However, to date, no tumor cell states have been found that influence CAR T cell therapy response. Our study decisively shows that tumor-intrinsic metabolic stress states can profoundly sensitize cancer cells to CAR T cell therapy. These cell states can be defined by oxidative the disruption of redox homeostasis, characterized by high activity of the Nrf2 antioxidant response pathway, or proteostasis, e.g., through *Slc33a1* loss.

Our data suggest that CAR T cell–sensitive cell states can arise through genetic and nongenetic mechanisms, including *Keap1* mutations and endogenous tumor evolution, respectively. Currently, no tumor-intrinsic mutations, except those present in tumor antigens, are known to modulate the response to CAR T cell therapy, and we lack any tumor biomarkers that predict CAR T cell response. Here, we show that perturbations driving PDAC development, evolution, and therapeutic resistance may also lead to a therapeutically exploitable collateral sensitivity to CAR T cell therapy. Future clinical studies are needed to identify if patients with PDAC harboring tumor-intrinsic alterations in the Keap1/Nrf2 antioxidant pathway are sensitive to CAR T cell therapy. Nevertheless, our findings open the possibility of therapeutic synergy through drugs targeting the Nrf2 antioxidant pathway in combination with CAR T cell therapies to increase their efficacy in PDAC. Alternatively, therapies that drive the phenotypic selection for *Keap1*-mutant tumor cell states could be combined in sequence with CAR T cells that target this nascent vulnerability.

## MATERIALS AND METHODS

### Study design

This study aimed to identify tumor-intrinsic mechanisms of resistance to CAR T cell therapy in PDAC. We used syngeneic C57BL/6 and NSG mouse models, murine PDAC cell lines, and primary murine T cells. Orthotopic PDAC transplantation followed by adoptive CAR T cell transfer in vivo was complemented by in vitro cytotoxicity assays and cytokine release assays. We performed iterative pooled CRISPR-Cas9 screens in cancer cells for genetic determinants of resistance. Furthermore, we used bulk and single-cell transcriptomics to characterize treatment-associated transcriptional changes.

Sample sizes were based on prior experience with these models to ensure reproducibility, and predefined humane endpoints guided stopping rules. Animals showing distress unrelated to tumor burden were excluded. Outliers in CRISPR screen counts and tumor measurements were defined prospectively and excluded as noted. For CAR T cell treatments, tumor-bearing mice were randomly assigned to treatment groups. Replicate numbers are reported in figure legends, and key findings were confirmed under multiple conditions.

### PDAC cell transplantation

PDAC cells were orthotopically injected into the pancreas as previously described (*82*). Briefly, PDAC cells were resuspended in Hanks’ balanced salt solution, and 600,000 cells in 100 μl were orthotopically injected into the tail of the pancreas using a 26G1/2 needle. Successful injection was confirmed by visible bubble formation in the pancreatic parenchyma. Four days later, mice were intraperitoneally injected with CAR T cells suspended in saline on the opposing abdominal site. For experiments in which tumor growth was monitored, mice were imaged every other day using ultrasound. As indicated, tumors were either isolated at defined time points after initial injection or mice were closely monitored for signs of disease and distress and sacrificed upon the appearance of morbidity, in accordance with Committee on Animal Care (CAC) and Division of Comparative Medicine (DCM) policy at the Massachusetts Institute of Technology (MIT).

### Ethics statement

The research conducted in this study was conducted ethically and complies with all relevant guidelines and regulations. Animal studies were performed under strict compliance with the CAC at MIT (study approval number 2402000631).

### Statistical analysis

All statistical analyses were conducted using GraphPad Prism 10 [GraphPad Software Inc. (RRID:SCR_002798)]. The specific statistical tests performed for each analysis are outlined in the figure legends. Differences were considered statistically significant for *P* values ≤ 0.05. Significant differences are indicated as *P ≤ 0.05, **P ≤ 0.01, ***P ≤ 0.001, and ****P ≤ 0.0001.

## Supplementary Materials

### The PDF file includes:

Supplementary Materials and Methods Tables S5 and S6 Figs. S1 to S17 Legends for tables S1 to S4 References
