# Supplementary material for: Tumor cell–intrinsic stress states drive sensitivity to CAR T cell therapy in pancreatic cancer
Source: Sci Adv. 2026 Oct 2;12(40):eaeh8973. doi: 10.1126/sciadv.aeh8973 (PMC13641470; doi:10.1126/sciadv.aeh8973)
Supplement: Supplementary file 1 [file sciadv.aeh8973_sm.pdf]

Science Advances
AAAS

Supplementary Materials for

Tumor cell–intrinsic stress states drive sensitivity to CAR T cell therapy in pancreatic cancer

Julia Fröse *et al.*

Corresponding author: Michael T. Hemann, hemann@mit.edu

Sci. Adv. **12**, eaeh8973 (2026)
DOI: 10.1126/sciadv.aeh8973

The PDF file includes:

Supplementary Materials and Methods
Tables S5 and S6
Figs. S1 to S17
Legends for tables S1 to S4
References

Other Supplementary Material for this manuscript includes the following:

Tables S1 to S4

Supplemental Materials and Methods

Cell culture and cell lines. The cell lines utilized in this study comprised murine PDAC cells (kindly provided by the Vander Heiden Laboratory), human embryonic kidney 293T cells (HEK293T; RRID:CVCL_0063) and Platinum-E (Plat-E; RRID:CVCL_B488) retroviral packaging cells. HEK293T cells, acquired from the American Type Culture Collection (ATCC), were sustained in DMEM with L-glutamine and sodium pyruvate (Corning, 10-013-CM), supplemented with 10% FBS. Murine PDAC cells were grown in DMEM with L-glutamine and sodium pyruvate (Corning, 10-013-CM), supplemented with 10% FBS. Plat-E cells were acquired from Cell Biolabs, Inc. and cultured in DMEM with 10% fetal bovine serum (FBS), 1 μg/mL puromycin, 10 μg/mL blasticidin, penicillin and streptomycin. Routine mycoplasma contamination checks were conducted for all cell lines.

Primary murine T cells were procured from mouse spleens and cultured on plates coated with activating antibodies, as described in the CAR T cell production methods. The T cell medium consisted of RPMI with L-glutamine (Corning, 10-040-CM), supplemented with 10% FBS, recombinant human IL-2 (rhIL-2, final concentration of 20 ng/mL; Peprotech, Cat# 200-02-1mg), and 2-mercaptoethanol to a final concentration of 0.05 mM (Gibco, 21985023).

Viral supernatant production. Lentiviral supernatant was produced using standard methods. Briefly, HEK293T cells were transfected with lentiviral transfer plasmid and packaging vector (psPAX2 and pMD2.G) using Mirus TransIT-LT1 (Mirus, MIR2305) as indicated by the manufacturer. For retroviral supernatant production, Plat-E cells were switched to DMEM + 10% FBS without antibiotics and transfected with retroviral transfer plasmid using Mirus TransIT-LT1 (Mirus, MIR2305) as indicated by the manufacturer. Viral supernatant was collected 48 hours and

72 hours after transfection, passed through a 0.45 μm filter, and stored at 4 °C for a maximum of one day.

CAR T cell production. Murine $CD8^+$ T cells were isolated from the spleens of 14-week-old male or female C57BL/6 mice (Jackson Laboratory) using Miltenyi Biotec CD8a (Ly-2) MicroBeads for mouse (positive selection kit; Miltenyi, 130-117-044) and LS columns (Miltenyi, Cat# 130-042-401) as per the manufacturer's instructions. The isolated T cells were cultured at $1x10^6$ cells/mL on 6-well plates coated with anti-murine CD3e and anti-murine CD28 activating antibodies (Bio X-Cell, BE0001-1 and BE0015-1) in T cell media.

After 24 hours, activated T cells were collected, counted, and resuspended at $0.5x10^6$ in a 1:1 mixture of fresh T cell media with viral supernatant supplemented with protamine sulfate to a final concentration of 10 μg/mL (MS Biomedicals, ICN19472910). The cells were spin-infected at 1000xg for 1.5 hours at 37 °C on new antibody-coated plates. The next day, T cells were again collected, counted, and resuspended at $1x10^6$ cells/mL in fresh T cell media, re-plated on new antibody-coated plates. Lastly, 24 hours later, T cells were collected, counted, and the percentage of CAR^+ (GFP^+) T cells determined by flow cytometry. The desired number of CAR T cells was then prepared for injection by resuspension in saline and injected intraperitoneally. Alternatively, T cells were resuspended at this step in T cell media and plated for *in vitro* cytotoxicity assays.

***In vitro* cytotoxicity assay.** Briefly, target cells were counted and co-cultured with or without CAR T cells at specified Effector-to-Target (E:T) ratios, accounting for the CAR T transduction efficiency, in T cell media. After approximately 24 hours, the cell suspension was subjected to flow cytometry analysis to evaluate live/dead status (via DAPI stain), %$hCD19^+$ cells, and %$CD8^+$ cells. Flow cytometry was also employed to determine the densities of each cell type (CAR T, target cell, non-transduced T cell). The resulting target cell densities in CAR T-containing wells

were normalized to those in control wells, seeded with the same number of target cells but with control CAR T or non-transduced T cells. All flow cytometry experiments were conducted with a minimum of 10,000 live cells (via DAPI exclusion) and subsequent data analysis.

Interferon gamma release ELISA assay. The enzyme-linked immunosorbent assay (ELISA) followed standard procedures. Briefly, supernatants from *in vitro* CAR T cytotoxicity assays were collected and centrifuged to eliminate any contaminating cells. The quantification of IFNγ released by CAR T cells in the supernatant was performed using the DuoSet ELISA kit for mouse IFNγ (R&D systems, DY485). Nunc MaxiSorp flat-bottom plates (Thermo Fisher Scientific, 44-2404-21) were employed for the assay, conducted on a Tecan Infinite 200 Pro machine according to the manufacturer's instructions. To maintain the assay within the linear range of the kit, the supernatant was initially diluted at 1:10 in reagent diluent. Subsequently, a minimum of six serial 4-fold dilutions were executed. For each plate, at least one standard curve was generated, and the entire experiment included at least two standard curves, constructed using standard solutions supplied by the manufacturer. The substrate solution used was 1-StepTM Ultra TMB-ELISA (Thermo Fisher Scientific, 34028), and the stop solution employed was 2N sulfuric acid (VWR, BDH7500-1). Bovine serum albumin (BSA; Sigma, A8022-500G) was prepared as a sterile-filtered 5% stock in PBS (Corning, 21-031-CV).

Fluorescence-activated cell sorting and analysis. The BD LSRFortessa Cell Analyzer (RRID:SCR_018655) in tube or plate reader format, with BD FACSDiva Software (RRID:SCR_001456) v9.0 was used for data collection. All flow cytometry experiments were conducted with a minimum of 10,000 live cells (via DAPI exclusion). Downstream analysis was performed using FlowJo 10.9.0. The BD FACSAria II Cell Sorter (RRID:SCR_018934) was used for cell sorting.

Western blotting. Cells were lysed with RIPA buffer (Boston BioProducts, BP-115) supplemented with 1X protease inhibitor mix (cOmplete EDTA-free, 11873580001, Roche). Protein concentration of cell lysates was determined using Pierce BCA Protein Assay (ThermoFisher Scientific, 23225). Total protein (25-40 μg) was separated on 4-12% Bis-Tris gradient SDS-PAGE gels (Life Technologies) and then transferred to PVDF membranes (IPVH00010, EMD Millipore) for blotting.

Base editing. An sgRNA targeting R470 in *Keap1* or E555 in *Nfe2l2* was cloned into the pUSEBB backbone *(83)*. Lentivirus was produced and WT PDAC cells were transduced and sorted for sgRNA expression (BFP+). Next, cells were transduced with a cytosine base editing (CBE) reporter (mScarlet+) *(83)*, sorted and electroporated with *in vitro* transcribed mRNA encoding CBE6-NG. Successful base editing at the CBE reporter leads to GFP expression. After 72 hours of recovery, cells were sorted for GFP to enrich for base-edited PDAC cells. Next, cells with *Keap1* R470H mutation were single cell-cloned and successful editing was confirmed via Sanger sequencing. A single cell clone with homozygous *Keap1* R470H mutation was used for further experiments. For cells with the Nfe2l2 E555K mutation, bulk editing efficiency was confirmed via Sanger sequencing (82% correct editing) and the bulk population was used for further experiments.

Animal housing and maintenance. All mouse experiments were conducted under Institutional Animal Care and Use Committee (IACUC)-approved animal protocols (protocol number 2402000631) at the Massachusetts Institute of Technology (MIT). The mouse strains utilized in this study included C57BL/6 (The Jackson Laboratories; RRID:IMSR_JAX:000664) and NOD/SCID/IL-2Rg$^{-/-}$ (NSG; The Jackson Laboratories; RRID:IMSR_JAX:005557), as indicated in the figure legends. All experimental mice used were 10-16 weeks old. Mice were housed under social conditions (two to five mice per cage) on a 12-hour dark/12-hour light cycle, ambient

temperature 21 °C ± 1 °C, and humidity 50% ± 10%. All animals were housed in the pathogen-free animal facility of the MIT Koch Institute, in accordance with the animal care standards of the institutions. Food and water were provided *ad libitum*. All animal research at MIT is conducted under humane conditions with utmost regard for animal welfare. The animal care facility staff is headed by a chief veterinarian and includes a veterinary assistant, animal care technicians, and administrative support. MIT adheres to institutional standards for the humane use and care of animals, which have been established to assure compliance with all applicable federal and state regulations for the purchase, transportation, housing, and research use of animals.

Limiting GFP-dilution assay. 600,000 PDAC cells were orthotopically transplanted into C57BL/6 mice. Five experimental groups of PDAC cells were tested containing diminishing amounts of GFP+ PDAC cells in an RFP+ PDAC cell population: (1) 50% RFP+ and 50% GFP+, (2) 99%RFP+ with 1% GFP+, (3) 99.9% RFP+ with 0.1% GFP+, (4) 99.95% RFP+ with 0.05% GFP+, (5) 99.975% RFP+ with 0.025% GFP+ PDAC cells. Ten days after transplantation, PDAC tumors were isolated and analyzed by flow cytometry for GFP+ PDAC cells. The recovered percentage of GFP+ cells were then compared to the initial percentage of GFP+ PDAC cells prior to orthotopic transplantation.

Competition assay. For *in vitro* competition assays, PDAC cells with or without hCD19 antigen were mixed at equal ratios and seeded at $1x10^4$ cells per well into a 48-well plate. Next, freshly prepared control (EGFRvIII) or hCD19 CAR T cells were added at an E:T ratio of 10:1 to the PDAC cells. After approximately 24 hours, the cell suspensions were subjected to flow cytometry analysis to evaluate live/dead status (via DAPI stain), %hCD19$^+$ PDAC cells (GFP+) and %hCD19- PDAC cells (E2-Crimson+). The resulting target cell densities in CAR T-containing

wells were normalized to those in control wells, seeded with the same number of target cells but with control CAR T or non-transduced T cells.

For *in vivo* competition assays, PDAC cells with or without hCD19 antigen were mixed at equal ratios and 600,000 total PDAC cells were orthotopically transplanted into C57BL/6 mice. Four days later, mice were injected with 2.5 million control (EGFRvIII) or hCD19 CAR T cells, or with saline as a negative control. Ten days after transplantation, PDAC tumors were isolated and analyzed by flow cytometry. Antigen-negative PDAC cells were identified by E2-Crimson expression and antigen-positive PDAC cells were identified by GFP expression. The ratios of GFP+ to E2-Crimson+ cells were compared between the three experimental groups.

Pooled sgRNA screening. The modular SKY CRISPR library which is divided into 48 individual, non-redundant pools that can be individually screened or combined into larger libraries was used. In this library, Kyoto Encyclopedia of Genes and Genomes (KEGG (RRID:SCR_012773)) protein-coding genes are distributed into pools by pathway ranking, leading to the first 15 pools of the library covering all KEGG pathways. After the first 15 pools, the gene distribution is random. Thus, we elected to screen the first 16 pools of this library in pairs of two to cover all major signaling pathways. To preserve library complexity, a minimum of 1000-fold coverage of the sgRNA library was maintained at each *in vitro* step before the screen, and at a minimum of 150-fold coverage was maintained in all screens completed. Cloned and sequenced plasmid pools, and viral supernatant were generated by the Broad Institute's GPP. For screens, Cas9+ cells were thawed, recovered, and expanded for 5 days to ensure robust growth, and then tested for cutting efficiency to ensure high rates of editing efficiency *(84)*. In brief, Cas9+ PDAC cells were transduced with the pXPR-047 plasmid that delivers both tdTomato and a sgRNA targeting tdTomato. Thus, active Cas9-expressing lines will result in a reduction in tdTomato over time.

After cutting assays were completed, cells were expanded over three additional days and infected with sub-pools. For each of the 16 sub-pools, $6x10^6$ cells were spin-infected with predetermined amounts of viral supernatant such that 30-50% of all cells were infected (expressed E2-Crimson, and survived puromycin selection; MOI«1). Forty-eight hours later, 2 million transduced PDAC cells were started on puromycin selection (5 µg/ml, Gibco, A1113803). Cells were selected over seven days and allowed to recover for two days. On the day of injection into mice, the appropriate number of infected, selected, and recovered cells were combined so that each screen contained two pools. For each screen, PDAC cells injected into the pancreas of C57BL/6 mice as described under PDAC cell transplantation section of this manuscript. On the same day as the injection, input samples were collected. Four days later, 2.5 million control (EGFRvIII) or hCD19 CAR T cells were adoptively transplanted into mice via intraperitoneal injection. Mice were sacrificed upon relapse and their tumors were isolated and dissociated into single cell suspensions. For this purpose, tumors were minced with a scalpel and incubated for 30 min in a HBSS-based digestion buffer containing 10 mM HEPES (Invitrogen, #15630080), 12.5U Collagenase IV (Millipore Sigma, C2674), and 200U DNaseI (NEB, #M0303) at 37°C. Next, tumors were further dissociated using a gentleMACS tissue dissociator (Miltenyi Biotec). Enzyme activity was quenched with FBS and the samples were filtered through sequential filtering steps to obtain single cell solutions. Finally, gDNA from all cells was isolated using the Machery Nagel L Midi NucleoSpin Blood Kit (Clontech, 740954.20). Modifications to the manufacturer's instructions were added as follows: in step1, cells were lysed in the kit's proteinase K containing lysis buffer for longer (overnight at 70 °C). The next morning, lysates were allowed to cool to room temperature, 4.1 µl of RNase A (20 mg/mL; Clontech, 740505) was added, and cells were incubated for 5 minutes at room temperature. The procedure then continued as indicated by the manufacturer. PCR inhibitors were

removed from the resulting gDNA (Zymo Research, D6030) and the concentration of the resulting gDNA was measured using the Qubit dsDNA HS assay kit (ThermoFisher, Q32854), and if necessary, diluted to 200 ng/μl with elution buffer. gDNA was then submitted for Illumina sequencing. Data were analyzed as specified in the Data Analysis section of this manuscript.

Single cell transcriptome profiling. C57BL/6 mice were orthotopically injected with $6x10^5$ hCD19+ PDAC cells, followed by intraperitoneal injection with murine CAR T cells targeting either hCD19 or a control epitope (human EGFRvIII). CAR T cell treated tumors were isolated after 10 days for control CAR T cell treated mice and after 15 days for hCD19 CAR T cell treated mice, at which stage all PDAC tumors had reached a similar weight. Tumors were dissociated into single cells and 10,000 live cells were submitted for transcriptional profiling. Single-cell expression libraries were prepared using the 10x Genomics Chromium v3 reagents. Data were analyzed as specified in the Data Analysis section of this manuscript.

Bulk tumor cell transcriptome profiling. C57BL/6 mice were orthotopically injected with $6x10^5$ hCD19+ PDAC cells with a control (*Olfr402*) KO, *Slc33a1* KO or *Keap1* KO. Tumors were isolated after 12 days, dissociated into single cells and sorted for E2-Crimson+ tumor cells. At least 100,000 tumor cells per sample were submitted in DNA/RNA shield solution (Zymo Research, #R1100) to Plasmidsaurus for bulk RNA transcriptome profiling. Data were analyzed by Plasmidsaurus as specified in the Data Analysis section of this manuscript.

***Nrf2* reporter experiment.** WT PDAC cells were transduced with a previously described *Nrf2* 8xARE-GFP reporter for which GFP expression indicates functional *Nrf2* transcriptional activity*(50)*. For *in vitro* validation of reporter function, *Nrf2* reporter PDAC cells were treated with tert-butylhydroquinone (tBHQ) and analyzed by flow cytometry for GFP expression 24 hours later. For in vivo experiments, C57BL/6 mice were orthotopically injected with $6x10^5$ *Nrf2*

reporter PDAC cells, followed by intraperitoneal injection with murine CAR T cells targeting either murine mesothelin or a control epitope (human EGFRvIII). CAR T cell treated tumors were isolated after 10 days, dissociated and PDAC cells (BFP+) were analyzed for GFP expression by flow cytometry.

Tumor flow cytometry profiling. C57BL/6 mice were orthotopically injected with $6x10^5$ hCD19+ PDAC cells with a control (*Olfr402*) KO, *Slc33a1* KO or *Keap1* KO, followed by intraperitoneal injection with murine CAR T cells targeting either hCD19 or a control epitope (human EGFRvIII). CAR T cell treated tumors were isolated after 12 days, and dissociated into single cells (as described above) for flow cytometric analysis. Samples were blocked with Fcr blocking reagent for mouse (Miltenyi Biotec 130-092-575) for 10 minutes at 4°C. Then samples were resuspended in antibody solution and incubated at 4°C for 1 hour. Following a wash step with PBS, samples were fixed using fresh 4% PFA for 20 minutes at room temperature. After another wash, samples were resuspended in PBS and data were collected within 24 hours using the BD LSRFortessa Cell Analyzer (RRID:SCR_018655) in tube or plate reader format, with BD FACSDiva Software (RRID:SCR_001456) v9.0. Downstream analysis was performed using FlowJo 10.9.0.

Tumor histology. PDAC tumors were fixed with 10% Formalin for 24 hours at 4°C and subsequently embedded in paraffin. Tumors were sectioned into 4 µm thick sections and stained with hematoxylin and eosin (H&E). Tumor sections were imaged using an Aperio Digital Pathology Slide scanner (Leica Biosystems) at 20x resolution and analyzed using the corresponding Aperio ImageScope software (RRID:SCR_020993).

Antibodies

Western blotting. anti-β-ACTIN (Cell Signaling Technology Cat# 4967, RRID:AB_330288), anti-Cas9 (Active Motif Cat# 61577, RRID:AB_2793684), anti-Slc33a1 (Sigma-Aldrich Cat# WH0009197M7, RRID:AB_2286124), anti-Nrf2 (Cell Signaling Technology Cat# 12721, RRID:AB_2715528) , anti-Keap1 (Cell Signaling Technology Cat# 8047, RRID:AB_10860776), anti-rabbit IgG HRP-linked antibody (Cell Signaling Technology Cat# 7074, RRID:AB_2099233), anti-mouse IgG HRP-linked antibody (Cell Signaling Technology Cat# 7076, RRID:AB_330924), anti-rat IgG HRP-linked antibody (Cell Signaling Technology Cat# 7077, RRID:AB_10694715), Rabbit anti-Armenian Hamster IgG H&L (HRP) (Abcam Cat# ab5745, RRID:AB_955407).

Flow cytometry. anti-human CD19-BV785 (BioLegend Cat# 302240, RRID:AB_2563442), anti-mouse Cd8a-PE/Cy7 (BioLegend Cat# 100722, RRID:AB_312761), anti-murine Cd45-PerCP-Cy5.5 (BioLegend Cat# 103132, RRID:AB_893340), anti-murine Cd45-BV785 (BioLegend Cat# 103149, RRID:AB_2564590), anti-murine Cd45-BV711(BioLegend Cat# 103147, RRID:AB_2564383), anti-murine Cd11b-APC-Cy7 (BioLegend Cat# 101226, RRID:AB_830642), anti-murine Cd11c-PerCP Cy5.5 (BioLegend Cat# 117328, RRID:AB_2129641), anti-murine Ly6C-BV605 (BioLegend Cat# 128035, RRID:AB_2562352), anti-murine Ly6G-FITC (Thermo Fisher Scientific Cat# 11-9668-82, RRID:AB_2572532), anti-murine F4/80-SparkUV387 (BioLegend Cat# 285504, RRID:AB_3717064), anti-murine Cd19-785 (BioLegend Cat# 115543, RRID:AB_11218994), anti-murine Nk1.1-PECy7 (Thermo Fisher Scientific Cat# 25-5941-81, RRID:AB_469664), anti-murine CD3-SparkUV387 (BioLegend Cat# 100284), anti-murine Cd8a-APC (BioLegend Cat# 100712, RRID:AB_312751), and anti-murine Cd4-PECy7 (BioLegend Cat# 100421, RRID:AB_312706).

Table S5: Plasmid constructs

**Plasmid**	**Producer**
psPAX2 (lentiviral packaging)	RRID:Addgene_12260
pMD2.G (VSV-g envelope)	RRID:Addgene_12259
lentiCas9-Blast	RRID:Addgene_52962
hCD19 CAR-P2A-GFP plasmid	*(14)*
EGFRvIII CAR-P2A-GFP plasmid	*(14)*
pMMLV hCD19(-ICD)	*(85)*
mmeso CAR-P2A-GFP plasmid	Sequence generously shared by the June lab and then cloned into the same plasmid backbone as other CAR plasmids used in this study.
pRDACrimson_170	*(14)*
pXPR-047	RRID:Addgene_107145 with tdTomato instead of eGFP;
Nrf2 8xARE-GFP reporter	*(50)*
pLEX307	RRID:Addgene_41392; backbone used for expression of Cas9 resistant *Slc33a1* for overexpression
pUSEBB	*(83)*
Cytosine base editor (CBE) reporter	RRID:Addgene_136895
SpCas9 CBE6-IVT template	RRID:Addgene_215834

CRISPR plasmids. For hit validation, the top 2 scoring sgRNAs for each gene were taken from the SKY library. All other sgRNAs were designed using the Broad Institute's sgRNA Designer tool (https://portals.broadinstitute.org/gppx/crispick/public). sgRNAs were cloned into pRDACrimson_170 or pUSEBB to generate KOs of indicated genes. The sgRNA sequence for *Keap1* R470H was kindly provided by Acosta, Johnson and Gould et al. *(86)* and is a murine equivalent of the human *KEAP1* R470H mutation.

Table S6: sgRNA sequences

sgRNA target	Forward sequence 5'->3'	Reverse sequence 5'->3'
Slc33a1 sg1	CACCGGAAAGGGTAACGATTCCCCT	AAACAGGGGAATCGTTACCCTTTCC
Slc33a1 sg2	CACCGATGTTATCCCGGGAAAACGT	AAACACGTTTTCCCGGGATAACATC
Keap1 sg1	CACCGCATGTACCAGATTGACAGCG	AAACCGCTGTCAATCTGGTACATGC
Keap1 sg2	CACCGCGCCTCATCTACACAGCGGG	AAACCCCGCTGTGTAGATGAGGCGC
hCD19 sg1	CACCGCCTCAGCCAGGACCTCACCA	AAACTGGTGAGGTCCTGGCTGAGGC
hCD19 sg2	CACCGCTAGGTCCGAAACATTCCAC	AAACGTGGAATGTTTCGGACCTAGC
hCD19 sg3	CACCGCTAGTGGTGAAGGTGGAAGA	AAACTCTTCCACCTTCACCACTAGC
hCD19 sg4	CACCGATGAAAAGCCAGATGGCCAG	AAACCTGGCCATCTGGCTTTTCATC
hCD19 sg5	CACCGGAGCCCCAAGCTGTATGTGT	AAACACACATACAGCTTGGGGCTCC
hCD19 sg6	CACCGAGTCAATGTGGAGGGCAGCG	AAACCGCTGCCCTCCACATTGACTC
Keap1 R470H	CACCGGAAGCGGTTGAGCACTGCCA	AAACTGGCAGTGCTCAACCGCTTCC
Nfel2l E555K	CACCGGACTTCAAGATACAAGGTGC	AAACGCACCTTGTATCTTGAAGTCC

Data Analysis

Primary CRISPR-Cas9 KO Screen

Count data was assigned to SKY library barcodes by the Broad Institute's Genomic Perturbation Platform (GPP) using PoolQ (version 3.3.1) from the output of 5 separate sequencing runs. The resulting 24 counts files were manually assembled using Tibco Spotfire version 11.4.1.16 and R version 4.4.1 *(87)* with tidyverse version 2.0.0 *(88)* and exported to 8 data matrices. Each matrix contains data for a pair of pools of the SKY library and contains sgRNA identifiers, the corresponding gene association for these identifiers and count data for input, EGFRvIII, and CD19 replicates. Interquartile ranges were calculated for each sample and 5 outliers were identified and

excluded from analysis, these were: pool 1+2, EGFRvIII replicate 2, pool 3+4 EGFRvIII replicate 5, pool 7+8 CD19 replicate 16, and pool 13+14 replicates CD19 14 and EGFRvIII 3. These count matrices were then used as input for differential comparison with MAGeCK test version 0.5.9.4 *(29)* with the parameters adjust-method fdr, gene-lfc-method median and norm-method control using a list of negative control guides that target intergenic or olfactory gene sequences. After testing, guide and gene-level data results for each experiment and pool were imported, processed and visualized in R using MAGeCKFlute version 2.8.0 *(89)* and tidyverse 2.2.0 *(88)*.

Gene and guide-level data were then aggregated into single data frames in order to visualize differential comparisons across the pools. The 16 pools of the SKY library screened in this study contains 29677 guides targeting barcodes. Of these 27705 target a single gene for a total of 7154 screened genes. Genes of interest and those exceeding different log fold change and FDR thresholds were then selected for validation screening.

Validation CRISPR-Cas9 KO Screen. A validation library was prepared consisting of 1960 sgRNAs. These include 376 non-targeting control sgRNAs and sgRNAs that target 328 genes, usually with 6 distinct guides. Differential analysis was performed for validation screen data with MAGeck test in the same way as for the primary screen. All validation screen samples were used in analysis. Metabolic genes were defined as those encoding enzymes or transporters participating in canonical metabolic pathways based on KEGG, Reactome, and GO. Genes involved in nutrient sensing, autophagy, and metabolic stress adaptation were classified as metabolism-adjacent.

Single cell transcriptome profiling. Sequencing data was aligned to the Mus_musculus.GRCm38v98.chr reference genome (with human CD19, Cas9 and EGFP added to it) and converted to fastq files using bcl2fastq (v2.20.0.422). Cell count matrices were generated

using cellranger (v.6.1.1). Matrices were analyzed by Seurat (v4.0.4) for R (v4.0.2). Digital gene expression matrices were filtered on the following criteria to exclude low-quality cells: number of genes detected in each cell **(=**nFeature_RNA >100, <8000), the total number of molecules detected within a cell **(=**nCount_RNA; top 10% removed) and percent mitochondrial reads (=percent.mito; <25%). The data were further filtered to exclude ambient RNA (SoupX) and doublets (DoubletFinder). Next, data from all tumors was combined into a shared data object and was normalized, scaled, and principal component analysis was performed on the scaled data. The standard deviation of principal components was quantified using an elbow plot, and input dimensions for SNN clustering (20) at which standard deviation = 2 was used. SNN clustering was performed to generate UMAP plots (k.param = 20, res = 0.8). Unique markers for each cluster were identified using FindAllMarkers function. Clusters were annotated using a mix of manual annotation based on well-known lineage genes, such as *Cd68* for macrophages, and the Bioconductor package SingleR. Tumor cells were identified by hCD19 and Cas9 expression and subsetted. Following normalization and scaling, principal component analysis was performed on the scaled data. SNN clustering was performed to generate UMAP plots (k.param = 10, res = 0.4). Differential abundance analysis was performed using MiloR to compare UMAP changes between control and hCD19 CAR T cell treated PDAC cells *(90)*. Differentially expressed genes between PDAC cell with control CAR T cell treatment and PDAC cells with hCD19 CAR T cell treatment were identified using the FindMarkers function. The top 100 differentially expressed genes were used for Gene Ontology (RRID:SCR_002811) analysis. A ranked list based on all differentially expressed genes was used for Gene Set Enrichment Analysis (RRID:SCR_003199). For the generation of a murine Nrf2-responsive gene signature, the human NRF2 WikiPathway gene set (species = "human", collection = "C2", sub collection = "CP:WIKIPATHWAYS", gene set=

"M39454") was used and murine orthologs to all genes were identified using the "orthologs" function of the R package "Disco" (v 0.6). The resulting gene list was filtered to only contain genes expressed in our PDAC cells. The murine Nrf2-responsive gene signature was then created of the resulting genes using the Seurat function "AddModuleScore" and displayed in a Feature Plot.

Bulk tumor cell transcriptome profiling. Quality of the fastq files was assessed using FastQC v0.12.1. Reads were then quality filtered using fastp v0.24.0 with poly-X tail trimming, 3' quality-based tail trimming, a minimum Phred quality score of 15, and a minimum length requirement of 50 bp. Quality-filtered reads were aligned to the reference genome using STAR aligner v2.7.11 with non-canonical splice junction removal and output of unmapped reads, followed by coordinate sorting using samtools v1.22.1. PCR and optical duplicates were removed using UMI-based deduplication with UMIcollapse v1.1.0. Alignment quality metrics, strand specificity, and read distribution across genomic features were assessed using RSeQC v5.0.4 and Qualimap v2.3, with results aggregated into a comprehensive quality control report using MultiQC v1.32. Gene-level expression quantification was performed using featureCounts (subread package v2.1.1) with strand-specific counting, multi-mapping read fractional assignment, exons and three prime UTR as the feature identifiers, and grouped by gene_id. Final gene counts were annotated with gene biotype and other metadata extracted from the reference GTF file. Sample-sample correlations for sample-sample heatmap and PCA were calculated on normalized counts (TMM, trimmed mean of M-values) using Pearson correlation. Differential expression was done with edgeR v4.0.16 using standard practice including filtering for low-expressed genes with edgeR::filterByExprwith default values. Functional enrichment was performed using gene set enrichment analysis with gseapy v0.12 using the MSigDB Hallmark gene set.

Supplemental figures

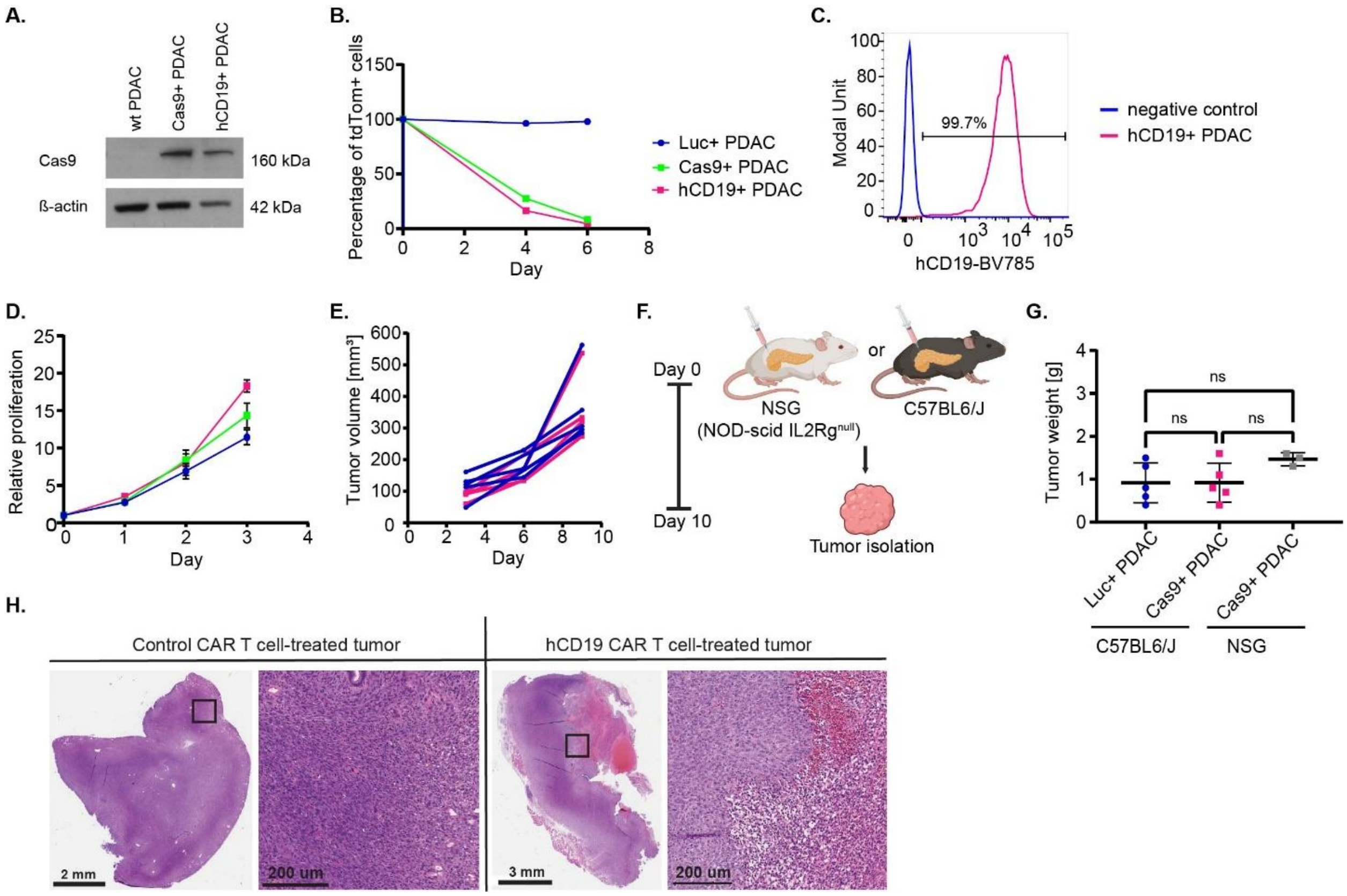

Figure S1: Development of a CRISPR-Cas9 knock-out screening-amenable PDAC model for studying CAR T cell therapy resistance. A) A western blot showing Cas9 protein expression in KPC PDAC cell lines. **B)** A graph showing Cas9 activity in PDAC cells *in vitro* as measured by disruption of tdTomato expression with an tdTomato-targeting sgRNA over a time period of 6 days. **C)** A flow cytometry plot showing hCD19 cell surface expression on hCD19+ PDAC cell compared to a negative control cell line. **D)** A graph showing *in vitro* proliferation of the Luc+ PDAC cell, Cas9+ PDAC cells, and hCD19+ PDAC cells. **E)** A graph showing *in vivo* proliferation of PDAC cells with and without Cas9 expression following orthotopic injection of 600,000 PDAC cells into C57BL/6 mice. **F)** Experimental schematic: Experimental schematic: C57BL/6 and NSG (Nod-scid IL2Rg^{null}) mice received an orthotopic injection of 600,000 murine PDAC cells with or without Cas9 and hCD19 expression. Tumors were isolated ten days later and their weight was measured. *Created in BioRender. Hemann, M. (2026) https://BioRender.com/uu8rpk2.* **G)** A graph showing the weight of PDAC tumors formed by Luc+ PDAC cells or hCD19+ PDAC cells in C57BL/6 mice and NSG mice. The tumor weights are not significantly different between mouse strains and PDAC cell lines. Statistical analysis with Ordinary One-Way ANOVA test (ns > 0.05). **H)** H&E-stained tumor sections from hCD19+ PDAC tumors treated with control CAR T cells targeting an irrelevant antigen (EGFRvIII) or treated with hCD19 CAR T cells. PDAC tumors treated with hCD19 CAR T cells show large necrotic areas and immune cell infiltration.

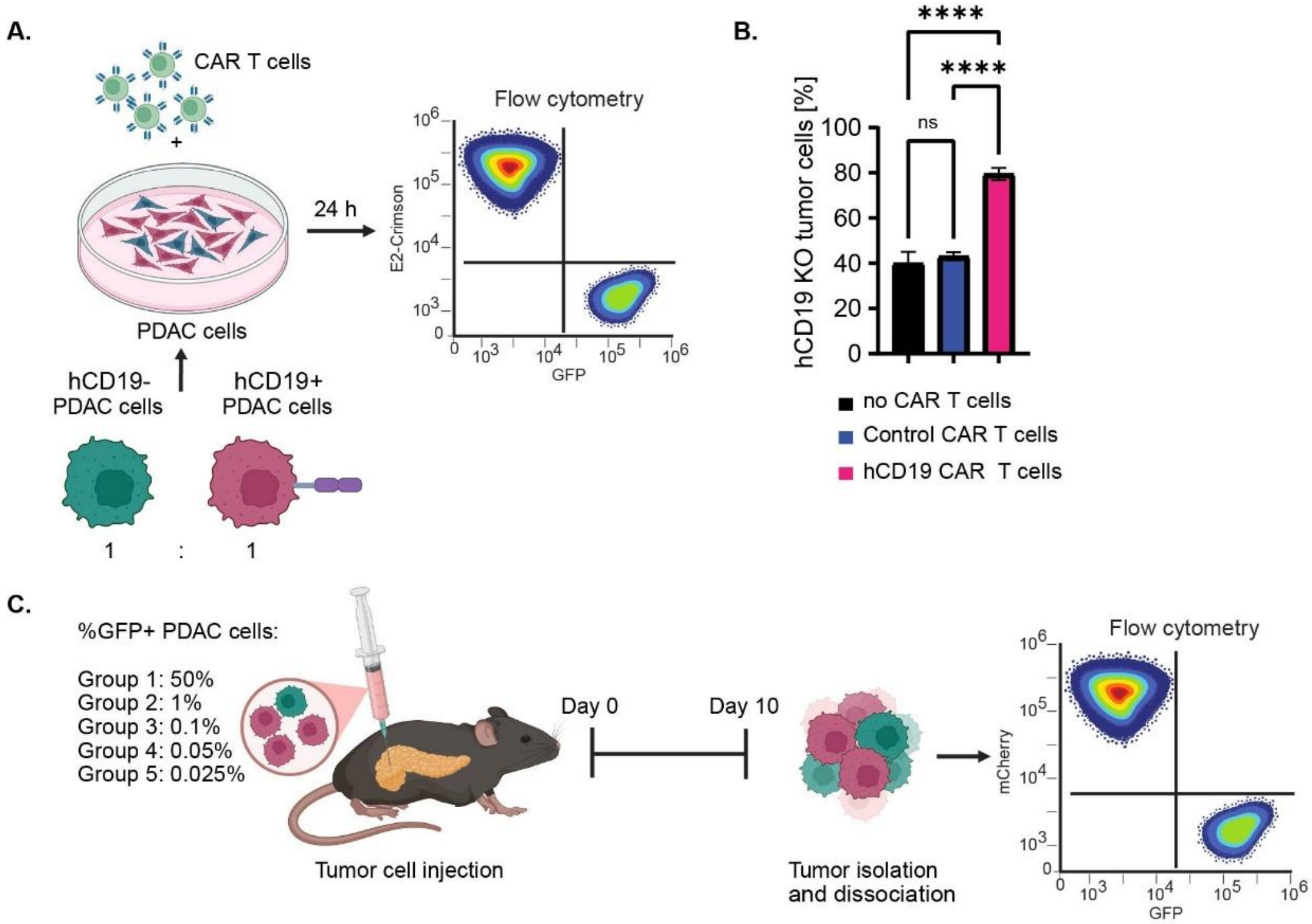

D.

		Expected input		True Input	Output
Group	Total no. of PDAC cells	%GFP	Maximum sgRNA library size	%GFP	Average %GFP
1	6.00E+05	50	2	51.5267%	52.0774%
2	6.00E+05	1	100	1.1974%	1.1089%
3	6.00E+05	0.1	1000	0.1058%	0.0947%
4	6.00E+05	0.05	2000	0.0453%	0.1283%
5	6.00E+05	0.025	4000	0.0268%	0.0299%

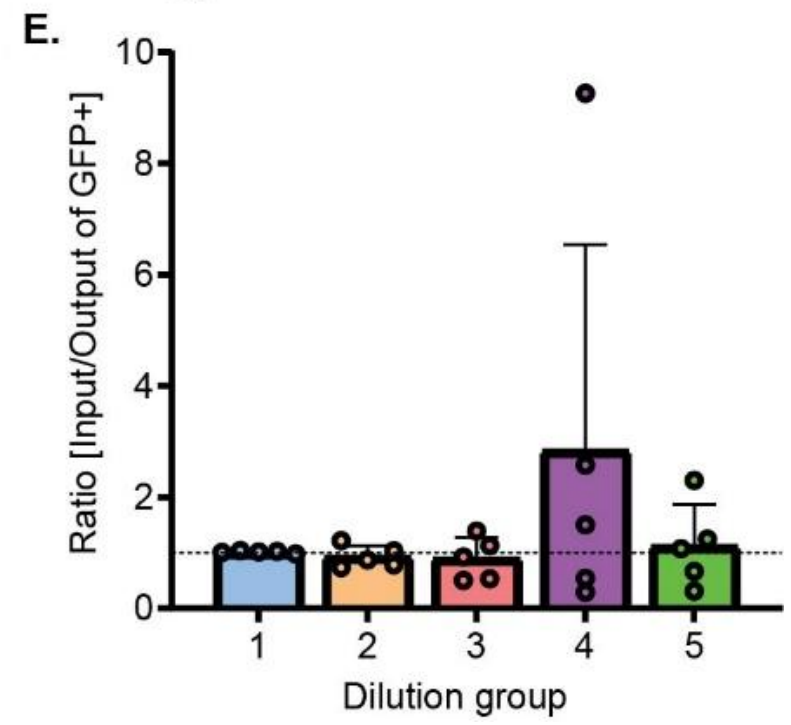

Figure S2: A PDAC model amenable for *in vivo* CRISPR-Cas9 KO screening. A) Experimental schematic: Testing the selective pressure conferred by CAR T cell treatment *in vitro* in a CAR T cell cytotoxicity assay. PDAC cells with hCD19 antigen expression (GFP+) were mixed at a 1:1 ratio with hCD19-negative PDAC cells (E2-Crimson+). Next, 10,000 PDAC cells from this mixture were incubated with 100,000 hCD19 CAR T cells or control (EGFRvIII) CAR T cells for 24 hours, followed by flow cytometric analysis.* **B)** A bar graph showing the change in the percentage of hCD19-negative cells in a mixture of antigen-positive and -negative PDAC cells after CAR T cell treatment. Only treatment with hCD19 CAR T cells causes enrichment of antigen-negative PDAC cells. All conditions were analyzed in triplicate and error bars represent standard deviation. Statistical analysis with Ordinary One-Way Anova test (ns > 0.05, * ≤ 0.05, ** p ≤ 0.01, *** p ≤ 0.001, **** p ≤ 0.0001). **C)** Experimental schematic: Determining the maximal CRISPR library size that can be represented in a murine PDAC tumor model *in vivo* using a GFP dilution assay. Diminishing amounts of GFP+ hCD19+ PDAC cells were spiked into an mCherry+ hCD19+ PDAC cell population at the indicated percentages. A total of 600,000 hCD19+ PDAC cells was then orthotopically transplanted into C57BL/6 mice. In total, five experimental groups containing hCD19+ PDAC cells with GFP percentages ranging from 50% to 0.025% were transplanted into mice (n=5 per experimental group). After ten days, tumors were isolated, dissociated into single cell solutions and

analyzed for mCherry+ and GFP+ tumor cells by flow cytometry. The percentage of recovered GFP+ cells (output) was then analyzed and compared to the initial GFP+ tumor cell percentage prior to injection (input).* **D)** Table showing the five experimental groups and their inferred representation of CRISPR library size. The largest CRISPR library that can be represented in this hCD19+ PDAC tumor model is 4000 sgRNAs (150x representation) in a total of 600,000 tumor cells. **E)** A bar graph showing the ratio of initial percentage of GFP+ PDAC cells (input) to the GFP+ PDAC cell percentage at the experimental endpoint (output). A ratio ≥1 indicates that the GFP percentage remained stable throughout the entirety of the experiment. **Created in BioRender. Hemann, M. (2026) https://BioRender.com/uu8rpk2*.

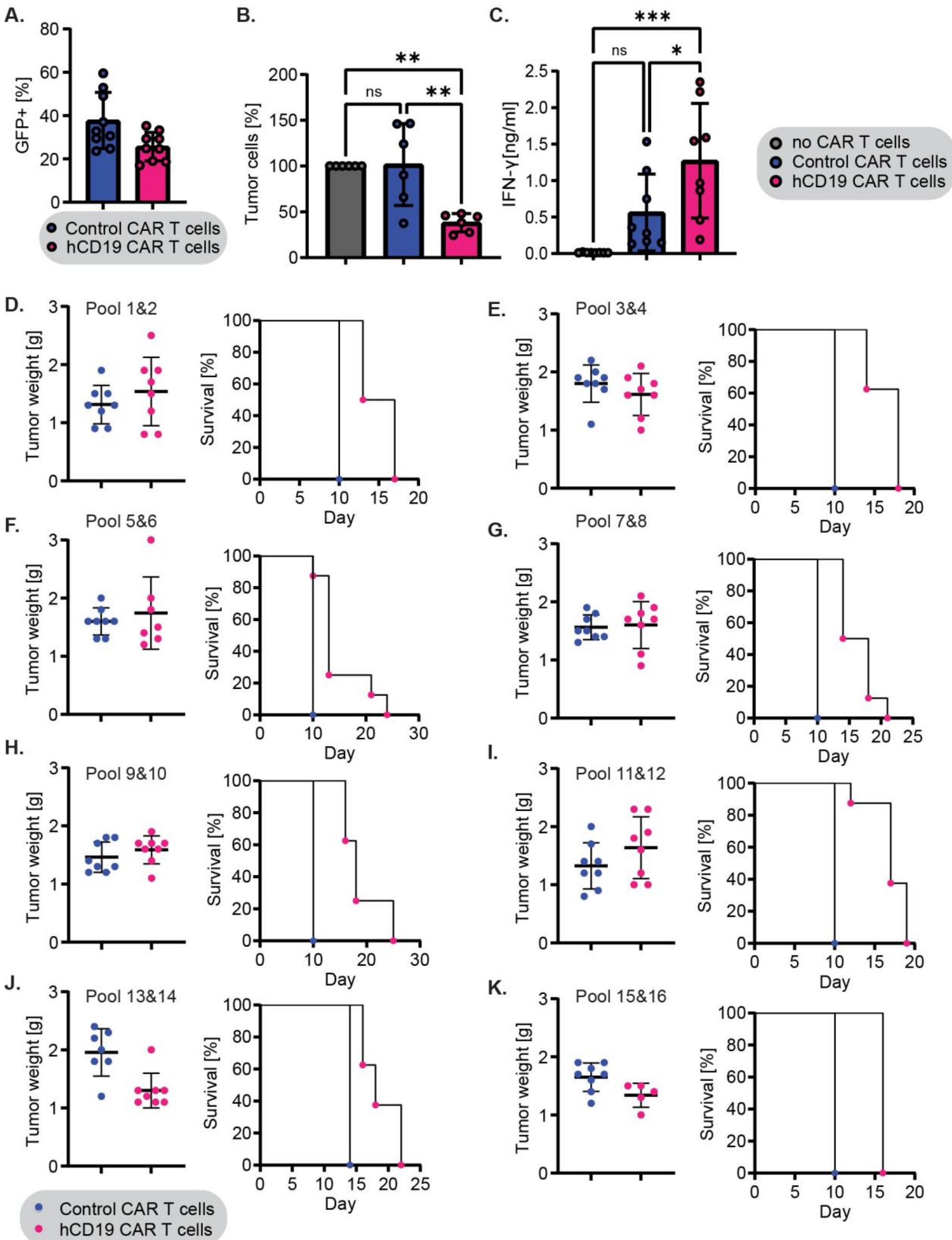

Figure S3: CAR T cell quality control data and tumor data from eight independent CRISPR-Cas9 KO screens. A) A bar graph showing the average transduction rate of murine CD8+ T cells with CAR-GFP constructs. **B)** A bar graph showing the average CAR T cell killing across six independent CAR T cell

cytotoxicity assays involving incubation of hCD19 CAR T cells or control (EGFRvIII) CAR T cells with PDAC cells (E:T ratio = 10:1) for 24 hours. All conditions were analyzed in triplicate. Error bars represent standard deviation. Statistical analysis with Ordinary One-Way ANOVA test (ns > 0.05, * ≤ 0.05, ** $p \leq 0.01$, *** $p \leq 0.001$, **** $p \leq 0.0001$). **C)** A bar graph showing the average IFN-γ release by hCD19 or control CAR T cells during the CAR T cell cytotoxicity assays across six independent experiments. All conditions were analyzed in triplicate and error bars represent standard deviation. Statistical analysis conducted using Ordinary One-Way ANOVA test (ns > 0.05, * ≤ 0.05, ** $p \leq 0.01$, *** $p \leq 0.001$, **** $p \leq 0.0001$). **D-K)** Average tumor weights (left) and tumor day harvests (right) shown in Kaplan-Meier format for each independent CRISPR-Cas9 KO screen. D) Screen 1 with CRISPR library pools 1&2, E) Screen 2 with CRISPR library pools 3&4 , F) Screen 3 with CRISPR library pools 5&6, G) Screen 4 with CRISPR library pools 7&8, H) Screen 5 with CRISPR library pools 9&10, I) Screen 6 with CRISPR library pools 11&12, J) Screen 7 with CRISPR library pools 13&14, K) Screen 8 with CRISPR library pools 15&16.

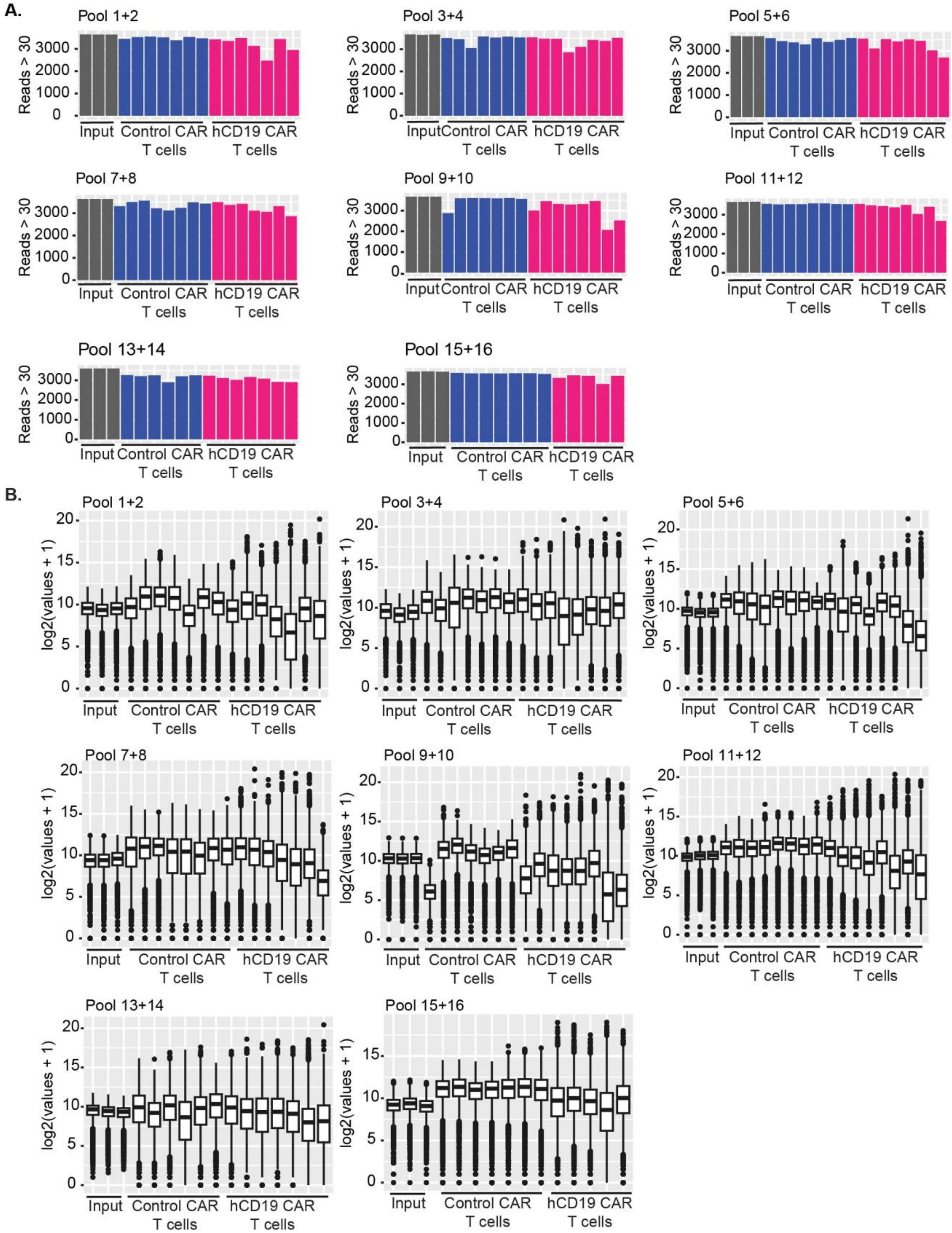

Figure S4: Analysis of eight independent *in vivo* CRISPR-Cas9 KO screens. A) A bar graph showing the number of represented sgRNA per mouse for each independent CRISPR-Cas9 KO screen. The number

of sgRNAs with at least 30 reads is shown for PDAC cells containing the CRISPR libraries on the day of injection (input) compared to tumor cells isolated from mice treated with hCD19 CAR T cells or treated with control CAR T cells. **B)** Box plots showing the distribution of raw sgRNA counts in PDAC cells containing the CRISPR libraries on the day of injection (input) compared to tumor cells isolated from mice treated with hCD19 CAR T cells or treated with control CAR T cells for each independent CRISPR-Cas9 KO screen.

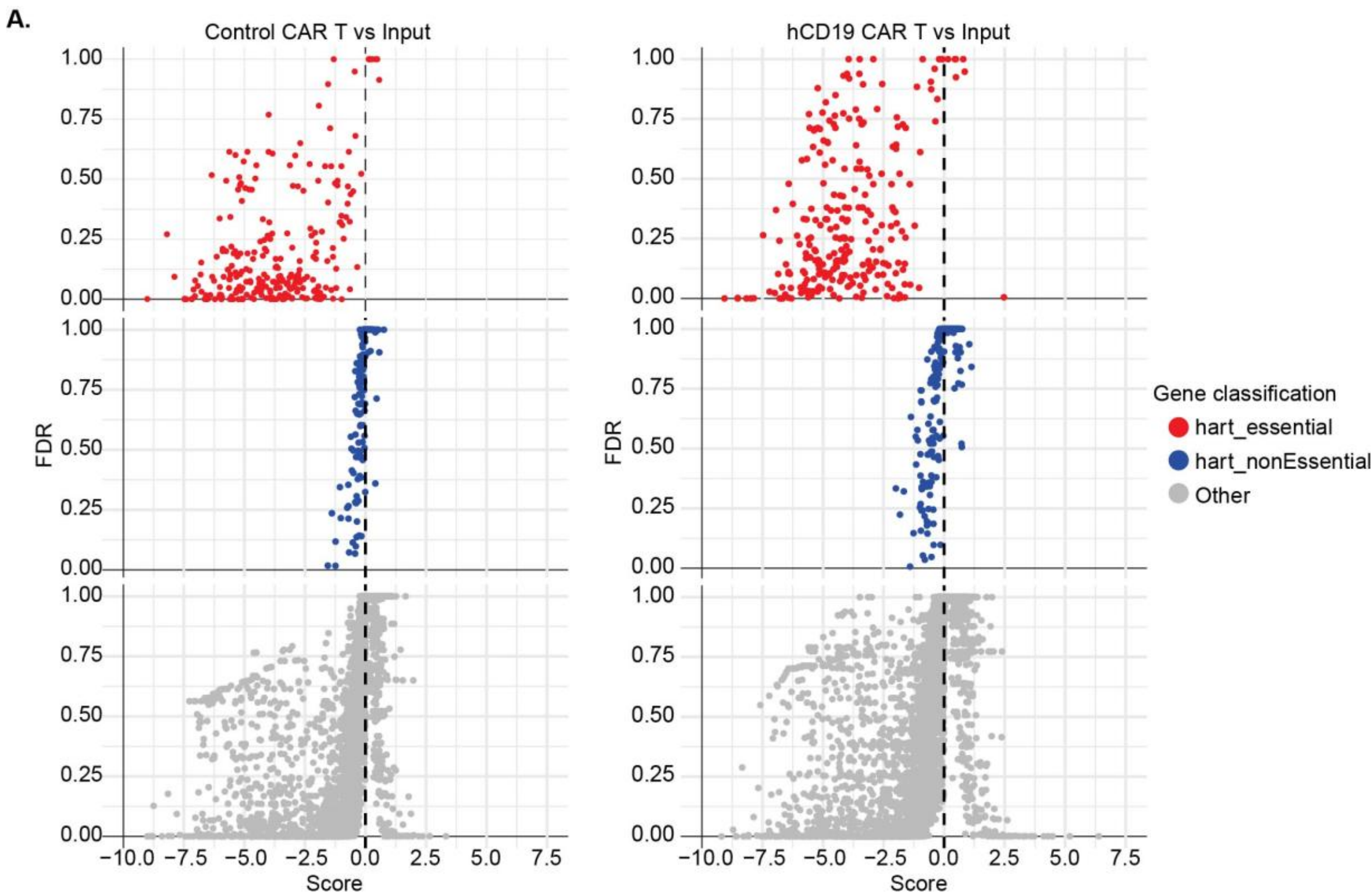

Figure S5: Essential genes show predicted behavior *in vivo*. Scatter plot highlighting the behavior of sgRNAs targeting genes classified as essential or non-essential, as defined by Hart et. al, compared to sgRNAs targeting all remaining genes in the first CRISPR-Cas9 KO screen (Pool 1 & 2) in control CAR T cell treated and hCD19 CAR T cell treated mice compared to tumor cells on the day of injection (input). FDR = False discovery rate, Score = Log2 fold change.

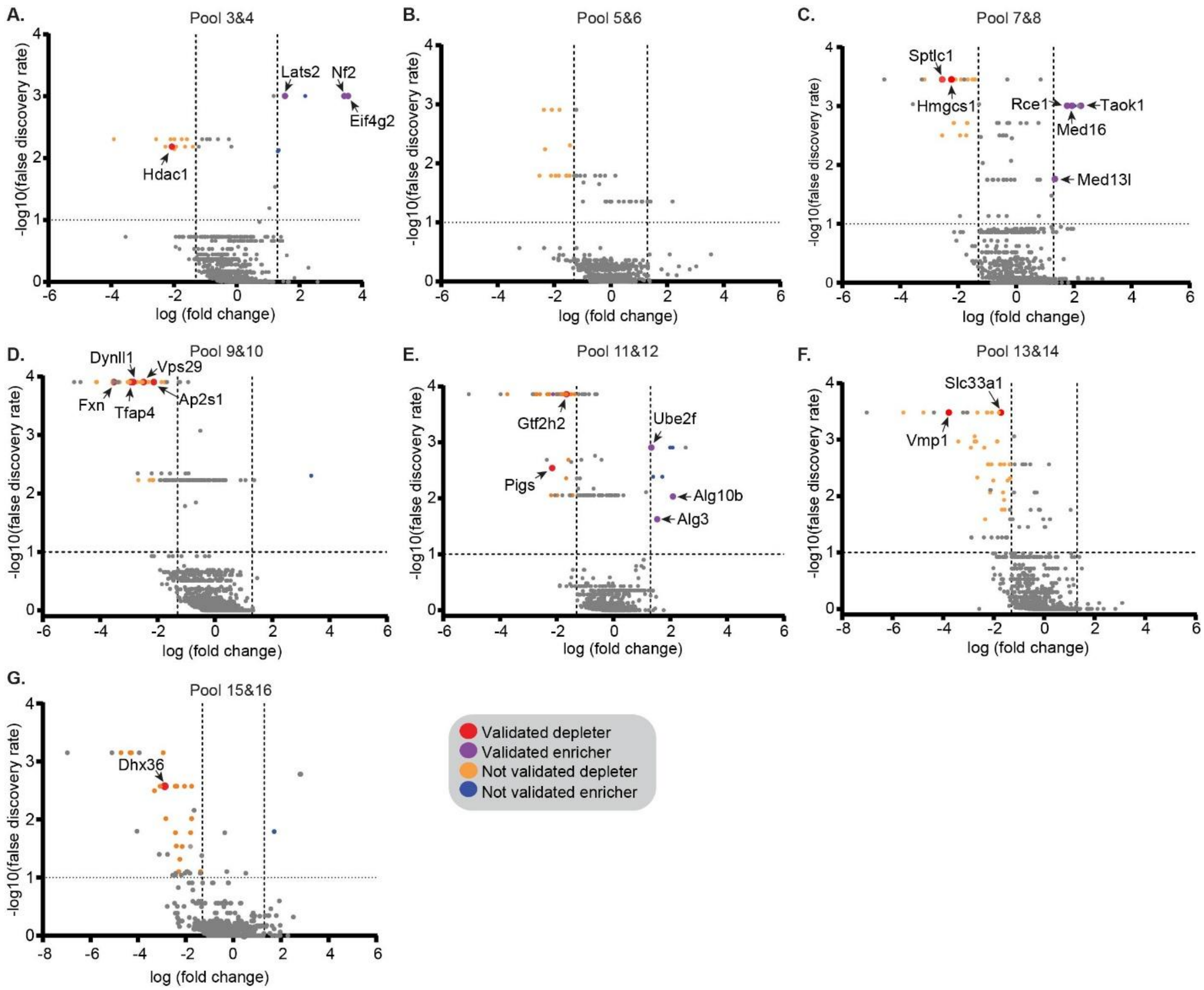

Figure S6: Iterative in vivo CRISPR-Cas9 screening reveals tumor cell-intrinsic mediators of CAR T cell response. Volcano plots showing the results of the knock-out screens 2 through 8. “Validated depleter/enricher” refers to genes that were validated in a secondary CRISPR-Cas9 KO screen. “Not validated depleter/enricher” refers to genes that were not confirmed as hits in a secondary CRISPR-Cas9 KO screen. Validated enrichers/depleters are additionally highlighted by name. **A)** Screen 2: Pool 3&4, **B)** Screen 3: Pool 5&6, **C)** Screen 3: Pool 7&8, **D)** Screen 4: Pool 9&10, **E)** Screen 5: Pool 11&12, **F)** Screen 7: Pool 13&14, **G)** Screen 8: Pool 15&16.

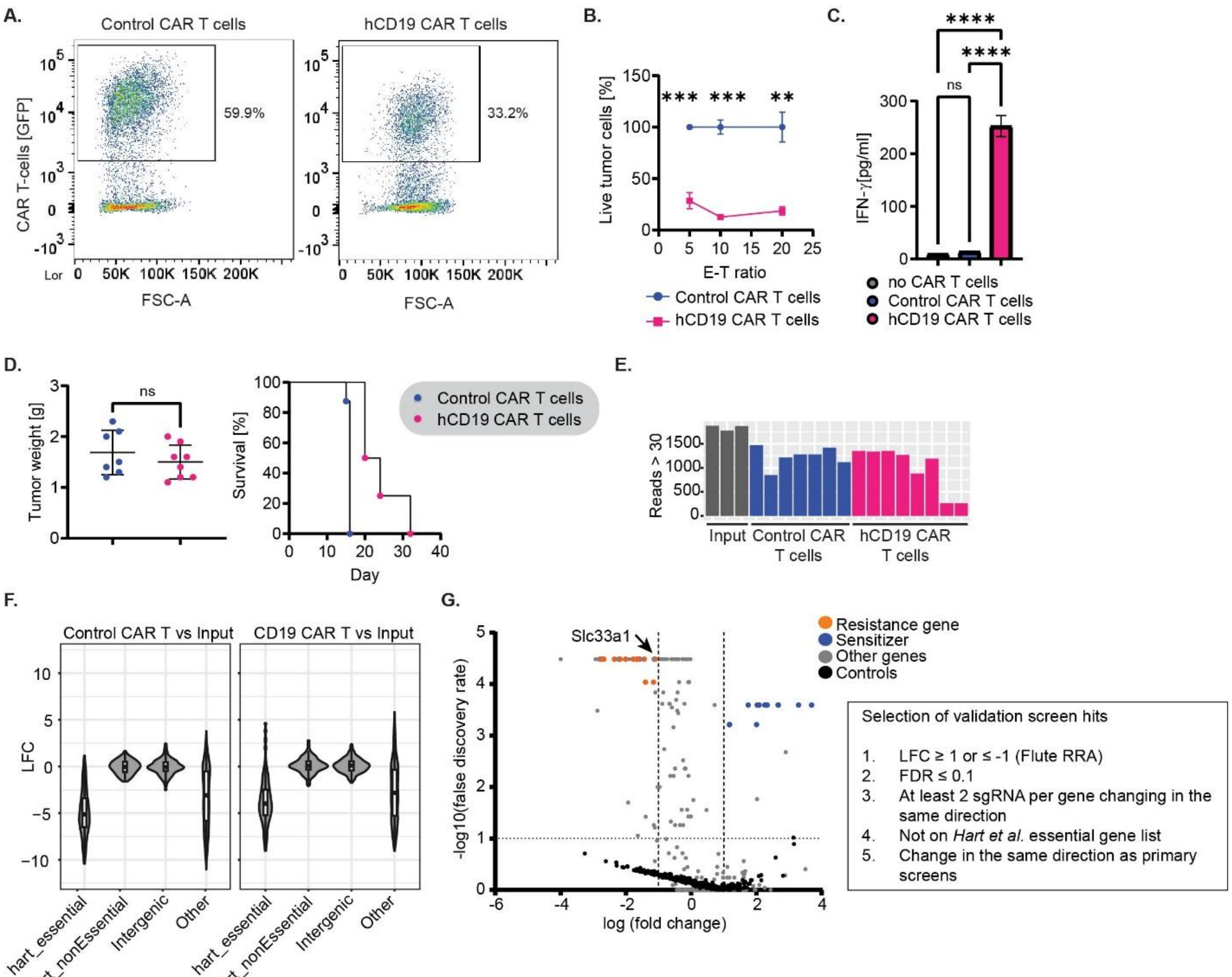

Figure S7: Validation CRISPR-Cas9 KO screen. A) A flow cytometry plot showing CAR T cell production efficiency for validation screen. Flow cytometric analysis of CD8+ T cell transduction rates with CAR-GFP constructs. **B)** A graph showing results from a CAR T cell cytotoxicity assay (on the day of *in vivo* injection) involving incubation of hCD19 CAR T cells or control (anti-EGFRvIII) CAR T cells with PDAC cells at multiple effector-to-target ratios (E:T ratios = CAR T cells:PDAC cells = 5:1, 10:1, 20:1) for 24 hours. All conditions were analyzed in triplicate and error bars represent standard deviation. Statistical analysis conducted using unpaired t-test. **C)** A bar graph showing the level of IFN-γ release by hCD19 or control CAR T cells during the CAR T cell assay (E:T ratio = 10:1). All conditions were analyzed in triplicate and error bars represent standard deviation. Statistical analysis conducted using Ordinary One-Way ANOVA (ns > 0.05, * ≤ 0.05, ** p ≤ 0.01, *** p ≤ 0.001, **** p ≤ 0.0001). **D)** Average tumor weights (left) and tumor day harvests (right) as shown in Kaplan-Meier format for the validation CRISPR-Cas9 KO screen. Statistical analysis conducted using Mann-Whitney test. **E)** A bar graph showing the number of represented sgRNA per mouse for each independent CRISPR-Cas9 KO screen. The number of sgRNAs with at least 30 reads is shown for PDAC cells containing the validation CRISPR library on the day of injection (input) compared to tumor cells isolated from mice treated with hCD19 CAR T cells or treated with control CAR T cells. **F)** Violin plots showing the behavior of sgRNAs targeting genes classified as essential or non-essential, as defined by Hart et. al, compared to sgRNAs targeting intergenic regions or all remaining genes in control CAR T cell treated and hCD19 CAR T cell treated mice compared to tumor cells on the day of injection (input). **G)** A volcano plot showing the results of the validation screen. Hits

from this screen are split up into “resistance” (orange) and “sensitizer” (blue) genes. To be considered a “hit” each targeted gene had to fulfill five criteria: (1) a LFC of greater than 1 or smaller than -1, (2) a FDR value equal to or smaller than 0.1, (3) at least two out of six guide RNAs changing in the same direction, (4) not considered essential (as defined by Hart et al.), (5) and sgRNAs changed in the same direction as in the original CRISPR-Cas9 KO screens.

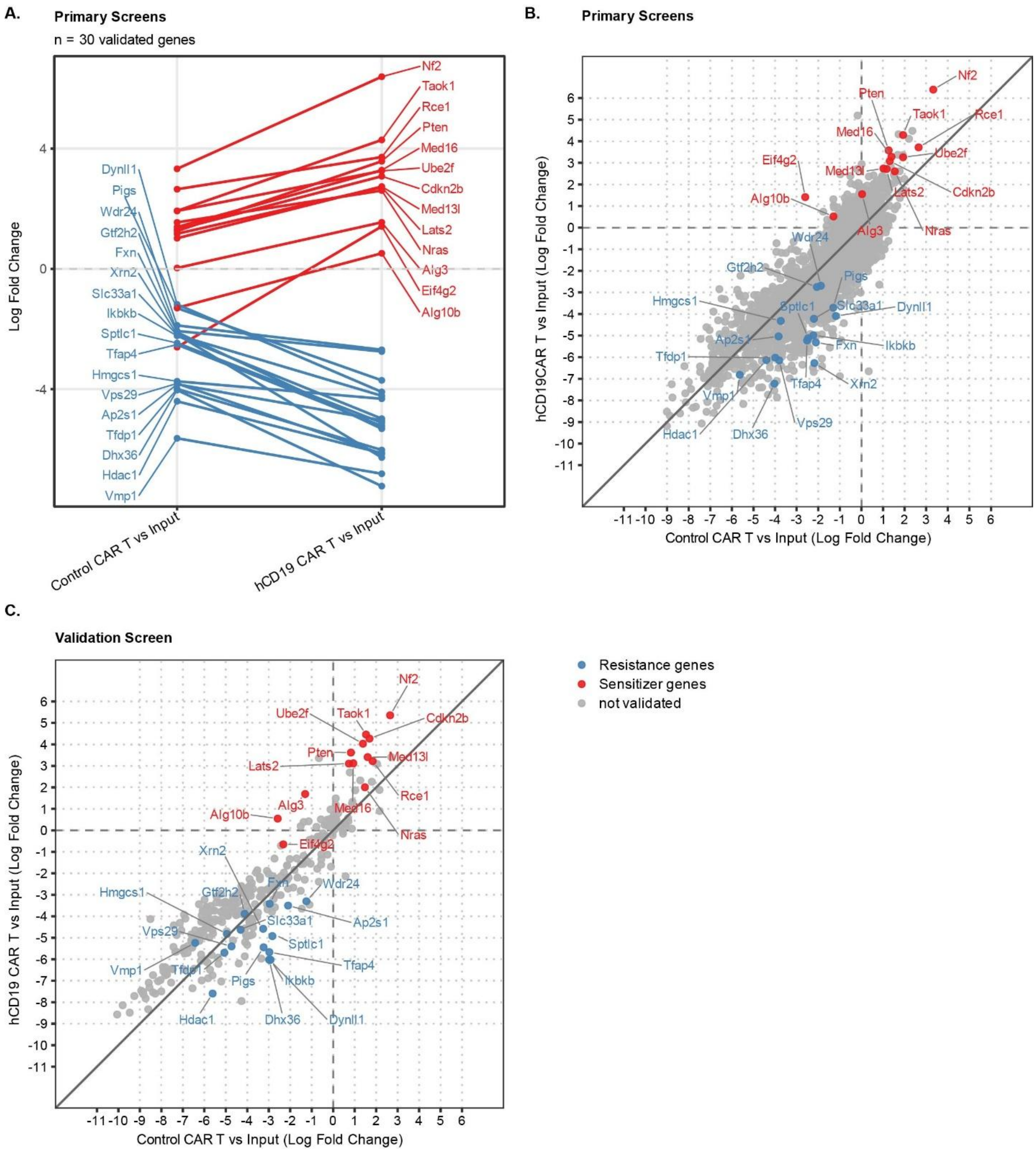

Figure S8: MAGeCK analysis of median log fold changes in sgRNAs targeting CAR T cell modulator genes *in vitro* vs *in vivo*. A) Median log-fold changes of sgRNAs for each gene identified as a CAR T cell modulator in input samples (*in vitro*) compared to control (EGFRvIII) or hCD19 CAR T cell-treated samples (*in vivo*) for all primary CRISPR screens. **B)** Scatter plot showing median log fold changes of sgRNAs for all genes in the primary screens comparing sgRNA changes in input samples vs control CAR T cell treatment and input samples vs hCD19 CAR T cell treatment. Genes modulating CAR T cell response subsequently validated in a secondary screen are highlighted in red (sensitizers) and blue (resistance genes). **C)** Scatter plot showing median log fold changes of sgRNAs for all genes in the validation screen comparing

sgRNA changes in input samples vs control CAR T cell treatment and input samples vs hCD19 CAR T cell treatment. Genes modulating CAR T cell response are highlighted in red (sensitizers) and blue (resistance genes).

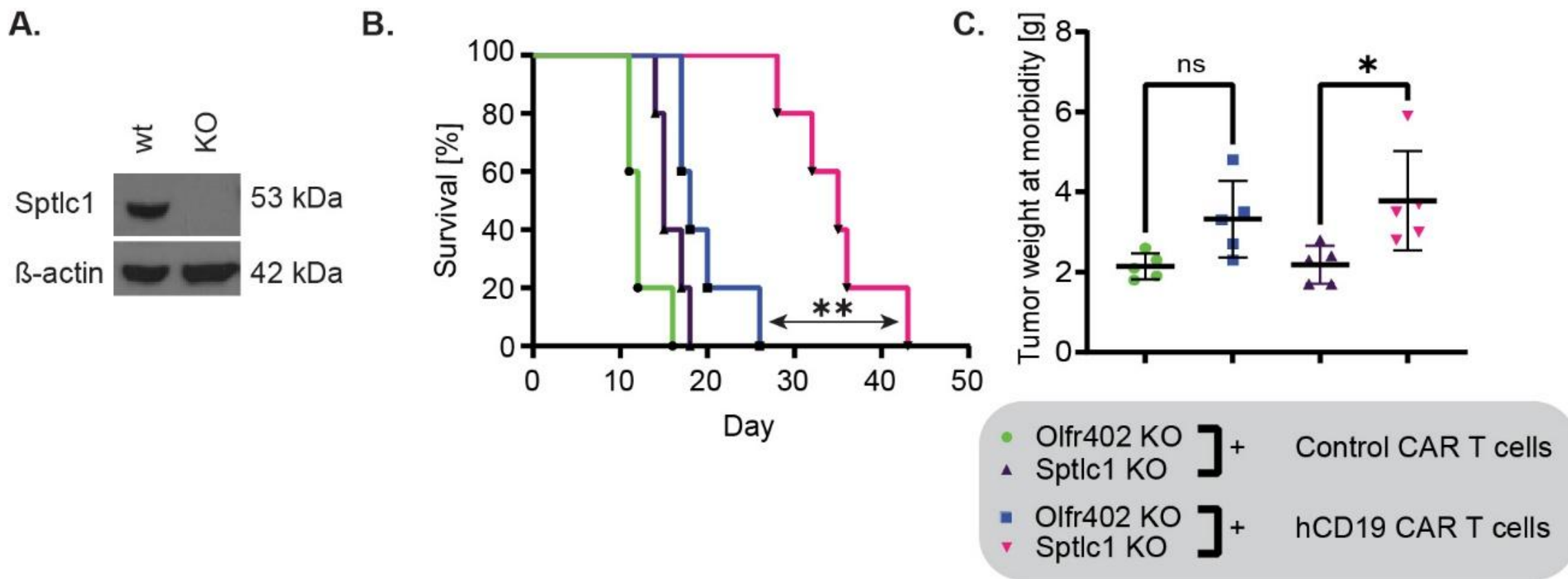

Figure S9: Loss of *Sptlc1* sensitizes to CAR T-cell therapy *in vivo*. A) Western blot confirming *Sptlc1* knock-out in PDAC cells. **B)** Kaplan-Meier curve: 6×10^5 PDAC cells (control KO or *Sptlc1* KO) injected into C57BL/6 mice and treated with hCD19 or control (EGFRvIII) CAR T cells (5×10^6; n=10 per group) via i.p. injection on day 4. Tumors were harvested when a humane endpoint was reached. *Sptlc1* KO tumors show extended survival following hCD19 CAR T treatment. Mantel–Cox test (p=0.0018). **C)** Tumor weight at morbidity: S*ptlc1* KO tumors treated with hCD19 CAR T cells are significantly larger. Error bars represent means ± SD. Ordinary one-way ANOVA (p=0.0464).

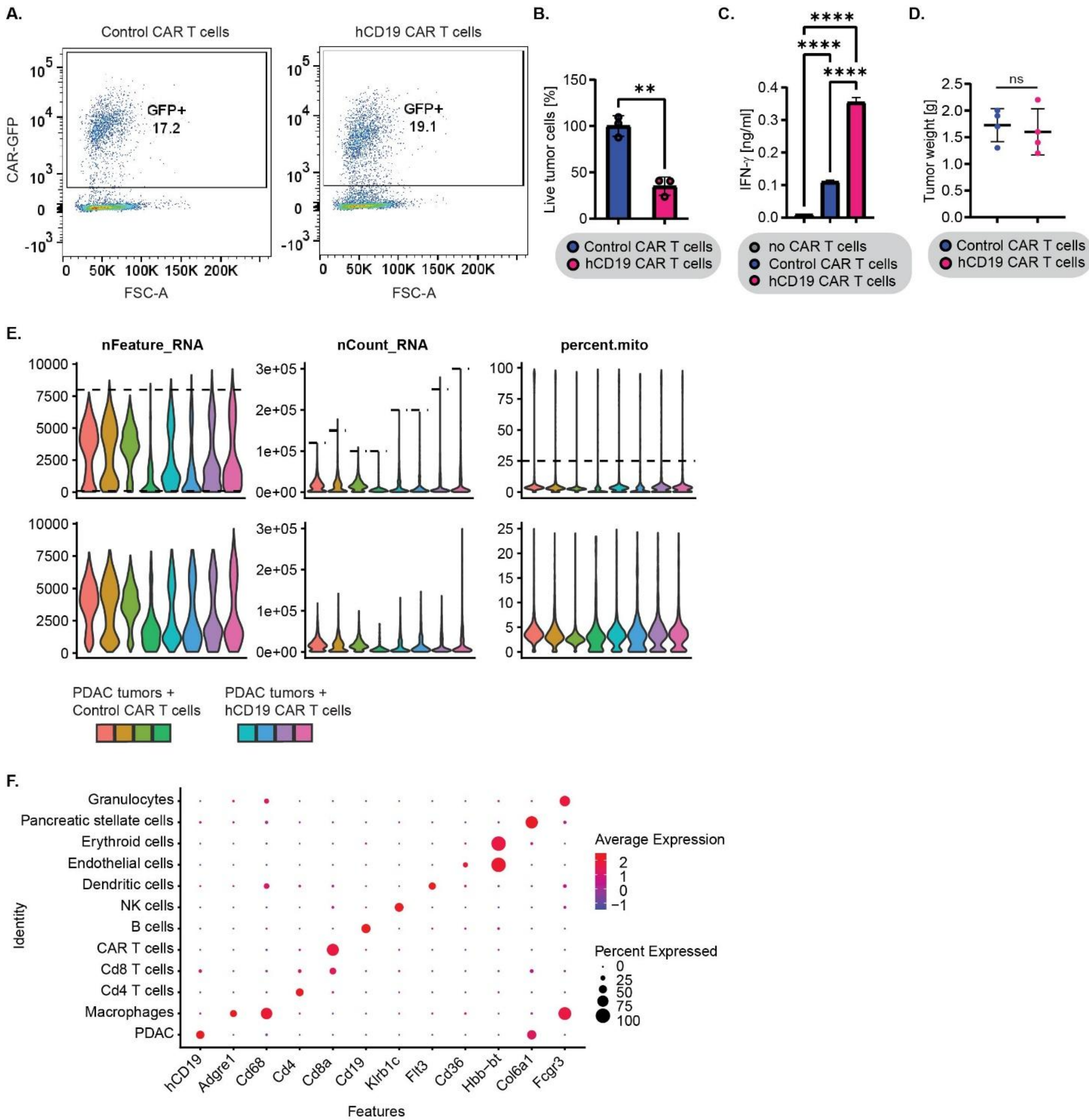

Figure S10: Single cell RNA sequencing of CAR T cell treated PDAC tumors. A) Flow cytometric analysis of CD8+ T cell transduction rates with CAR-GFP constructs. **B)** A bar graph showing data from a CAR T cell cytotoxicity assay involving incubation of hCD19 CAR T cells or control (anti-EGFRvIII) CAR T cells with hCD19+ PDAC cells at a 10:1 effector-to-target ratio for 24 hours. All conditions were analyzed in triplicate and error bars represent standard deviation. Statistical analysis conducted using unpaired t-test (ns > 0.05, * ≤ 0.05, ** p ≤ 0.01, *** p ≤ 0.001, **** p ≤ 0.0001). **C)** A bar graph showing the level of IFN-γ release by hCD19 or control CAR T cells during the CAR T cell assay. All conditions were analyzed in triplicate and error bars represent standard deviation. Statistical analysis conducted using

Ordinary One-Way ANOVA (ns > 0.05, * ≤ 0.05, ** $p \leq 0.01$, *** $p \leq 0.001$, **** $p \leq 0.0001$). **D)** A graph showing the tumor weight of PDAC tumors treated with control (isolated on day 10) or hCD19 (isolated on day 15) CAR T cells. Tumors are similar in size at the day of isolation. Statistical analysis conducted using t-test (ns > 0.05). **E)** Violin plots showing single cell RNA sequencing data for each PDAC tumor before (top) and after (bottom) filtering of the number of genes detected in each cell **(=**nFeature_RNA >100, <8000), the total number of molecules detected within a cell **(=**nCount_RNA; top 10% removed) and percent mitochondrial reads (=percent.mito; <25%). **F)** Identification of cell clusters in single cell RNA sequencing data. The dot plot is showing the expression levels of multiple genes across tumor cells and stromal cells used for cell type identification.

A.

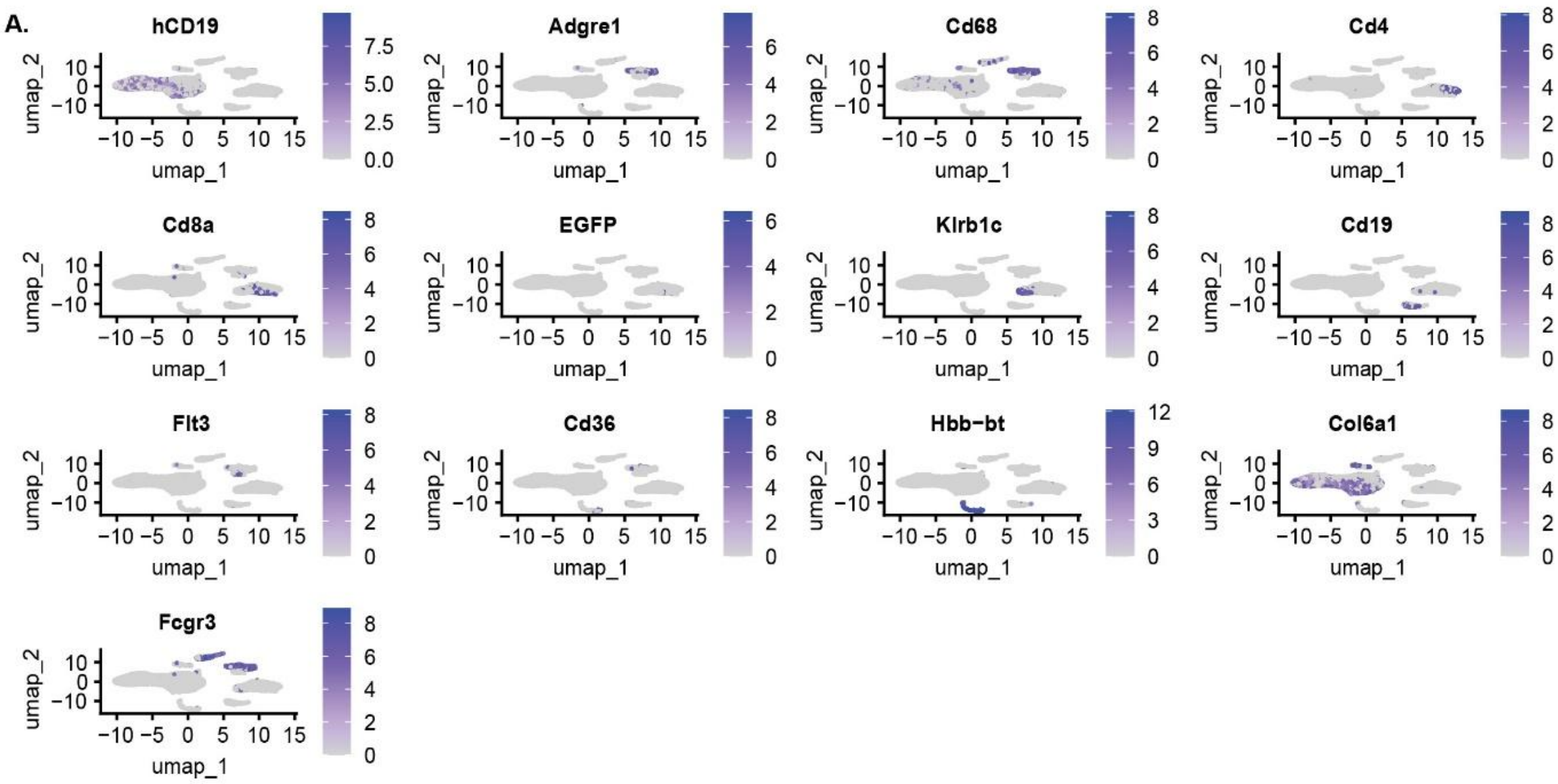

B.

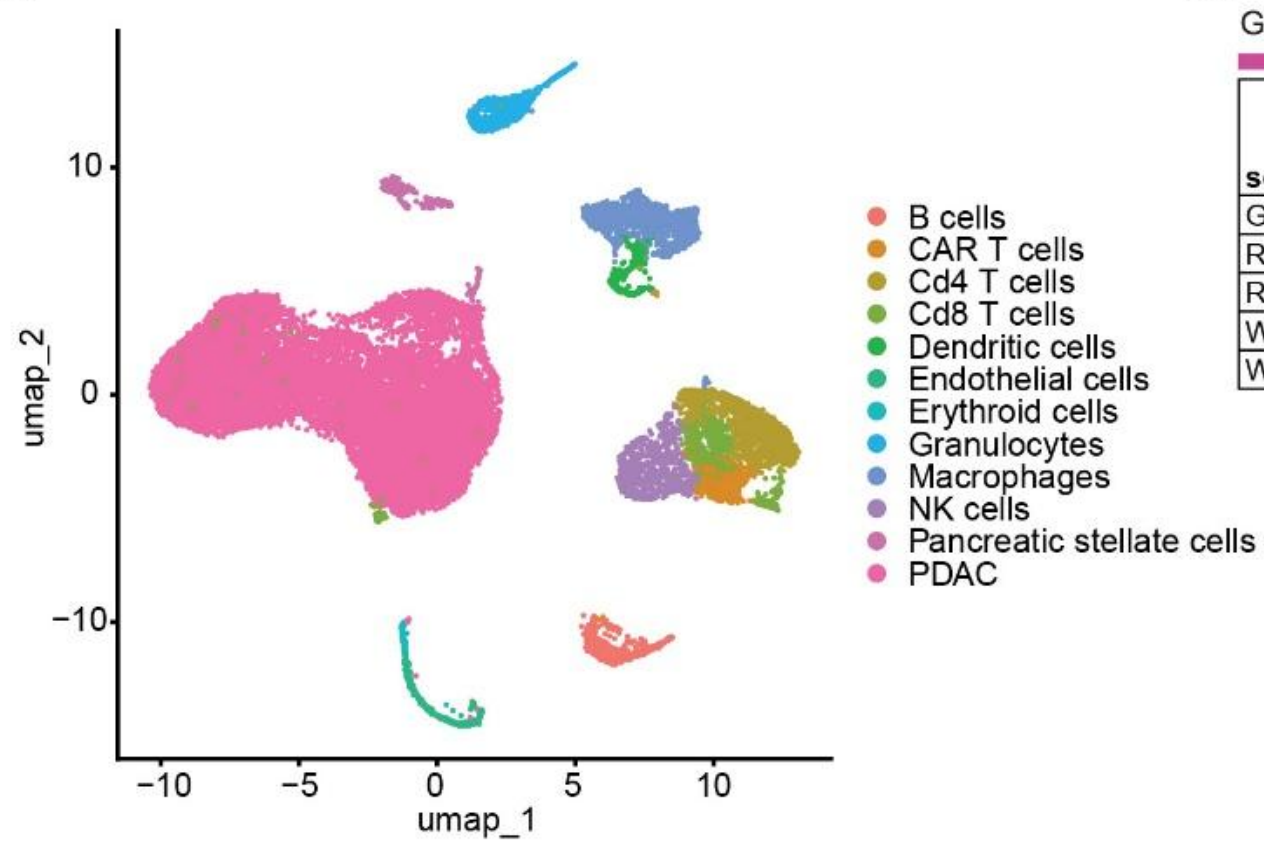

C.

GO terms enriched in hCD19 CAR T cell-treated PDAC tumors

source	term_name	term_id	adjusted_ p_value
GO:MF	extracellular matrix structural constituent	GO:0005201	0.0194
REAC	Extracellular matrix organization	REAC:R-HSA-1474244	0.0001
REAC	Non-integrin membrane-ECM interactions	REAC:R-HSA-3000171	0.0035
WP	Wnt beta catenin signaling in leukemia	WP:WP3658	0.0385
WP	Acute myeloid leukemia	WP:WP5293	0.0477

Figure S11: Single cell RNA sequencing cluster annotation and gene ontology. A) Feature plots showing the expression of different genes across all clusters of cells isolated from PDAC tumors in a UMAP. These are examples of genes that were used for the identification and annotation of the shown cell clusters. **B)** A UMAP showing the annotated tumor and stromal cell clusters isolated from PDAC tumors. **C)** Gene ontology (GO) analysis of top 100 enriched genes in hCD19 CAR T cell treated tumors compared to control CAR T cell treated tumors.

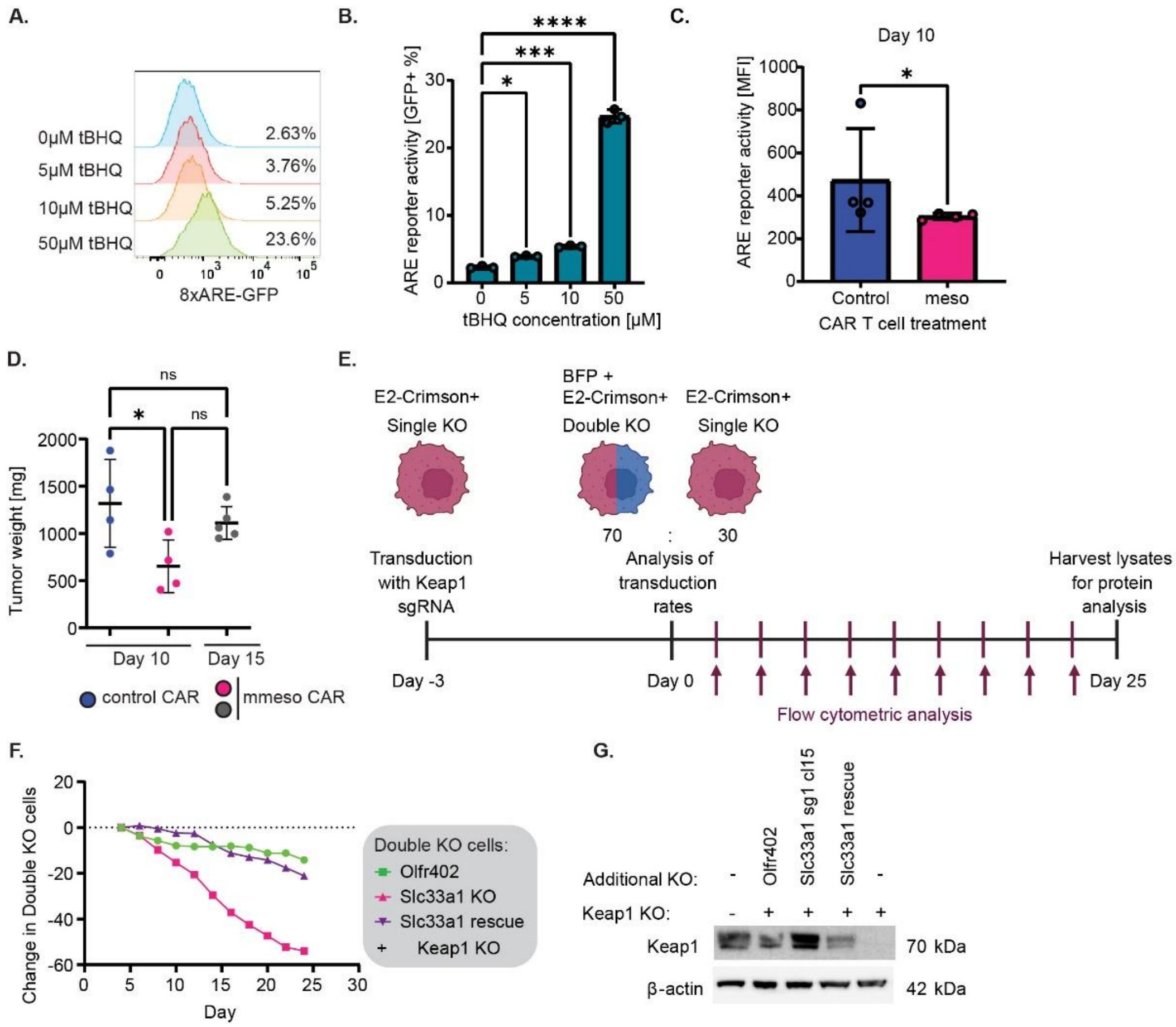

Figure S12: *Nrf2*-activity in PDAC cells. A) Flow cytometry showing *Nrf2* reporter expression. Treatment of WT PDAC cells expressing an *NRF2* 8xARE-GFP reporter with the Nrf2 activator tBHQ for 24 hours *in vitro* leads to dose-dependent expression of GFP as measured by flow cytometry. **B)** A bar graph showing quantification of *NRF2* 8xARE-GFP reporter activity (as % GFP) after treatment with different doses of tBHQ for 24 hours. All conditions were analyzed in triplicate and error bars represent standard deviation. Statistical analysis conducted using Ordinary One-Way ANOVA (ns > 0.05, * ≤ 0.05, ** p ≤ 0.01, *** p ≤ 0.001, **** p ≤ 0.0001). **C)** Mean Fluorescent Intensity (MFI) of *NRF2* 8xARE-GFP reporter PDAC cells in isolated tumors 10 days after tumor induction (n=4). Error bars represent standard deviation. Statistical analysis conducted using Mann-Whitney test (p=0.0286). **D)** A graph showing the weight of PDAC tumors formed by WT PDAC cells expressing an *NRF2* 8xARE-GFP reporter in C57BL/6 mice 10 days and 15 days after tumor cell injection. Statistical analysis with Ordinary One-Way ANOVA test (ns > 0.05, * ≤ 0.05, ** p ≤ 0.01, *** p ≤ 0.001, **** p ≤ 0.0001). **E)** Analysis of the effect of Keap1 KO on the proliferation of control (*Olfr402*) KO, *Slc33a1* KO or *Slc33a1* re-expression (rescue) *in vitro*. E2-Crimson+ PDAC cells with control KO, Slc33a1 KO or Slc33a1 rescue were transduced with a previously validated Keap1 sgRNA (BFP+). Three days after transduction, the transduction rates were analyzed by flow cytometry. Single KO and double KO cells were at an approximately 30:70 ratio at the beginning of the long-term co-culture. Throughout the co-culture, cells were analyzed every other day by flow cytometry. On day 25, lysates were harvested for protein analysis. *Created in BioRender. Hemann, M. (2026) https://BioRender.com/uu8rpk2*. **F)** A graph showing growth dynamics of Keap1 KO PDAC cells in a control (*Olfr402*) KO, *Slc33a1* KO or *Slc33a1* re-expression (rescue) background *in vitro*. The percentages of double KO cells were compared to the percentages of single KO cells over 25 days. *Keap1* and *Slc33a1*

double KO cells get depleted over time. **G)** A western blot showing Keap1 protein expression at the endpoint of long-term *in vitro* co-culture from (F). In the *Slc33a1* KO condition, *Slc33a1/Keap1* double-KO cells are depleted.

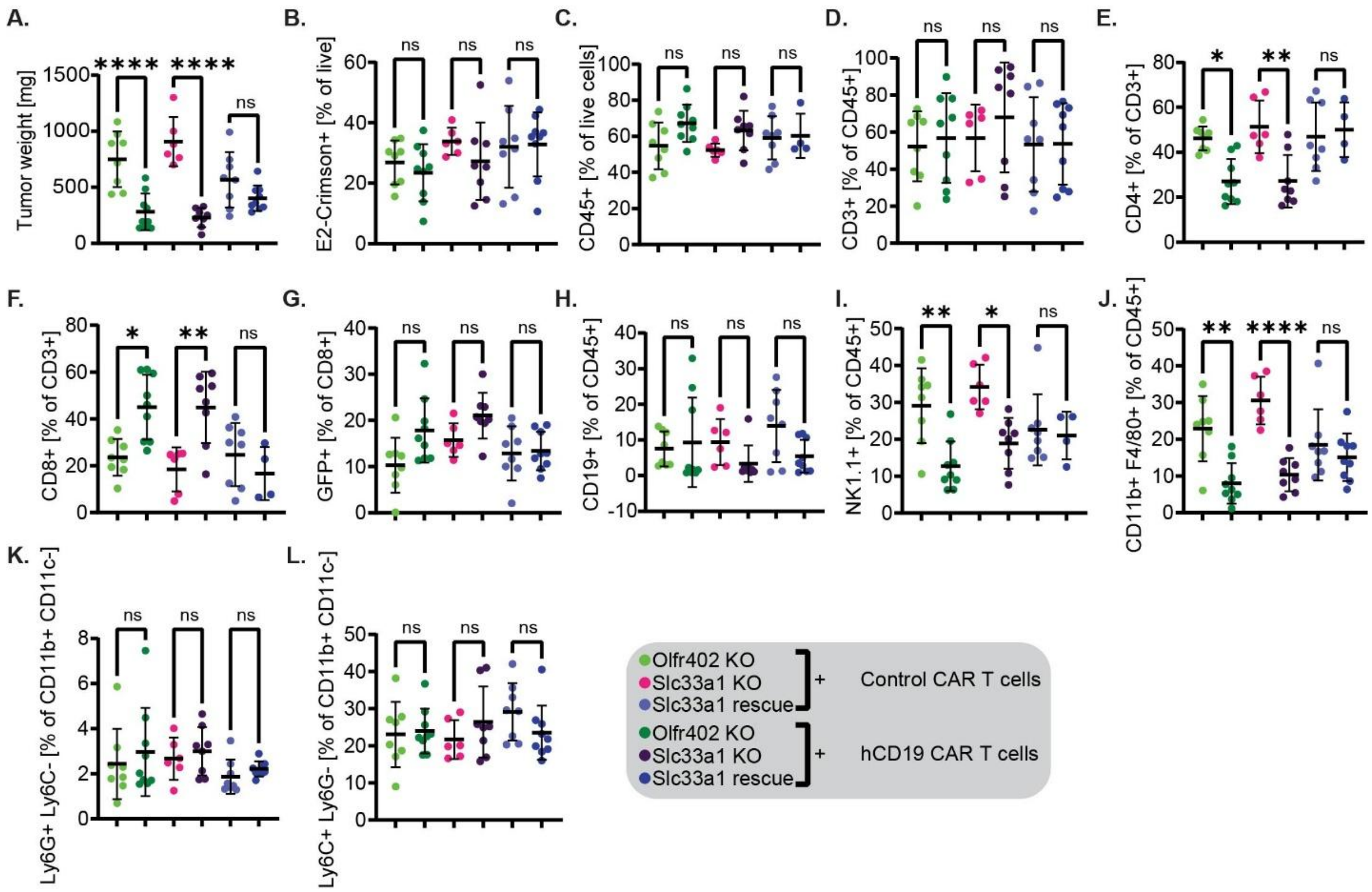

Figure S13: Analysis of *Slc33a1* KO tumor composition by flow cytometry. A) A graph showing the tumor weight of PDAC tumors with a control (*Olfr402*) KO, *Slc33a1* KO, or re-expression of *Slc33a1* (rescue), that were treated with control or hCD19 CAR T cells. All tumors were isolated on day 10 after tumor cell injection. PDAC tumors with *Slc33a1* or control KO treated with hCD19 CAR T cells are significantly smaller than control (EGFRvIII) CAR T cell treated tumors, but not *Slc33a1* rescue tumors. **B-L) Analysis of PDAC tumor composition by flow cytometry. B)** Percentage of E2-Crimson+ PDAC cells of all live cells. **C)** Percentage of CD45+ immune cells of all live cells. **D)** Percentage of CD3+ T cells of all immune cells. **E)** Percentage of CD4+ T cells of CD3+ T cells. CD4+ T cells are significantly reduced in PDAC tumors with control KO or *Slc33a1* KO treated with hCD19 CAR T cells compared to control CAR T cell treatment. **F)** Percentage of CD8+ T cells of CD3+ T cells. CD8+ T cells are significantly enriched in PDAC tumors with control KO or *Slc33a1* KO treated with hCD19 CAR T cells compared to control CAR T cell treatment. **G)** GFP+ CAR T cells as a percentage of all CD8 T cells. **H)** Percentage of CD19+ B cells of all immune cells. **I)** Percentage of NK1.1+ NK cells of all immune cells. **J)** Percentage of CD11b+ F4/80+ Macrophages of all immune cells. **K)** Percentage of Ly6G+ Ly6C- Neutrophils of all CD11b+ CD11c- immune cells. **L)** Percentage of Ly6C+ Ly6G- MDSCs of all CD11b+ CD11c- immune cells.

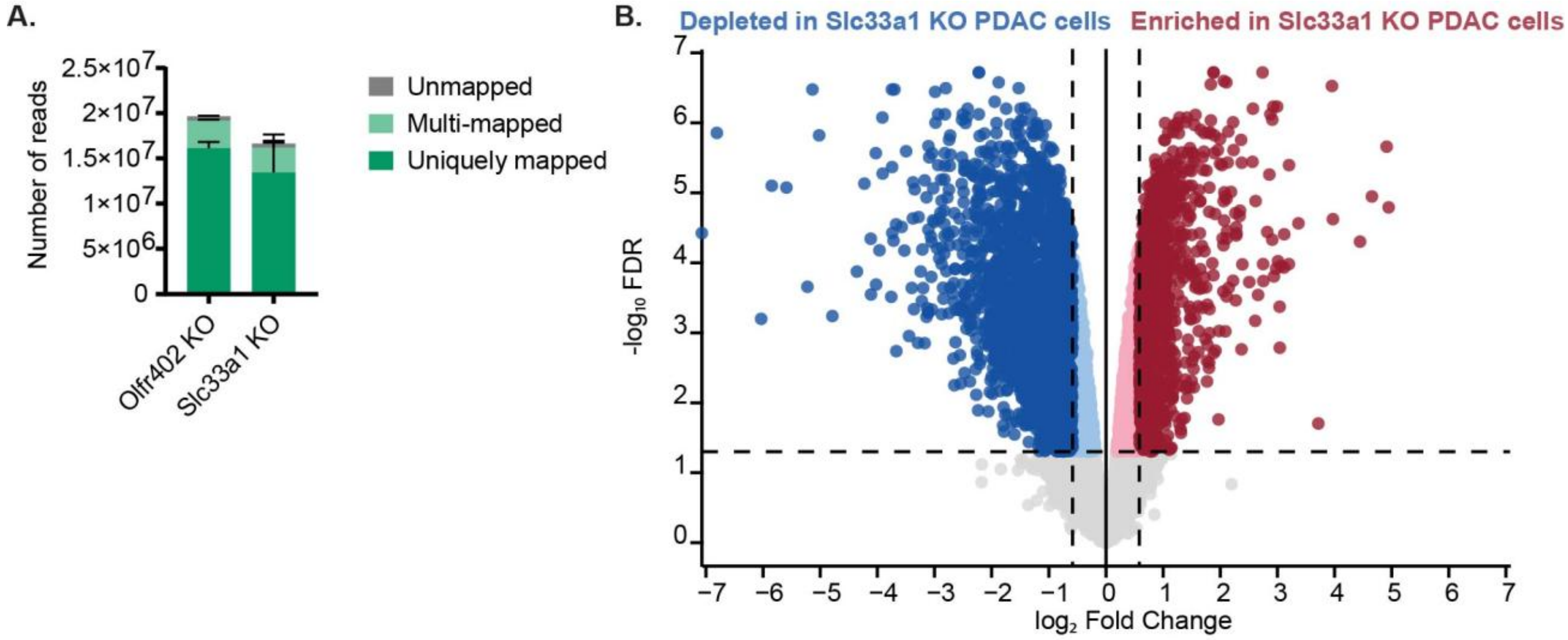

C.

Depleted in Slc33a1 KO PDAC cells

MSigDB Hallmark term	Size	NES	NOM p-value	FDR q-value
Interferon Gamma Response	102	-2.8781	0	0
Interferon Alpha Response	62	-2.835	0	0
IL6 JAK STAT3 Signaling	43	-2.2432	0	0
Allograft Rejection	57	-2.1142	0	0
Complement	67	-2.0055	0	0
Epithelial Mesenchymal Transition	60	-1.9226	0	0
Angiogenesis	10	-1.9174	0	0.000151143
Inflammatory Response	58	-1.893	0	0.000396751
Coagulation	36	-1.8159	0	0.000940448
Apical Junction	56	-1.777	0	0.001481205

Enriched in Slc33a1 KO PDAC cells

MSigDB Hallmark term	Size	NES	NOM p-value	FDR q-value
MYC Targets V1	145	2.96	0	0
Unfolded Protein Response	64	2.71872	0	0
MYC Targets V2	33	2.46046	0	0
E2F Targets	113	2.39803	0	0
MTORC1 Signaling	79	2.19919	0	0
Oxidative Phosphorylation	82	2.10713	0	0.000149276
DNA Repair	64	1.96955	0	0.000127951
G2M Checkpoint	80	1.8049	0	0.001343484
Adipogenesis	57	1.44245	0.003154574	0.027964373
Fatty Acid Metabolism	31	1.39581	0.014409222	0.038154948

Figure S14: Bulk tumor cell transcriptome profiling of *Slc33a1* KO PDAC cells. A) Bulk RNA sequencing read assignment for Control (*Olfr402*) or *Slc33a1* KO PDAC cells isolated from orthotopic tumors. The majority of reads are uniquely mapped. **B)** Volcano plot showing the differentially expressed genes of *Slc33a1* KO PDAC cells compared to control PDAC cells. Genes depicted in blue are depleted in *Slc33a1* KO cells compared to control KO cells. Genes depicted in red are enriched in *Slc33a1* KO cells compared to control KO cells. The cutoff for gene significance was set at a log fold change of |1.5| and an adjusted p-value of < 0.05. The y-axis depicts the -$\log_{10}$ FDR and the x-axis depicts the $\log_2$ fold change. **C)** GSEA analysis on differentially expressed genes in *Slc33a1* KO cells compared to control KO cells using the MSigDB Hallmark gene set. The top 10 pathways enriched (red) or depleted (blue) in *Slc33a1* KO cells compared to control KO cells are shown.

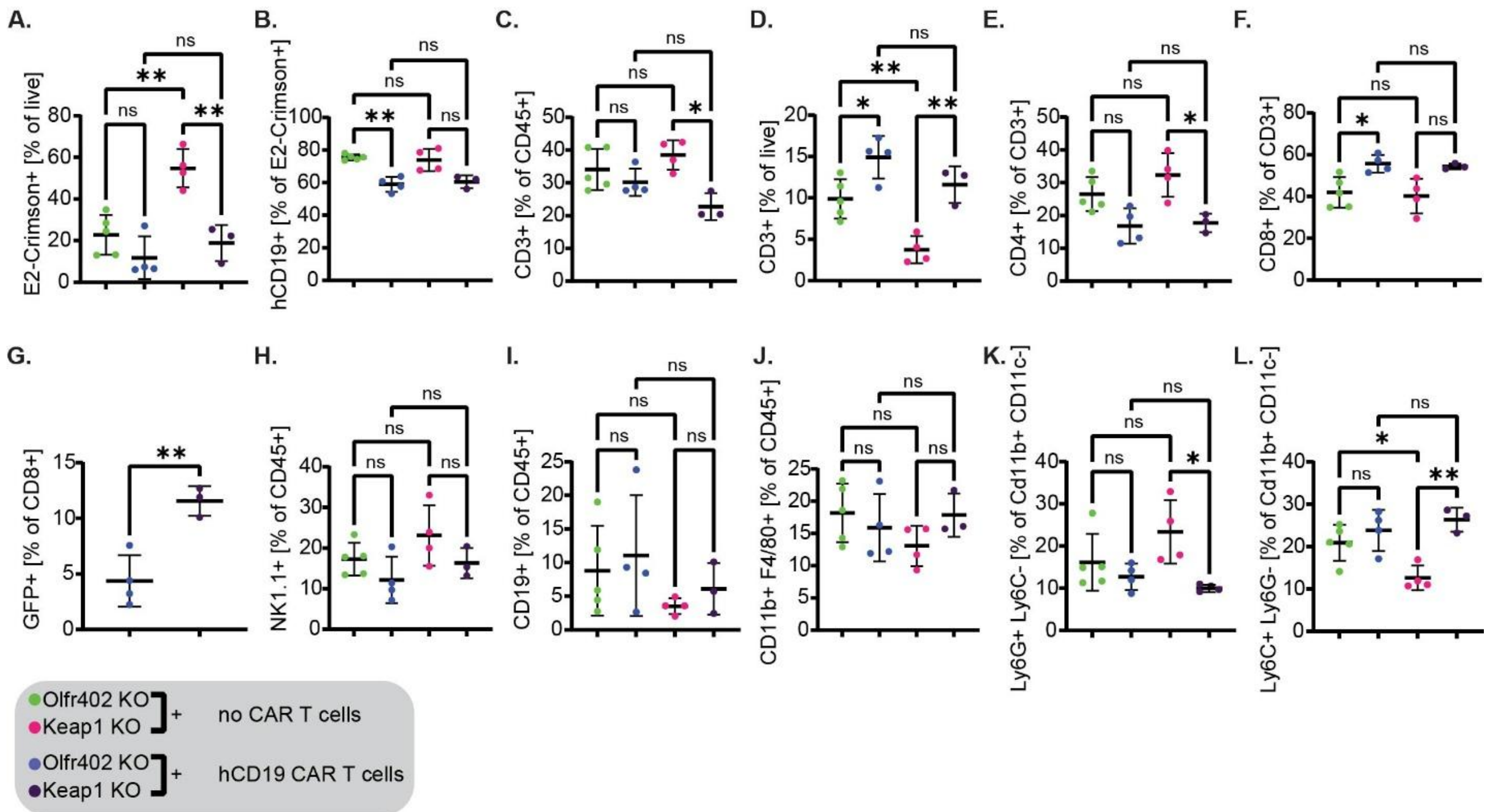

Figure S15: Analysis of *Keap1* KO tumor composition by flow cytometry. A) Percentage of E2-Crimson+ PDAC cells of all live cells. Untreated Keap1 KO tumors contain a significant higher proportion of PDAC cells. **B)** Percentage of antigen-positive (hCD19+) PDAC cells. **C)** Percentage of CD3+ T cells of all immune cells. **D)** Percentage of CD3+ T cells of all live cells. CD3+ tumor cells are enriched in hCD19 CAR T cell treated PDAC tumors . **E)** Percentage of CD4+ T cells of CD3+ T cells. **F)** Percentage of CD8+ T cells of CD3+ T cells. **G)** GFP+ CAR T cells as a percentage of all CD8 T cells. **H)** Percentage of NK1.1+ NK cells of all immune cells. **I)** Percentage of CD19+ B cells of all immune cells. **J)** Percentage of CD11b+ F4/80+ Macrophages of all immune cells. **K)** Percentage of Ly6G+ Ly6C- Neutrophils of all CD11b+ CD11c- immune cells. **L)** Percentage of Ly6C+ Ly6G- MDSCs of all CD11b+ CD11c- immune cells.

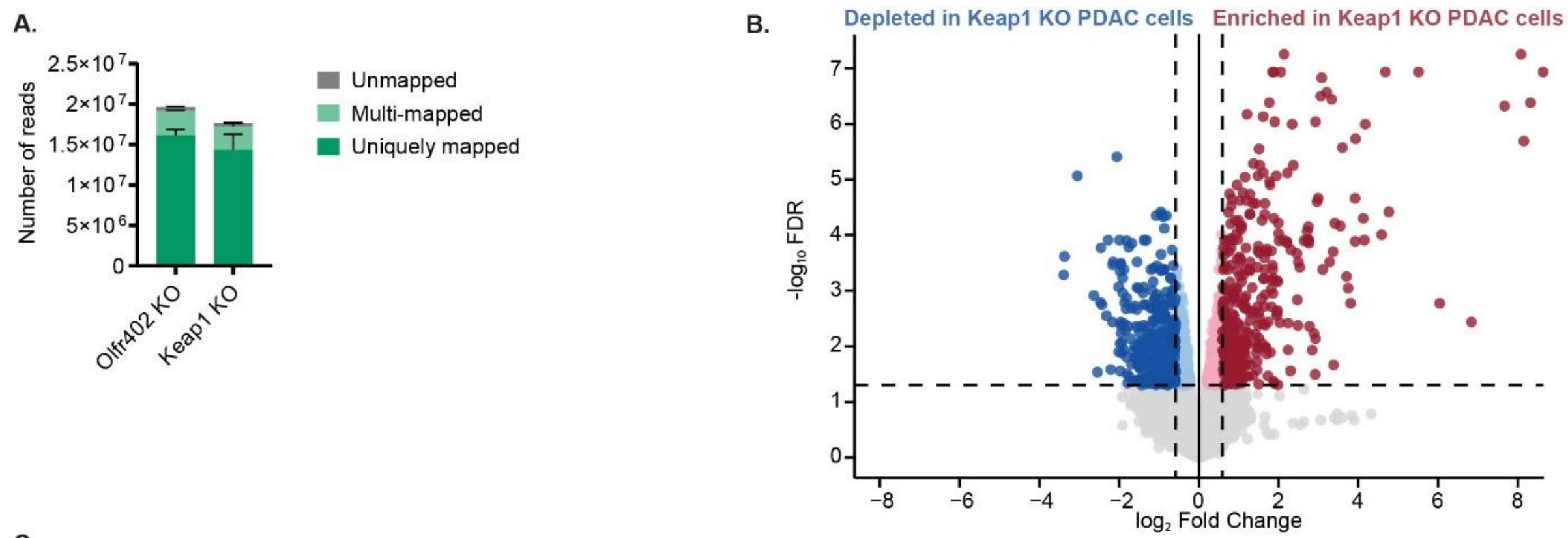

C.

Depleted in Keap1 KO PDAC cells

MSigDB Hallmark term	Size	NES	NOM p-value	FDR q-value
Interferon Gamma Response	109	-2.445	0	0
Interferon Alpha Response	54	-2.436	0	0
Inflammatory Response	83	-2.109	0	0
TNFA Signaling VIA NFKB	82	-2.106	0	0
Epithelial Mesenchymal Transition	63	-2.026	0	0
Coagulation	35	-1.966	0	0
KRAS Signaling UP	73	-1.874	0	0.000310041
Allograft Rejection	65	-1.853	0	0.000542571
IL6 JAK STAT3 Signaling	38	-1.833	0	0.000482285
Hypoxia	61	-1.829	0	0.000434057

Enriched in Keap1 KO PDAC cells

MSigDB Hallmark term	Size	NES	NOM p-value	FDR q-value
Xenobiotic Metabolism	36	2.52093	0	0
Reactive Oxygen Species Pathway	20	2.35896	0	0
Oxidative Phosphorylation	91	2.14969	0	0
MYC Targets V1	81	2.08381	0	0
MYC Targets V2	38	2.02866	0	0
Adipogenesis	61	1.94714	0	0
Fatty Acid Metabolism	47	1.69095	0	0.00473996
Bile Acid Metabolism	10	1.62308	0.005791506	0.00979263
Peroxisome	13	1.58617	0.005813953	0.0124936
MTORC1 Signaling	29	1.49458	0.001828154	0.02866359

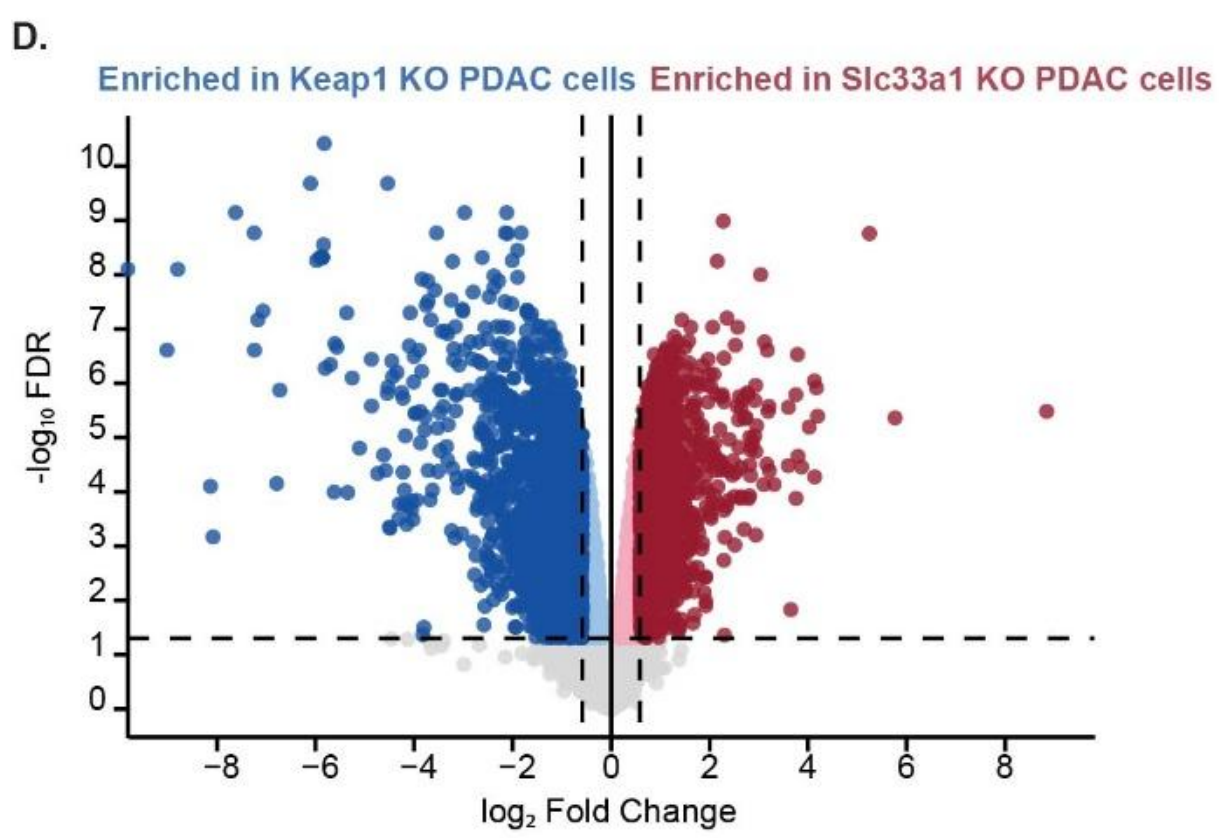

E.

MSigDB Hallmark term	Size	NES	NOM p-value	FDR q-value
MYC Targets V1	99	2.66034	0	0
Unfolded Protein Response	67	2.46557	0	0
E2F Targets	90	2.32497	0	0
MYC Targets V2	38	2.16408	0	0
G2M Checkpoint	61	2.06507	0	0
MTORC1 Signaling	67	1.91177	0	0.000453972
DNA Repair	53	1.52754	0	0.018288597
TNFA Signaling VIA NFKB	46	1.38732	0.002695418	0.056860025
PI3K AKT MTOR Signaling	29	1.31055	0.049261084	0.098259773
Oxidative Phosphorylation	52	1.30716	0.018018018	0.09097604

MSigDB Hallmark term	Size	NES	NOM p-value	FDR q-value
Reactive Oxygen Species Pathway	18	-2.1249	0	0
Xenobiotic Metabolism	54	-1.9925	0	0
Angiogenesis	12	-1.9643	0	0
Apical Surface	13	-1.8718	0	0.00079536
Bile Acid Metabolism	28	-1.8496	0	0.001060481
Interferon Gamma Response	79	-1.6151	0	0.017851422
Peroxisome	28	-1.5794	0.004975124	0.021058113
Interferon Alpha Response	49	-1.506	0.001680672	0.033935377
KRAS Signaling DN	27	-1.4403	0.026315789	0.057619442
Epithelial Mesenchymal Transition	52	-1.4266	0.007974482	0.060023198

Figure S16: Bulk tumor cell transcriptome profiling of *Keap1* KO PDAC cells. A) Bulk RNA sequencing read assignment for Control (*Olfr402*) or *Keap1* KO PDAC cells isolated from orthotopic tumors. The majority of reads are uniquely mapped. **B)** Volcano plot showing the differentially expressed genes of *Keap1* KO PDAC cells compared to control PDAC cells. Genes depicted in blue are depleted in *Keap1* KO cells compared to control KO cells. Genes depicted in red are enriched in *Keap1* KO cells compared to control KO cells. The cutoff for gene significance was set at a log fold change of |1.5| and an adjusted p-value of < 0.05. The y-axis depicts the -$\log_{10}$ FDR and the x-axis depicts the $\log_2$ fold change. **C)** GSEA analysis on differentially expressed genes in *Keap1* KO cells compared to control KO cells using the MSigDB Hallmark gene set. The top 10 pathways enriched (red) or depleted (blue) in *Keap1* KO cells compared to control KO cells are shown. **D)** Volcano plot showing the differentially expressed genes of *Keap1* KO PDAC cells compared to *Slc33a1* KO PDAC cells. Genes depicted in blue are enriched in *Keap1* KO cells compared to *Slc33a1* KO cells. Genes depicted in red are enriched in *Slc33a1* KO tumor cells compared to *Keap1* KO cells. The cutoff for gene significance was set at a log fold change of |1.5| and an adjusted p-value of < 0.05. The y-axis depicts the -$\log_{10}$ FDR and the x-axis depicts the $\log_2$ fold change.

E) GSEA analysis on differentially expressed genes in *Keap1* KO cells compared to *Slc33a1* KO cells using the MSigDB Hallmark gene set. The top 10 pathways enriched in Slc33a1 KO cells (red) or enriched in *Keap1* KO cells (blue) are shown.

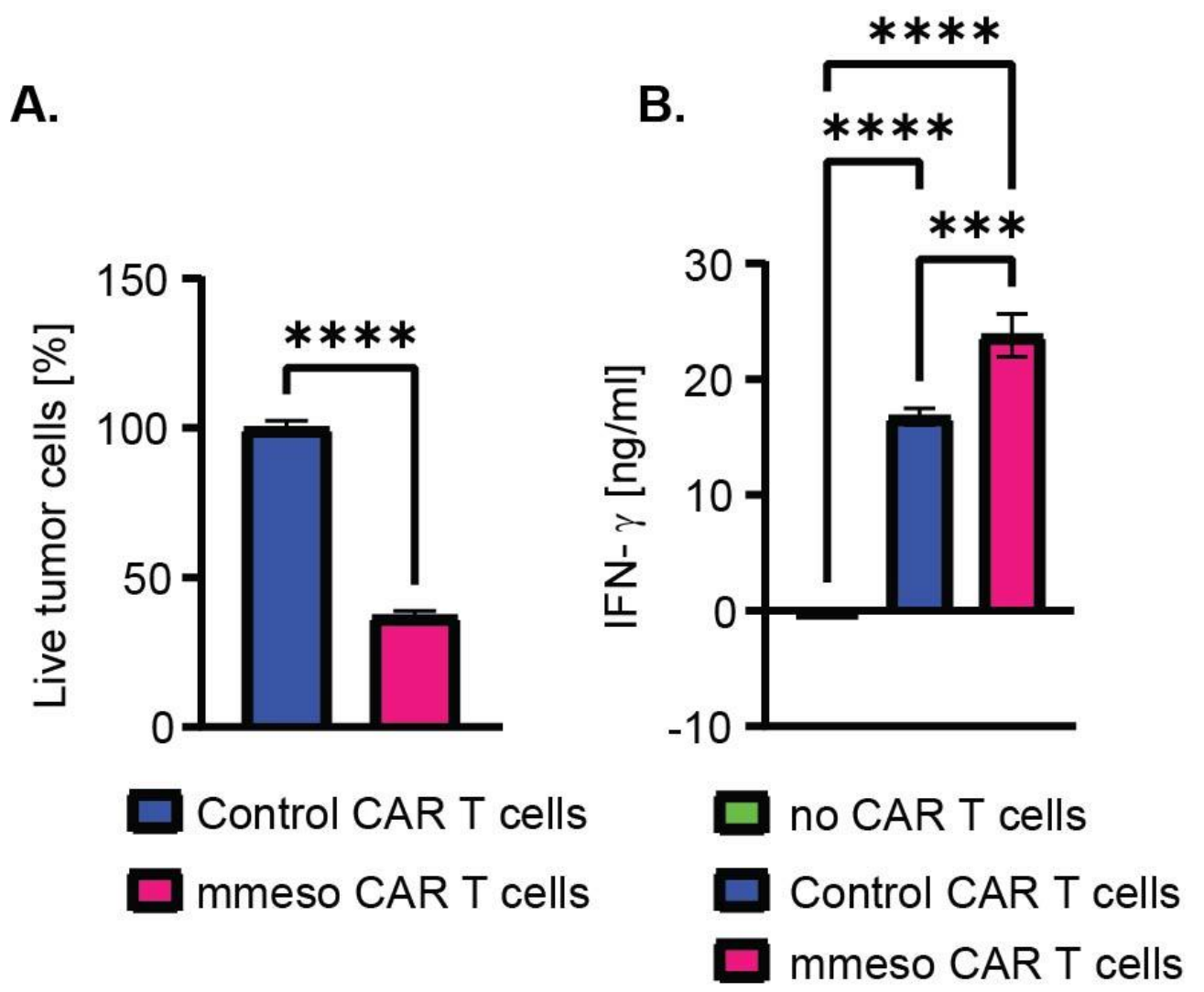

Figure S17: WT PDAC cells are efficiently killed by mmeso CAR T cells. A) A bar graph showing CAR T cell killing after incubation of mmeso CAR T cells or control (EGFRvIII) CAR T cells with PDAC cells (E:T ratio = 10:1) for 24 hours. All conditions were analyzed in triplicate. Error bars represent standard deviation. Statistical analysis with Ordinary One-Way ANOVA test (ns > 0.05, * ≤ 0.05, ** p ≤ 0.01, *** p ≤ 0.001, **** p ≤ 0.0001). **B)** A bar graph showing the average IFN-γ release by mmeso or control (EGFRvIII) CAR T cells during the CAR T cell cytotoxicity assays. All conditions were analyzed in triplicate and error bars represent standard deviation. Statistical analysis conducted using Ordinary One-Way ANOVA test (ns > 0.05, * ≤ 0.05, ** p ≤ 0.01, *** p ≤ 0.001, **** p ≤ 0.0001).

Supplemental tables

Table_S1.xlsx: Genes targeted by sgRNAs in SKY CRISPR library pools 1-16

Table_S2.xlsx: Genes targeted by sgRNAs in validation CRISPR library

Table_S3.xlsx: Classification of CRISPR screen hits

Table_S4.xlsx: Murine Nrf2-responsive genes

REFERENCES

1. L. Rahib, B. D. Smith, R. Aizenberg, A. B. Rosenzweig, J. M. Fleshman, L. M. Matrisian, Projecting cancer incidence and deaths to 2030: The unexpected burden of thyroid, liver, and pancreas cancers in the United States. *Cancer Res.* **74**, 2913–2921 (2014).

2. M. Brunner, Z. Wu, C. Krautz, C. Pilarsky, R. Grützmann, G. F. Weber, Current clinical strategies of pancreatic cancer treatment and open molecular questions. *Int. J. Mol. Sci.* **20**, 4543 (2019).

3. J. H. Park, I. Rivière, M. Gonen, X. Wang, B. Sénéchal, K. J. Curran, C. Sauter, Y. Wang, B. Santomasso, E. Mead, M. Roshal, P. Maslak, M. Davila, R. J. Brentjens, M. Sadelain, Long-term follow-up of CD19 CAR therapy in acute lymphoblastic leukemia. *N. Engl. J. Med.* **378**, 449–459 (2018).

4. M. Akce, M. Y. Zaidi, E. K. Waller, B. F. El-Rayes, G. B. Lesinski, The potential of CAR T cell therapy in pancreatic cancer. *Front. Immunol.* **9**, 2166 (2018).

5. A. Czaplicka, M. Lachota, L. Pączek, R. Zagożdżon, B. Kaleta, Chimeric antigen receptor T cell therapy for pancreatic cancer: A review of current evidence. *Cells* **13**, 101 (2024).

6. K. Drougkas, K. Karampinos, I. Karavolias, I.-A. Koumprentziotis, I. Ploumaki, E. Triantafyllou, I. Trontzas, E. Kotteas, Comprehensive clinical evaluation of CAR-T cell immunotherapy for solid tumors: A path moving forward or a dead end? *J. Cancer Res. Clin. Oncol.* **149**, 2709–2734 (2023).

7. M. C. Milone, J. Xu, S.-J. Chen, M. A. Collins, J. Zhou, D. J. Powell, J. J. Melenhorst, Engineering-enhanced CAR T cells for improved cancer therapy. *Nat. Cancer* **2**, 780–793 (2021).

8. R. M. Young, N. W. Engel, U. Uslu, N. Wellhausen, C. H. June, Next-generation CAR T-cell therapies. *Cancer Discov.* **12**, 1625–1633 (2022).

9. A. Kalbasi, A. Ribas, Tumour-intrinsic resistance to immune checkpoint blockade. *Nat. Rev. Immunol.* **20**, 25–39 (2020).

10. E. J. Orlando, X. Han, C. Tribouley, P. A. Wood, R. J. Leary, M. Riester, J. E. Levine, M. Qayed, S. A. Grupp, M. Boyer, B. De Moerloose, E. R. Nemecek, H. Bittencourt, H. Hiramatsu, J. Buechner, S. M. Davies, M. R. Verneris, K. Nguyen, J. L. Brogdon, H. Bitter, M. Morrissey, P. Pierog, S. Pantano, J. A. Engelman, W. Winckler, Genetic mechanisms of target antigen loss in CAR19 therapy of acute lymphoblastic leukemia. *Nat. Med.* **24**, 1504–1506 (2018).

11. E. Sotillo, D. M. Barrett, K. L. Black, A. Bagashev, D. Oldridge, G. Wu, R. Sussman, C. Lanauze, M. Ruella, M. R. Gazzara, N. M. Martinez, C. T. Harrington, E. Y. Chung, J. Perazzelli, T. J. Hofmann, S. L. Maude, P. Raman, A. Barrera, S. Gill, S. F. Lacey, J. J. Melenhorst, D. Allman, E. Jacoby, T. Fry, C. Mackall, Y. Barash, K. W. Lynch, J. M. Maris, S. A. Grupp, A. Thomas-Tikhonenko, Convergence of acquired mutations and alternative splicing of CD19 enables resistance to CART-19 immunotherapy. *Cancer Discov.* **5**, 1282–1295 (2015).

12. F. Braig, A. Brandt, M. Goebeler, H.-P. Tony, A.-K. Kurze, P. Nollau, T. Bumm, S. Böttcher, R. C. Bargou, M. Binder, Resistance to anti-CD19/CD3 BiTE in acute lymphoblastic leukemia may be mediated by disrupted CD19 membrane trafficking. *Blood* **129**, 100–104 (2017).

13. N. Singh, Y. G. Lee, O. Shestova, P. Ravikumar, K. E. Hayer, S. J. Hong, X. M. Lu, R. Pajarillo, S. Agarwal, S. Kuramitsu, E. J. Orlando, K. T. Mueller, C. R. Good, S. L. Berger, O. Shalem, M. D. Weitzman, N. V. Frey, S. L. Maude, S. A. Grupp, C. H. June, S. Gill, M. Ruella, Impaired death receptor signaling in leukemia causes antigen-independent resistance by inducing CAR T-cell dysfunction. *Cancer Discov.* **10**, 552–567 (2020).

14. A. Ramos, C. E. Koch, Y. Liu-Lupo, R. D. Hellinger, T. Kyung, K. L. Abbott, J. Fröse, D. Goulet, K. S. Gordon, K. P. Eidell, P. Leclerc, C. A. Whittaker, R. C. Larson, A. J. Muscato, K. B. Yates, J. Dubrot, J. G. Doench, A. Regev, M. G. Vander Heiden, M. V. Maus, R. T. Manguso, M. E. Birnbaum, M. T. Hemann, Leukemia-intrinsic determinants of CAR-T response revealed by iterative in vivo genome-wide CRISPR screening. *Nat. Commun.* **14**, 8048 (2023).

15. M. Boulch, M. Cazaux, A. Cuffel, M. V. Guerin, Z. Garcia, R. Alonso, F. Lemaître, A. Beer, B. Corre, L. Menger, C. L. Grandjean, F. Morin, C. Thieblemont, S. Caillat-Zucman, P.

Bousso, Tumor-intrinsic sensitivity to the pro-apoptotic effects of IFN-γ is a major determinant of CD4+ CAR T-cell antitumor activity. *Nat. Cancer* **4**, 968–983 (2023).

16. J. Dubrot, P. P. Du, S. K. Lane-Reticker, E. A. Kessler, A. J. Muscato, A. Mehta, S. S. Freeman, P. M. Allen, K. E. Olander, K. M. Ockerman, C. H. Wolfe, F. Wiesmann, N. H. Knudsen, H.-W. Tsao, A. Iracheta-Vellve, E. M. Schneider, A. N. Rivera-Rosario, I. C. Kohnle, H. W. Pope, A. Ayer, G. Mishra, M. D. Zimmer, S. Y. Kim, A. Mahapatra, H. Ebrahimi-Nik, D. T. Frederick, G. M. Boland, W. N. Haining, D. E. Root, J. G. Doench, N. Hacohen, K. B. Yates, R. T. Manguso, In vivo CRISPR screens reveal the landscape of immune evasion pathways across cancer. *Nat. Immunol.* **23**, 1495–1506 (2022).

17. K. E. de Visser, J. A. Joyce, The evolving tumor microenvironment: From cancer initiation to metastatic outgrowth. *Cancer Cell* **41**, 374–403 (2023).

18. M. Hidalgo, Pancreatic cancer. *N. Engl. J. Med.* **362**, 1605–1617 (2010).

19. P. Bailey, D. K. Chang, K. Nones, A. L. Johns, A.-M. Patch, M.-C. Gingras, D. K. Miller, A. N. Christ, T. J. C. Bruxner, M. C. Quinn, C. Nourse, L. C. Murtaugh, I. Harliwong, S. Idrisoglu, S. Manning, E. Nourbakhsh, S. Wani, L. Fink, O. Holmes, V. Chin, M. J. Anderson, S. Kazakoff, C. Leonard, F. Newell, N. Waddell, S. Wood, Q. Xu, P. J. Wilson, N. Cloonan, K. S. Kassahn, D. Taylor, K. Quek, A. Robertson, L. Pantano, L. Mincarelli, L. N. Sanchez, L. Evers, J. Wu, M. Pinese, M. J. Cowley, M. D. Jones, E. K. Colvin, A. M. Nagrial, E. S. Humphrey, L. A. Chantrill, A. Mawson, J. Humphris, A. Chou, M. Pajic, C. J. Scarlett, A. V. Pinho, M. Giry-Laterriere, I. Rooman, J. S. Samra, J. G. Kench, J. A. Lovell, N. D. Merrett, C. W. Toon, K. Epari, N. Q. Nguyen, A. Barbour, N. Zeps, K. Moran-Jones, N. B. Jamieson, J. S. Graham, F. Duthie, K. Oien, J. Hair, R. Grützmann, A. Maitra, C. A. Iacobuzio-Donahue, C. L. Wolfgang, R. A. Morgan, R. T. Lawlor, V. Corbo, C. Bassi, B. Rusev, P. Capelli, R. Salvia, G. Tortora, D. Mukhopadhyay, G. M. Petersen, Australian Pancreatic Cancer Genome Initiative, D. M. Munzy, W. E. Fisher, S. A. Karim, J. R. Eshleman, R. H. Hruban, C. Pilarsky, J. P. Morton, O. J. Sansom, A. Scarpa, E. A. Musgrove, U.-M. H. Bailey, O. Hofmann, R. L. Sutherland, D. A. Wheeler, A. J. Gill, R. A. Gibbs, J. V. Pearson, N. Waddell, A. V. Biankin, S. M. Grimmond, Genomic analyses identify molecular subtypes of pancreatic cancer. *Nature* **531**, 47–52 (2016).

20. S. Raghavan, P. S. Winter, A. W. Navia, H. L. Williams, A. DenAdel, K. E. Lowder, J. Galvez-Reyes, R. L. Kalekar, N. Mulugeta, K. S. Kapner, M. S. Raghavan, A. A. Borah, N. Liu, S. A. Väyrynen, A. D. Costa, R. W. S. Ng, J. Wang, E. K. Hill, D. Y. Ragon, L. K. Brais, A. M. Jaeger, L. F. Spurr, Y. Y. Li, A. D. Cherniack, M. A. Booker, E. F. Cohen, M. Y. Tolstorukov, I. Wakiro, A. Rotem, B. E. Johnson, J. M. McFarland, E. T. Sicinska, T. E. Jacks, R. J. Sullivan, G. I. Shapiro, T. E. Clancy, K. Perez, D. A. Rubinson, K. Ng, J. M. Cleary, L. Crawford, S. R. Manalis, J. A. Nowak, B. M. Wolpin, W. C. Hahn, A. J. Aguirre, A. K. Shalek, Microenvironment drives cell state, plasticity, and drug response in pancreatic cancer. *Cell* **184**, 6119–6137.e26 (2021).

21. W. L. Hwang, K. A. Jagadeesh, J. A. Guo, H. I. Hoffman, P. Yadollahpour, J. W. Reeves, R. Mohan, E. Drokhlyansky, N. Van Wittenberghe, O. Ashenberg, S. L. Farhi, D. Schapiro, P. Divakar, E. Miller, D. R. Zollinger, G. Eng, J. M. Schenkel, J. Su, C. Shiau, P. Yu, W. A. Freed-Pastor, D. Abbondanza, A. Mehta, J. Gould, C. Lambden, C. B. M. Porter, A. Tsankov, D. Dionne, J. Waldman, M. S. Cuoco, L. Nguyen, T. Delorey, D. Phillips, J. L. Barth, M. Kem, C. Rodrigues, D. Ciprani, J. Roldan, P. Zelga, V. Jorgji, J. H. Chen, Z. Ely, D. Zhao, K. Fuhrman, R. Fropf, J. M. Beechem, J. S. Loeffler, D. P. Ryan, C. D. Weekes, C. R. Ferrone, M. Qadan, M. J. Aryee, R. K. Jain, D. S. Neuberg, J. Y. Wo, T. S. Hong, R. Xavier, A. J. Aguirre, O. Rozenblatt-Rosen, M. Mino-Kenudson, C. F.-D. Castillo, A. S. Liss, D. T. Ting, T. Jacks, A. Regev, Single-nucleus and spatial transcriptome profiling of pancreatic cancer identifies multicellular dynamics associated with neoadjuvant treatment. *Nat. Genet.* **54**, 1178–1191 (2022).

22. K. L. Pitter, O. Grbovic-Huezo, S. Joost, A. Singhal, M. Blum, K. Wu, M. Holm, A. Ferrena, A. Bhutkar, A. Hudson, N. Lecomte, E. de Stanchina, R. Chaligne, C. A. Iacobuzio-Donahue, D. Pe’er, T. Tammela, Systematic comparison of pancreatic ductal adenocarcinoma models identifies a conserved highly plastic basal cell state. *Cancer Res.* **82**, 3549–3560 (2022).

23. A. Singhal, H. C. Styers, J. Rub, Z. Li, S. R. Torborg, J. Y. Kim, O. Grbovic-Huezo, H. Feng, Z. C. Tarcan, H. S. Ozkan, J. Hallin, O. Basturk, R. Yaeger, J. G. Christensen, D. Betel, Y. Yan, I. I. C. Chio, E. de Stanchina, T. Tammela, A classical epithelial state drives acute resistance to KRAS inhibition in pancreas cancer. *Cancer Discov.* **14**, 2122–2134 (2024).

24. Y. Gu, Z. Zhang, M. G. M. Camps, F. Ossendorp, R. H. Wijdeven, P. Ten Dijke, Genome-wide CRISPR screens define determinants of epithelial-mesenchymal transition mediated immune evasion by pancreatic cancer cells. *Sci. Adv.* **9**, eadf9915 (2023).

25. R. C. Larson, M. C. Kann, S. R. Bailey, N. J. Haradhvala, P. M. Llopis, A. A. Bouffard, I. Scarfő, M. B. Leick, K. Grauwet, T. R. Berger, K. Stewart, P. V. Anekal, M. Jan, J. Joung, A. Schmidts, T. Ouspenskaia, T. Law, A. Regev, G. Getz, M. V. Maus, CAR T cell killing requires the IFNγR pathway in solid but not liquid tumours. *Nature* **604**, 563–570 (2022).

26. K. R. Hagel, R. Arafeh, S. Gang, T. E. Arnoff, R. C. Larson, J. G. Doench, N. D. Mathewson, K. W. Wucherpfennig, M. V. Maus, W. C. Hahn, Systematic interrogation of tumor cell resistance to chimeric antigen receptor T-cell therapy in pancreatic cancer. *Cancer Res.* **83**, 613–625 (2023).

27. S. R. Hingorani, L. Wang, A. S. Multani, C. Combs, T. B. Deramaudt, R. H. Hruban, A. K. Rustgi, S. Chang, D. A. Tuveson, Trp53R172H and KrasG12D cooperate to promote chromosomal instability and widely metastatic pancreatic ductal adenocarcinoma in mice. *Cancer Cell* **7**, 469–483 (2005).

28. J. G. Doench, Am I ready for CRISPR? A user's guide to genetic screens. *Nat. Rev. Genet.* **19**, 67–80 (2018).

29. W. Li, H. Xu, T. Xiao, L. Cong, M. I. Love, F. Zhang, R. A. Irizarry, J. S. Liu, M. Brown, X. S. Liu, MAGeCK enables robust identification of essential genes from genome-scale CRISPR/Cas9 knockout screens. *Genome Biol.* **15**, 554 (2014).

30. T. Hart, A. H. Y. Tong, K. Chan, J. Van Leeuwen, A. Seetharaman, M. Aregger, M. Chandrashekhar, N. Hustedt, S. Seth, A. Noonan, A. Habsid, O. Sizova, L. Nedyalkova, R. Climie, L. Tworzyanski, K. Lawson, M. A. Sartori, S. Alibeh, D. Tieu, S. Masud, P. Mero, A. Weiss, K. R. Brown, M. Usaj, M. Billmann, M. Rahman, M. Constanzo, C. L. Myers, B. J. Andrews, C. Boone, D. Durocher, J. Moffat, Evaluation and design of genome-wide CRISPR/SpCas9 knockout screens. *G3* **7**, 2719–2727 (2017).

31. H. Ying, K. G. Elpek, A. Vinjamoori, S. M. Zimmerman, G. C. Chu, H. Yan, E. Fletcher-Sananikone, H. Zhang, Y. Liu, W. Wang, X. Ren, H. Zheng, A. C. Kimmelman, J. Paik, C. Lim, S. R. Perry, S. Jiang, B. Malinn, A. Protopopov, S. Colla, Y. Xiao, A. F. Hezel, N. Bardeesy, S. J. Turley, Y. A. Wang, L. Chin, S. P. Thayer, R. A. DePinho, Pten is a major tumor suppressor in pancreatic ductal adenocarcinoma and regulates an NF-κB-cytokine network. *Cancer Discov.* **1**, 158–169 (2011).

32. Q. Tu, J. Hao, X. Zhou, L. Yan, H. Dai, B. Sun, D. Yang, S. An, L. Lv, B. Jiao, C. Chen, R. Lai, P. Shi, X. Zhao, CDKN2B deletion is essential for pancreatic cancer development instead of unmeaningful co-deletion due to juxtaposition to CDKN2A. *Oncogene* **37**, 128–138 (2018).

33. S. Meril, M. Muhlbauer Avni, C. Lior, M. Bahlsen, T. Olender, A. Savidor, J. Krausz, H. Belhanes Peled, H. Birisi, N. David, S. Bialik, R. Scherz-Shouval, Y. Ben David, A. Kimchi, Loss of EIF4G2 mediates aggressiveness in distinct human endometrial cancer subpopulations with poor survival outcome in patients. *Oncogene* **43**, 1098–1112 (2024).

34. K. F. Harvey, X. Zhang, D. M. Thomas, The Hippo pathway and human cancer. *Nat. Rev. Cancer* **13**, 246–257 (2013).

35. J. Zhao, A. X. Chen, R. D. Gartrell, A. M. Silverman, L. Aparicio, T. Chu, D. Bordbar, D. Shan, J. Samanamud, A. Mahajan, I. Filip, R. Orenbuch, M. Goetz, J. T. Yamaguchi, M. Cloney, C. Horbinski, R. V. Lukas, J. Raizer, A. I. Rae, J. Yuan, P. Canoll, J. N. Bruce, Y. M. Saenger, P. Sims, F. M. Iwamoto, A. M. Sonabend, R. Rabadan, Immune and genomic correlates of response to anti-PD-1 immunotherapy in glioblastoma. *Nat. Med.* **25**, 462–469 (2019).

36. W. Peng, J. Q. Chen, C. Liu, S. Malu, C. Creasy, M. T. Tetzlaff, C. Xu, J. A. McKenzie, C. Zhang, X. Liang, L. J. Williams, W. Deng, G. Chen, R. Mbofung, A. J. Lazar, C. A. Torres-Cabala, Z. A. Cooper, P.-L. Chen, T. N. Tieu, S. Spranger, X. Yu, C. Bernatchez, M.-A. Forget, C. Haymaker, R. Amaria, J. L. McQuade, I. C. Glitza, T. Cascone, H. S. Li, L. N. Kwong, T. P. Heffernan, J. Hu, R. L. Bassett, M. W. Bosenberg, S. E. Woodman, W. W. Overwijk, G. Lizée, J. Roszik, T. F. Gajewski, J. A. Wargo, J. E. Gershenwald, L. Radvanyi,

M. A. Davies, P. Hwu, Loss of PTEN promotes resistance to T cell-mediated immunotherapy. *Cancer Discov.* **6**, 202–216 (2016).

37. F. Exposito, M. Redrado, M. Houry, K. Hastings, M. Molero-Abraham, T. Lozano, J. L. Solorzano, J. Sanz-Ortega, V. Adradas, R. Amat, E. Redin, S. Leon, N. Legarra, J. Garcia, D. Serrano, K. Valencia, C. Robles-Oteiza, G. Foggetti, N. Otegui, E. Felip, J. J. Lasarte, L. Paz-Ares, J. Zugazagoitia, K. Politi, L. Montuenga, A. Calvo, PTEN loss confers resistance to anti-PD-1 therapy in non-small cell lung cancer by increasing tumor infiltration of regulatory T cells. *Cancer Res.* **83**, 2513–2526 (2023).

38. T. Vidotto, C. M. Melo, E. Castelli, M. Koti, R. B. Dos Reis, J. A. Squire, Emerging role of PTEN loss in evasion of the immune response to tumours. *Br. J. Cancer* **122**, 1732–1743 (2020).

39. K. A. Lawson, C. M. Sousa, X. Zhang, E. Kim, R. Akthar, J. J. Caumanns, Y. Yao, N. Mikolajewicz, C. Ross, K. R. Brown, A. A. Zid, Z. P. Fan, S. Hui, J. A. Krall, D. M. Simons, C. J. Slater, V. De Jesus, L. Tang, R. Singh, J. E. Goldford, S. Martin, Q. Huang, E. A. Francis, A. Habsid, R. Climie, D. Tieu, J. Wei, R. Li, A. H. Y. Tong, M. Aregger, K. S. Chan, H. Han, X. Wang, P. Mero, J. H. Brumell, A. Finelli, L. Ailles, G. Bader, G. A. Smolen, G. A. Kingsbury, T. Hart, C. Kung, J. Moffat, Functional genomic landscape of cancer-intrinsic evasion of killing by T cells. *Nature* **586**, 120–126 (2020).

40. H. T. McMahon, E. Boucrot, Molecular mechanism and physiological functions of clathrin-mediated endocytosis. *Nat. Rev. Mol. Cell Biol.* **12**, 517–533 (2011).

41. B. Monfort, K. Want, S. Gervason, B. D’Autréaux, Recent advances in the elucidation of frataxin biochemical function open novel perspectives for the treatment of Friedreich’s ataxia. *Front. Neurosci.* **16**, 838335 (2022).

42. I. A. Dieterich, A. J. Lawton, Y. Peng, Q. Yu, T. W. Rhoads, K. A. Overmyer, Y. Cui, E. A. Armstrong, P. R. Howell, M. S. Burhans, L. Li, J. M. Denu, J. J. Coon, R. M. Anderson, L. Puglielli, Acetyl-CoA flux regulates the proteome and acetyl-proteome to maintain intracellular metabolic crosstalk. *Nat. Commun.* **10**, 3929 (2019).

43. Y. A. Hannun, L. M. Obeid, Sphingolipids and their metabolism in physiology and disease. *Nat. Rev. Mol. Cell Biol.* **19**, 175–191 (2018).

44. I.-H. Wang, T.-T. Huang, J.-L. Chen, L.-W. Chu, Y.-H. Ping, K.-W. Hsu, K.-H. Huang, W.-L. Fang, H.-C. Lee, C.-F. Chen, C.-C. Liao, R.-H. Hsieh, T.-S. Yeh, Mevalonate pathway enzyme HMGCS1 contributes to gastric cancer progression. *Cancer* **12**, 1088 (2020).

45. M. Soula, G. Unlu, R. Welch, A. Chudnovskiy, B. Uygur, V. Shah, H. Alwaseem, P. Bunk, V. Subramanyam, H.-W. Yeh, A. Khan, S. Heissel, H. Goodarzi, G. D. Victora, S. Beyaz, K. Birsoy, Glycosphingolipid synthesis mediates immune evasion in KRAS-driven cancer. *Nature* **633**, 451–458 (2024).

46. T. Nguyen, P. Nioi, C. B. Pickett, The Nrf2-antioxidant response element signaling pathway and its activation by oxidative stress. *J. Biol. Chem.* **284**, 13291–13295 (2009).

47. L. Baird, M. Yamamoto, The molecular mechanisms regulating the KEAP1-NRF2 pathway. *Mol. Cell. Biol.* **40**, e00099-20 (2020).

48. A.-M. Zavitsanou, R. Pillai, Y. Hao, W. L. Wu, E. Bartnicki, T. Karakousi, S. Rajalingam, A. Herrera, A. Karatza, A. Rashidfarrokhi, S. Solis, M. Ciampricotti, A. H. Yeaton, E. Ivanova, C. A. Wohlhieter, T. B. Buus, M. Hayashi, B. Karadal-Ferrena, H. I. Pass, J. T. Poirier, C. M. Rudin, K.-K. Wong, A. L. Moreira, K. M. Khanna, A. Tsirigos, T. Papagiannakopoulos, S. B. Koralov, KEAP1 mutation in lung adenocarcinoma promotes immune evasion and immunotherapy resistance. *Cell Rep.* **42**, 113295 (2023).

49. J. M. Matés, J. A. Campos-Sandoval, J. de Los Santos-Jiménez, J. Márquez, Glutaminases regulate glutathione and oxidative stress in cancer. *Arch. Toxicol.* **94**, 2603–2623 (2020).

50. M. Zucker, M. A. Perry, S. I. Gould, A. Elkrief, A. Safonov, R. Thummalapalli, M. Mehine, D. Chakravarty, A. R. Brannon, M. Ladanyi, P. Razavi, M. T. A. Donoghue, Y. R. Murciano-Goroff, K. Grigoriadis, N. McGranahan, M. Jamal-Hanjani, C. Swanton, Y. Chen, R. Shen, S. Chandarlapaty, D. B. Solit, N. Schultz, M. F. Berger, J. Chang, A. J. Schoenfeld, F. J. Sánchez-Rivera, E. Reznik, C. Bandlamudi, Pan-cancer analysis of biallelic inactivation in

tumor suppressor genes identifies KEAP1 zygosity as a predictive biomarker in lung cancer. *Cell* **188**, 851–867.e17 (2025).

51. J. W. Zagorski, A. E. Turley, H. E. Dover, K. R. VanDenBerg, J. R. Compton, C. E. Rockwell, The Nrf2 activator, tBHQ, differentially affects early events following stimulation of Jurkat cells. *Toxicol. Sci. Off. J. Soc. Toxicol.* **136**, 63–71 (2013).

52. K. Watanabe, Y. Luo, T. Da, S. Guedan, M. Ruella, J. Scholler, B. Keith, R. M. Young, B. Engels, S. Sorsa, M. Siurala, R. Havunen, S. Tähtinen, A. Hemminki, C. H. June, Pancreatic cancer therapy with combined mesothelin-redirected chimeric antigen receptor T cells and cytokine-armed oncolytic adenoviruses. *JCI Insight* **3**, 99573 (2018).

53. R. Paredes, R. Borea, F. Drago, A. Russo, G. Nigita, C. Rolfo, Genetic drivers of tumor microenvironment and immunotherapy resistance in non-small cell lung cancer: The role of KEAP1, SMARCA4, and PTEN mutations. *J. Immunother. Cancer* **13**, e012288 (2025).

54. R. Romero, F. J. Sánchez-Rivera, P. M. K. Westcott, K. L. Mercer, A. Bhutkar, A. Muir, T. J. González Robles, S. Lamboy Rodríguez, L. Z. Liao, S. R. Ng, L. Li, C. I. Colón, S. Naranjo, M. C. Beytagh, C. A. Lewis, P. P. Hsu, R. T. Bronson, M. G. Vander Heiden, T. Jacks, Keap1 mutation renders lung adenocarcinomas dependent on Slc33a1. *Nat. Cancer* **1**, 589–602 (2020).

55. A. Lister, T. Nedjadi, N. R. Kitteringham, F. Campbell, E. Costello, B. Lloyd, I. M. Copple, S. Williams, A. Owen, J. P. Neoptolemos, C. E. Goldring, B. K. Park, Nrf2 is overexpressed in pancreatic cancer: Implications for cell proliferation and therapy. *Mol. Cancer* **10**, 37 (2011).

56. M. C. Jonas, M. Pehar, L. Puglielli, AT-1 is the ER membrane acetyl-CoA transporter and is essential for cell viability. *J. Cell Sci.* **123**, 3378–3388 (2010).

57. S. Liu, M. Gad, C. Li, K. Cho, Y. Liu, K. Wangdu, V. Belay, A. Millet, H. Kojima, H. Sanford, M. Wölk, L. Urnavicius, M. Fedorova, G. J. Patti, E. V. Vinogradova, R. K. Hite, K. Birsoy, SLC33A1 exports oxidized glutathione to maintain endoplasmic reticulum redox homeostasis. *Nat. Cell Biol.* **28**, 903–914 (2026).

58. M. A. Farrugia, L. Puglielli, Nε-lysine acetylation in the endoplasmic reticulum—A novel cellular mechanism that regulates proteostasis and autophagy. *J. Cell Sci.* **131**, jcs221747 (2018).

59. D. Zhou, N. Chen, S. Huang, C. Song, Z. Zhang, Mechanistic insights into the acetyl-CoA recognition by SLC33A1. *Cell Discov.* **11**, 36 (2025).

60. M. M. Cooley, D. D. H. Thomas, K. Deans, Y. Peng, A. Lugea, S. J. Pandol, L. Puglielli, G. E. Groblewski, Deficient endoplasmic reticulum acetyl-CoA import in pancreatic acinar cells leads to chronic pancreatitis. *Cell. Mol. Gastroenterol. Hepatol.* **11**, 725–738 (2021).

61. W. Jia, Q. Gao, A. Han, H. Zhu, J. Yu, The potential mechanism, recognition and clinical significance of tumor pseudoprogression after immunotherapy. *Cancer Biol. Med.* **16**, 655–670 (2019).

62. Y. Ma, Q. Wang, Q. Dong, L. Zhan, J. Zhang, How to differentiate pseudoprogression from true progression in cancer patients treated with immunotherapy. *Am. J. Cancer Res.* **9**, 1546–1553 (2019).

63. X. Zhao, Y. Liu, G. Qin, Y. Ge, Q. Li, X. Chen, X. Tian, Y. Yu, J. Ren, Y. Zhang, Pseudoprogression in CAR-T cell therapy for solid tumor: Case report. *Front. Immunol.* **15**, 1504104 (2024).

64. X. Chen, C. Su, S. Ren, C. Zhou, T. Jiang, Pan-cancer analysis of KEAP1 mutations as biomarkers for immunotherapy outcomes. *Ann. Transl. Med.* **8**, 141 (2020).

65. Y. B. Hong, H. J. Kang, S. Y. Kwon, H. J. Kim, K. Y. Kwon, C. H. Cho, J.-M. Lee, B. V. S. Kallakury, I. Bae, Nrf2 regulates drug resistance in pancreatic cancer cells. *Pancreas* **39**, 463–472 (2010).

66. C. A. McIntyre, A. Grimont, J. Park, Y. Meng, W. J. Sisso, K. Seier, G. H. Jang, H. Walch, V. G. Aveson, D. J. Falvo, W. B. Fall, C. W. Chan, A. Wenger, B. L. Ecker, A. Pulvirenti, R. Gelfer, M. P. Zafra, N. Schultz, W. Park, E. M. O'Reilly, S. L. Houlihan, A. Alonso, E. Hissong, G. M. Church, C. E. Mason, D. Siolas, F. Notta, M. Gonen, L. E. Dow, W. R.

Jarnagin, R. Chandwani, Distinct clinical outcomes and biological features of specific KRAS mutants in human pancreatic cancer. *Cancer Cell* **42**, 1614–1629.e5 (2024).

67. A. J. Deen, S. Adinolfi, J. Härkönen, T. Patinen, X. Liu, T. Laitinen, P. Takabe, K. Kainulainen, S. Pasonen-Seppänen, L. M. Gawriyski, U. T. Arasu, I. Selvarajan, P. Mäkinen, H. Laitinen, E. Kansanen, M. U. Kaikkonen, A. Poso, M. Varjosalo, A.-L. Levonen, Oncogenic KEAP1 mutations activate TRAF2-NFκB signaling to prevent apoptosis in lung cancer cells. *Redox Biol.* **69**, 103031 (2024).

68. B. E. Hast, E. W. Cloer, D. Goldfarb, H. Li, P. F. Siesser, F. Yan, V. Walter, N. Zheng, D. N. Hayes, M. B. Major, Cancer-derived mutations in KEAP1 impair NRF2 degradation but not ubiquitination. *Cancer Res.* **74**, 808–817 (2014).

69. J. Alam, D. Stewart, C. Touchard, S. Boinapally, A. M. Choi, J. L. Cook, Nrf2, a Cap'n'Collar transcription factor, regulates induction of the heme oxygenase-1 gene. *J. Biol. Chem.* **274**, 26071–26078 (1999).

70. S. Martinez, S. Wu, M. Geuenich, A. Malik, R. Weber, T. Woo, A. Zhang, G. H. Jang, D. Dervovic, K. N. Al-Zahrani, R. Tsai, N. Fodil, P. Gros, S. Gallinger, G. G. Neely, F. Notta, A. Sendoel, K. Campbell, U. Elling, D. Schramek, In vivo CRISPR screens reveal SCAF1 and USP15 as drivers of pancreatic cancer. *Nat. Commun.* **15**, 5266 (2024).

71. C. S. Kubota, S. L. Myers, T. T. Seppälä, R. A. Burkhart, P. J. Espenshade, In vivo CRISPR screening identifies geranylgeranyl diphosphate as a pancreatic cancer tumor growth dependency. *Mol. Metab.* **85**, 101964 (2024).

72. D. E. Biancur, K. S. Kapner, K. Yamamoto, R. S. Banh, J. E. Neggers, A. S. W. Sohn, W. Wu, R. T. Manguso, A. Brown, D. E. Root, A. J. Aguirre, A. C. Kimmelman, Functional genomics identifies metabolic vulnerabilities in pancreatic cancer. *Cell Metab.* **33**, 199–210.e8 (2021).

73. X. G. Zhu, A. Chudnovskiy, L. Baudrier, B. Prizer, Y. Liu, B. N. Ostendorf, N. Yamaguchi, A. Arab, B. Tavora, R. Timson, S. Heissel, E. de Stanchina, H. Molina, G. D. Victora, H. Goodarzi, K. Birsoy, Functional genomics in vivo reveal metabolic dependencies of pancreatic cancer cells. *Cell Metab.* **33**, 211–221.e6 (2021).

74. V. Ngo, M. L. Duennwald, Nrf2 and oxidative stress: A general overview of mechanisms and implications in human disease. *Antioxidants* **11**, 2345 (2022).

75. M. Kryszczuk, O. Kowalczuk, Significance of NRF2 in physiological and pathological conditions an comprehensive review. *Arch. Biochem. Biophys.* **730**, 109417 (2022).

76. C. Tonelli, I. I. C. Chio, D. A. Tuveson, Transcriptional regulation by Nrf2. *Antioxid. Redox Signal.* **29**, 1727–1745 (2018).

77. R. Romero, V. I. Sayin, S. M. Davidson, M. R. Bauer, S. X. Singh, S. E. LeBoeuf, T. R. Karakousi, D. C. Ellis, A. Bhutkar, F. J. Sanchez-Rivera, L. Subbaraj, B. Martinez, R. T. Bronson, J. R. Prigge, E. E. Schmidt, C. J. Thomas, C. Goparaju, A. Davies, I. Dolgalev, A. Heguy, V. Allaj, J. T. Poirier, A. L. Moreira, C. M. Rudin, H. I. Pass, M. G. Vander Heiden, T. Jacks, T. Papagiannakopoulos, Keap1 loss promotes Kras-driven lung cancer and results in a dependence on glutaminolysis. *Nat. Med.* **23**, 1362–1368 (2017).

78. R.-F. Fan, K.-K. Tang, Z.-Y. Wang, L. Wang, Persistent activation of Nrf2 promotes a vicious cycle of oxidative stress and autophagy inhibition in cadmium-induced kidney injury. *Toxicology* **464**, 152999 (2021).

79. J. A. Hellyer, S. K. Padda, M. Diehn, H. A. Wakelee, Clinical implications of KEAP1-NFE2L2 mutations in NSCLC. *J. Thorac. Oncol.* **16**, 395–403 (2021).

80. D. Marinelli, M. Mazzotta, S. Scalera, I. Terrenato, F. Sperati, L. D'Ambrosio, M. Pallocca, G. Corleone, E. Krasniqi, L. Pizzuti, M. Barba, S. Carpano, P. Vici, M. Filetti, R. Giusti, A. Vecchione, M. Occhipinti, A. Gelibter, A. Botticelli, F. De Nicola, L. Ciuffreda, F. Goeman, E. Gallo, P. Visca, E. Pescarmona, M. Fanciulli, R. De Maria, P. Marchetti, G. Ciliberto, M. Maugeri-Saccà, KEAP1-driven co-mutations in lung adenocarcinoma unresponsive to immunotherapy despite high tumor mutational burden. *Ann. Oncol.* **31**, 1746–1754 (2020).

81. X. He, C. Xu, Immune checkpoint signaling and cancer immunotherapy. *Cell Res.* **30**, 660–669 (2020).

82. H. G. Hotz, H. A. Reber, B. Hotz, T. Yu, T. Foitzik, H. J. Buhr, G. Cortina, O. J. Hines, An orthotopic nude mouse model for evaluating pathophysiology and therapy of pancreatic cancer. *Pancreas* **26**, e89–e98 (2003).

83. F. J. Sánchez-Rivera, B. J. Diaz, E. R. Kastenhuber, H. Schmidt, A. Katti, M. Kennedy, V. Tem, Y.-J. Ho, J. Leibold, S. V. Paffenholz, F. M. Barriga, K. Chu, S. Goswami, A. N. Wuest, J. M. Simon, K. M. Tsanov, D. Chakravarty, H. Zhang, C. S. Leslie, S. W. Lowe, L. E. Dow, Base editing sensor libraries for high-throughput engineering and functional analysis of cancer-associated single nucleotide variants. *Nat. Biotechnol.* **40**, 862–873 (2022).

84. J. G. Doench, E. Hartenian, D. B. Graham, Z. Tothova, M. Hegde, I. Smith, M. Sullender, B. L. Ebert, R. J. Xavier, D. E. Root, Rational design of highly active sgRNAs for CRISPR-Cas9–mediated gene inactivation. *Nat. Biotechnol.* **32**, 1262–1267 (2014).

85. J. Fröse, J. Rowley, A. S. Farid, T. Rakhshandehroo, P. Leclerc, H. Mak, H. Allen, H. Moravej, L. Munaretto, L. Millan-Barea, E. Codet, H. Glockner, C. Jacobson, M. Hemann, M. Rashidian, Development of an antigen-based approach to noninvasively image CAR T cells in real time and as a predictive tool. *Sci. Adv.* **10**, eadn3816 (2024).

86. J. Acosta, G. A. Johnson, S. I. Gould, K. Dong, Y. Lendner, D. Detrés, O. Atwa, J. Bulkens, S. Gruber, M. E. Contreras, A. N. Wuest, V. K. Narendra, M. T. Hemann, F. J. Sánchez-Rivera, Multiplexed in vivo base editing identifies functional gene-variant-context interactions. bioRxiv 639770 [Preprint] (2025). https://doi.org/10.1101/2025.02.23.639770.

87. R Core Team, R: A Language and Environment for Statistical Computing. Vienna, Austria: R Foundation for Statistical Computing (2021); https://R-project.org.

88. H. Wickham, Welcome to the Tidyverse. *J. Open Source Softw.* **4**, 1686 (2019).

89. B. Wang, M. Wang, W. Zhang, T. Xiao, C.-H. Chen, A. Wu, F. Wu, N. Traugh, X. Wang, Z. Li, S. Mei, Y. Cui, S. Shi, J. J. Lipp, M. Hinterndorfer, J. Zuber, M. Brown, W. Li, X. S. Liu, Integrative analysis of pooled CRISPR genetic screens using MAGeCKFlute. *Nat. Protoc.* **14**, 756–780 (2019).

90. E. Dann, N. C. Henderson, S. A. Teichmann, M. D. Morgan, J. C. Marioni, Differential abundance testing on single-cell data using k-nearest neighbor graphs. *Nat. Biotechnol.* **40**, 245–253 (2022).
